# Synthesis of indolo[1,2-*c*]quinazolines from 2-alkynylaniline derivatives through Pd-catalyzed indole formation/cyclization with *N*,*N*-dimethylformamide dimethyl acetal

**DOI:** 10.3762/bjoc.14.218

**Published:** 2018-09-14

**Authors:** Antonio Arcadi, Sandro Cacchi, Giancarlo Fabrizi, Francesca Ghirga, Antonella Goggiamani, Antonia Iazzetti, Fabio Marinelli

**Affiliations:** 1 Dipartimento di Scienze Fisiche e Chimiche, Università di L’Aquila, Via Vetoio, 671010 Coppito (AQ), Italy; 2 Dipartimento di Chimica e Tecnologie del Farmaco, Sapienza, Università di Roma, P.le A. Moro 5, 00185, Rome, Italy; 3 Center for Life Nano Science@Sapienza, Istituto Italiano di Tecnologia, Viale Regina Elena 291, 00161 Rome, Italy

**Keywords:** arylboronic acids, DMFDMA, indoles, indoloquinazolines, quinazolines

## Abstract

An efficient strategy for the synthesis of 6-unsubstituted indolo[1,2-*c*]quinazolines is described. The Pd-catalyzed reaction of *o*-(*o*-aminophenylethynyl) trifluoroacetanilides with Ar–B(OH)_2_ afforded 2-(*o*-aminophenyl)-3-arylindoles, that were converted to 12-arylindolo[1,2-*c*]quinazolines by adding dimethylformamide dimethyl acetal (DMFDMA) to the reaction mixture after extractive work-up. This reaction outcome is different from the previously reported Pd-catalyzed sequential reaction of the same substrates with Ar–I, Ar–Br and ArN_2_^+^BF_4_^−^, that afforded 12-arylindolo[1,2-*c*]quinazolin-6(5*H*)-ones. Moreover, 12-unsubstituted indolo[1,2-*c*]quinazolines can be obtained both by reacting 2-(*o*-aminophenyl)indoles with DMFDMA or by sequential Pd-catalyzed reaction of *o*-(*o*-aminophenylethynyl)aniline with DMFDMA.

## Introduction

**Scheme 1:**
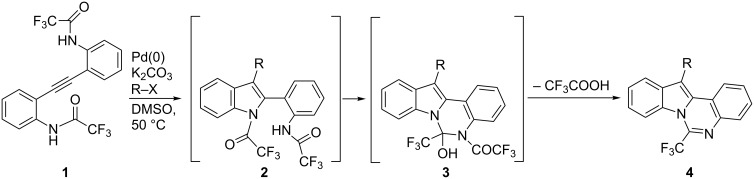
Synthesis of 6-trifluoromethyl-12-aryl(vinyl)indolo[1,2-*c*]quinazolines **4**.

Indoloquinazoline derivatives constitute an important class of compounds which exhibit a wide range of biological activities [1–4]. Then, extensive searches aimed at discovering pharmacologically active compounds encouraged the synthesis of some new products containing the indolequinazoline nucleus with the aim to discover novel drug candidates [5–8]. In particular, libraries of 6-substituted indolo[1,2-*c*]quinazolines were synthesized and exhibited good antimicrobial as well as notable antifungal activities [9–11]. Since the isolation of hinckdentine A, an unusual marine alkaloid from the bryozoan *Hincksinoflustra denticulata* collected off the eastern coast of Tasmania [12–13], indolo[1,2-*c*]quinazolines related to hinckdentine A received increasing attention as a source of new and useful pharmaceuticals. One well-established approach is based on the elaboration of a preformed indole ring, for example through cyclocondensation of 2-(*o*-aminophenyl)indoles with 2-cyanobenzothiazoles [14], aldehydes [9] and formic acid [15–16], or reactions of 2-(2-bromoaryl)-1*H*-indoles with aldehydes and aqueous ammonia [17] or with amino acids [18]. An alternative approach to indoloquinazolines is represented by sequential procedures that use 2-alkynylaniline derivatives as starting materials, via their conversion to 2-(*o*-aminophenyl)indole derivatives followed by further cyclization [19–22]. Our interest in this field led to the development of an original approach to 6-trifluoromethyl-12-aryl(vinyl)indolo[1,2-*c*]quinazolines **4** through sequential Pd-catalyzed reactions of bis(*o*-trifluoroacetamidophenyl)acetylene (**1**) with aryl or vinyl halides and triflates followed by cyclization reactions (Scheme 1) [19].

**Scheme 2:**
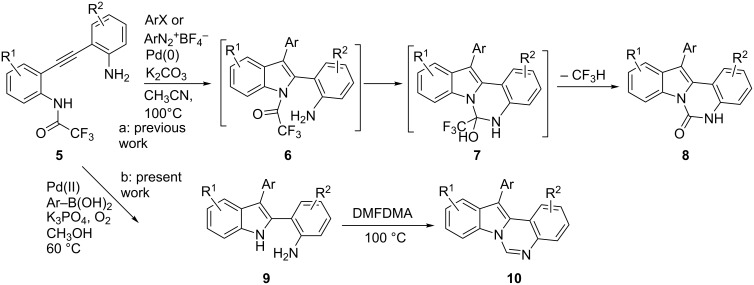
Present and previously reported reactions starting from **5**. DMFDMA: dimethylformamide dimethyl acetal.

The reaction, which tolerates a variety of important functional groups, likely involves the formation of the indole intermediates **2** (through aminopalladation/reductive elimination) [20–21] followed by cyclization to **3** and elimination of trifluoroacetic acid (Scheme 1). Later, a procedure that allows the introduction of a variety of substituents other than CF_3_ in the 6-position (without the substituent in the 12-position) was reported by Wang and co-workers [22]. More recently, we showed that the Pd-catalyzed reaction of *o*-(*o*-aminophenylethynyl) trifluoroacetanilides **5** with aryl halides and aryldiazonium salts led to the formation of 12-arylindolo[1,2-*c*]quinazolin-6(5*H*)-ones **8** in moderate to excellent yields (Scheme 2a) [23]. Very likely, under these reaction conditions, the palladium-catalyzed reaction of **5** gave intermediates **6**; then, cyclization to **7** followed by elimination of trifluoromethane afforded products **8**.

Since the unique molecular skeleton of hinckdentine A is constituted of a 6-unsubstituted indolo[1,2-*c*]quinazoline nucleus [12–13], we planned to modify our previous procedures to address the 6-unsubstituted-12-arylindolo[1,2-*c*]quinazolines **10**. We wish to report here that, under suitable reaction conditions, substrates **5** can be easily converted into target products **10** (Scheme 2b). Moreover, 12-unsubstituted analogues **13** are also available from **5** or from *o*-(*o*-aminophenylethynyl)anilines **15** (see below).

## Results and Discussion

**Scheme 3:**
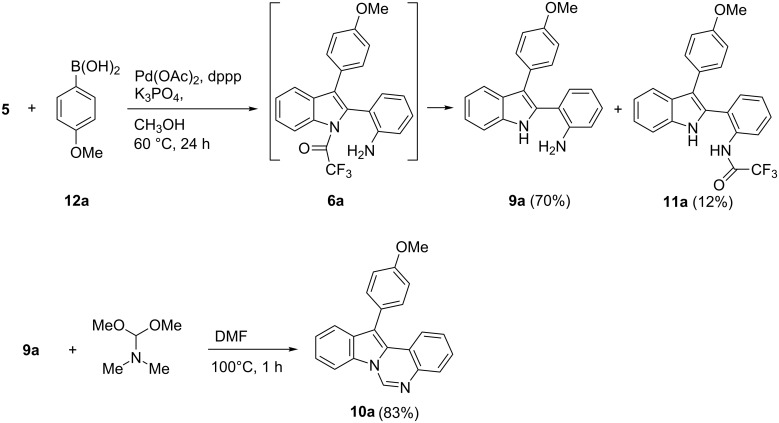
Two-step synthesis of 12-arylindolo[1,2-*c*]quinazoline **10a**.

We have previously reported that arylboronic acids **12** can be used in place of aryl halides in the Pd-catalyzed synthesis of indoles through aminopalladation/reductive elimination reaction from 2-alkynyltrifluoroacetanilides [24]. This reaction is carried out in MeOH at 60 °C in the presence of K_3_PO_4_ under an oxygen atmosphere. We then decided to react substrate **5** under these reaction conditions, in order to ascertain if the presence of methanol and base would result in the cleavage of the trifluoroacetylamide group after the formation of the indole ring, affording 2-(1*H*-indolo-2-yl)benzenamine derivatives **9** instead of products **8**. We were pleased to observe the formation of **9a** in good yields when **5** was reacted with 4-methoxyphenylboronic acid. A minor amount of product **11a** (likely deriving from a transamidation reaction [25] of initially formed intermediate **6a**) was also isolated. Then, **9a** was treated with dimethylformamide dimethyl acetal (DMFDMA) as a source of an electrophilic one-carbon unit at the formate oxidation level [26–27], affording 12-arylindolo[1,2-c]quinazoline **10a** in good yield (Scheme 3).

Based on this result, we tested an approach to target products **10** avoiding the isolation of intermediates **9**. After some experimentation, we found that the best results were obtained from the following procedure: 1) Pd-catalyzed reaction of **5** with ArB(OH)_2_/K_3_PO_4_ in MeOH at 60 °C; 2) heating of the reaction mixture at 100 °C to allow complete hydrolysis of byproducts **11**; 3) after extractive work-up, treatment of the crude reaction mixture with an excess of DMFDMA (5 equiv) [28] in DMF at 100 °C to obtain indoloquinazolines **10**. The results of this procedure, starting from a variety of *o*-(*o*-aminophenylethynyl)trifluoroacetanilides **5** and arylboronic acids **12**, are summarized in Table 1.

**Table 1:** Synthesis of 12-arylindolo[1,2-*c*]quinazolines **10**.^a^

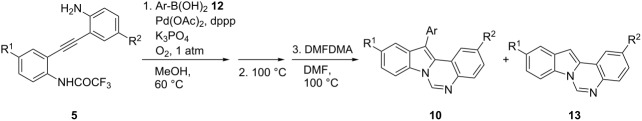							

Entry	R^1^	R^2^	**5**	Ar	Time (h)^b^	**10** (% yield)^c^	**13** (% yield)^c^

1	H	H	**5a**	4-MeO-C_6_H_4_-	3, 16, 16	**10a** (76)	–
2	H	H	**5a**	4-MeO-C_6_H_4_-	24, 18, 16	**10a** (49)^d^	–
3	H	H	**5a**	4-Me-C_6_H_4_-	5, 16, 16	**10b** (61)	–
4	H	H	**5a**	2-Me-C_6_H_4_-	5, 16, 16	**10j** (56)	**13j** (11)
5	H	H	**5a**	phenyl	24, 8, 12	**10c** (75)	–
6	H	H	**5a**	1-naphthyl	2, 16, 2	**10d** (60)	–
7	H	H	**5a**	4-CF_3_-C_6_H_4_-	4, 7, 16	**10e** (69)	–
8	H	H	**5a**	4-Br-C_6_H_4_-	2, 16, 6	**10f** (60)	–
9	H	H	**5a**	4-MeO_2_C-C_6_H_4_-	3, 28, 2	**10g** (59)	–
10	H	H	**5a**	4-Cl-C_6_H_4_-	6, 16, 3	**10h** (52)	**13h** (12)
11	H	H	**5a**	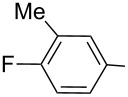	18, 16, 2	**10i** (65)	**13i** (12)
12	Me	H	**5b**	4-MeO-C_6_H_4_-	18, 6, 1	**10k** (63)	**13k** (5)
13	Me	H	**5b**	phenyl	24, 5, 4	**10l** (72)	–
14	Me	H	**5b**	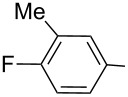	1, 16, 8	**10m** (76)	–
15	Me	COOMe	**5c**	4-MeO-C_6_H_4_-	5, 16, 3	**10n** (60)	–
16	H	Me	**5d**	4-Br-C_6_H_4_-	6, 16, 1.5	**10o** (58)	–
17	H	H	**5a**	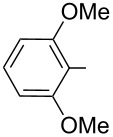	24, 24, 8	**10p** (12)	**13p** (38)

^a^Reactions were carried out at 60 °C on 0.345 mmol scale using 1.0 equiv of **5**, 2.0 equiv of **13**, 0.05 equiv of Pd(OAc)_2_, 0.05 equiv of dppp and 2 equiv of K_3_PO_4_ in MeOH (2 mL) under an atmosphere of O_2_; then 100 °C; work-up; then 5 equiv of DMFDMA in DMF (2 mL), 100 °C. ^b^Numbers refer to the 1st, 2nd and 3rd step, respectively. ^c^Isolated yields. ^d^Carried out under air atmosphere.

In some cases, 12-unsubstituted indolo[1,2-*c*]quinazoline derivatives **13** were also obtained as byproducts. Electron-rich arylboronic acids worked quite well (Table 1, entries 1, 3, and 4). However, an attempt to carry out the reaction under air, instead of under O_2_ atmosphere, resulted in a loss of efficiency (Table 1, entry 2). Good results were also observed with phenyl- and 1-naphthylboronic acids (Table 1, entries 5 and 6). Electron-poor arylboronic acids proved to be effective (Table 1, entries 7–10), and only (4-chlorophenyl)boronic acid gave a moderate yield of product (Table 1, entry 10).

Products substituted in the indole and quinazoline frameworks were also obtained (Table 1, entries 12–16). It is worth noting that the reaction tolerates a -Br substituent (Table 1, entries 8 and 16) which may serve as a useful handle for increasing molecular complexity through cross-coupling reactions. Only in the case of two *ortho*-substituents on the aryl residue of the boronic acid the reaction afforded the target product **10p** in low yield (Table 1, entry 17), and 12-unsubstituted derivative **13p** as the major product.

Then, with the aim of obtaining 12-unsubstituted indoloquinazolines **13** selectively, we tested the use of DMFDMA for the cyclization of 2-(*o*-aminophenyl)indoles **14**. This reaction required only a slight excess of DMFDMA (1.2 equiv), and afforded derivatives **13** as sole products in high yields (Table 2).

**Table 2:** Synthesis of 12-unsubstituted indolo[1,2-*c*]-quinazolines **13**.^a^

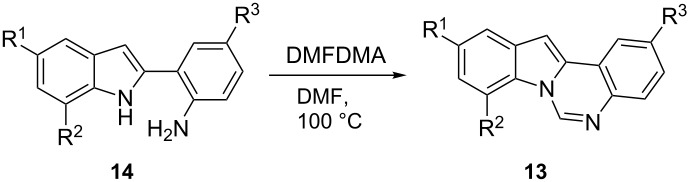						

Entry	R^1^	R^2^	R^3^	**14**	Time (h)	**13** (% yield)^b^

1	H	H	H	**14a**	5	**13a** (86)
2	Me	H	Me	**14b**	24	**13b** (87)
3	COOMe	H	COOMe	**14c**	6	**13c** (81)
4	H	H	Me	**14d**	25	**13d** (95)
5	Me	Me	H	**14e**	24	**13e** (73)

^a^Reactions were carried out at 100 °C on 0.481 mmol scale using 1.2 equiv of DMFDMA in DMF (2 mL). ^b^Isolated yields.

**Scheme 4:**
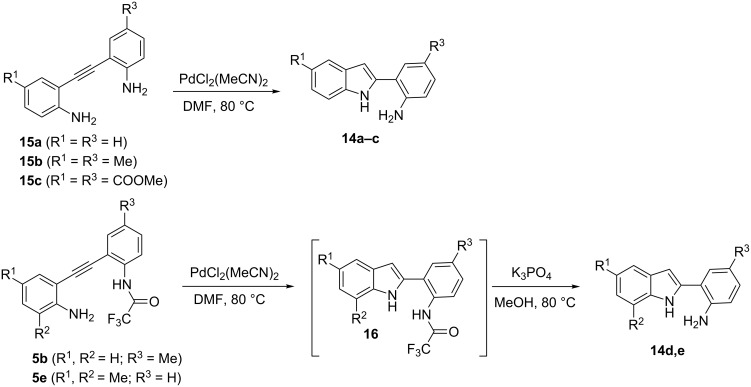
Synthesis of indoles **14**.

As described in Supporting Information File 1, indoles **14a**–**c** (R^1^ = R^3^, R^2^ = H) were obtained by cyclization of the corresponding *o*-(*o*-aminophenylethynyl)anilines **15a**–**c** with PdCl_2_(MeCN)_2_ [29–30]. Indoles **14d**,**e** (R^1^ ≠ R^3^) could not be obtained in such way (due to the lack of selectivity between the two different NH_2_ groups in the hydroamination reaction), and were obtained by cyclization of the corresponding *o*-(*o*-aminophenylethynyl) trifluoroacetanilides **5** with PdCl_2_(MeCN)_2_ (it was previously demonstrated that the selective formation of **16** occurs under these conditions [31]) followed by the hydrolysis of the COCF_3_ group of the crude product (Scheme 4).

**Scheme 5:**
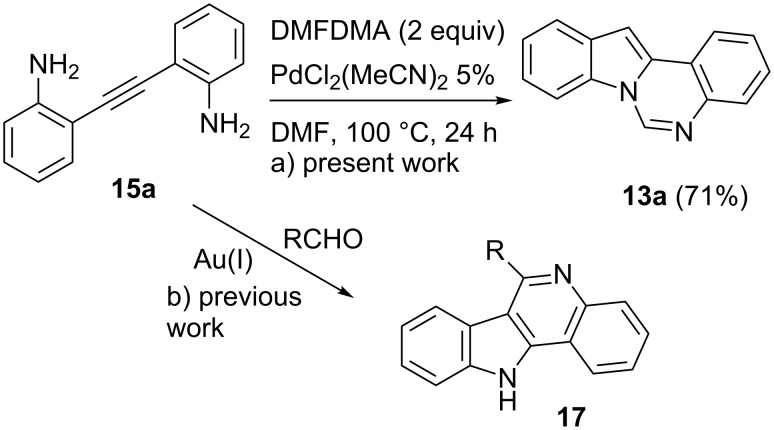
Sequential preparation of **13a** from **15a**.

Moreover, the palladium-catalyzed reaction of **15a** in the presence of DMFDMA led directly to the formation of quinazoline **13a** in 71% yield through a sequential process (Scheme 5a).

The selective formation of products **13** from **15** (and also from 3-unsubstituted indoles **14**) is not trivial, since the previously reported gold-catalyzed reaction of **15a** with aldehydes as electrophiles resulted in the divergent formation of 11*H*-indolo[3,2-*c*]quinolines **17** (Scheme 5b) [32] through functionalization of C-3 position of the indole ring instead of N-1.

The sequential reaction shown in Scheme 5, path a, probably occurs through cyclization of **15a** to indole **14a**, followed by the reaction with DMFDMA; however, an alternative path in which one amino group interact with DMFDMA to give a formamidine intermediate [27] before cyclization cannot be ruled out.

## Conclusion

We have reported here an efficient procedure for the preparation of 12-arylindolo[1,2-*c*]quinazolines **10** from *o*-(*o*-aminophenylethynyl) trifluoroacetanilides **5** and arylboronic acids, by avoiding the isolation of intermediate indoles **9**. Starting from indoles **14**, or from *o*-(*o*-aminophenylethynyl)aniline **15a**, selective formation of 12-unsubstituted[1,2-*c*]quinazolines **13** was accomplished, without competitive formation of products derived from C-3 functionalization at the indole moiety.

## Supporting Information

File 1 Experimental procedures, characterization data and copies of NMR spectra.
