# Supplementary material for: Synthesis of indolo[1,2-c]quinazolines from 2-alkynylaniline derivatives through Pd-catalyzed indole formation/cyclization with N,N-dimethylformamide dimethyl acetal
Source: Beilstein J Org Chem. 2018 Sep 14;14:2411–7. doi: 10.3762/bjoc.14.218 (PMC6142776; doi:10.3762/bjoc.14.218)
Supplement: File 1 — Experimental procedures, characterization data and copies of NMR spectra. [file Beilstein_J_Org_Chem-14-2411-s001.pdf]

Supporting Information

for

Synthesis of indolo[1,2-*c*]quinazolines from 2-alkynylaniline derivatives through Pd-catalyzed indole formation/cyclization with *N*,*N*-dimethylformamide dimethyl acetal

Antonio Arcadi[1], Sandro Cacchi[2], Giancarlo Fabrizi[2], Francesca Ghirga[3], Antonella Goggiamani[2], Antonia Iazzetti*[2] and Fabio Marinelli*[1]

Address: [1]Dipartimento di Scienze Fisiche e Chimiche, Università di L'Aquila, Via Vetoio, 671010 Coppito (AQ), Italy, [2]Dipartimento di Chimica e Tecnologie del Farmaco, Sapienza, Università di Roma, P.le A. Moro 5, 00185, Rome, Italy and [3]Center for Life Nano Science@Sapienza, Istituto Italiano di Tecnologia, Viale Regina Elena 291, 00161 Rome, Italy

*Corresponding author

Email: Fabio Marinelli - fabio.marinelli@univaq.it

Experimental procedures, characterization data and copies of NMR spectra

Contents

General information:	S2
General procedure:	S3
Characterization data (**5a–d; 14a–e; 9a)**:	S4
Characterization data (**10a–p; 13a–e)**:	S7
References	S15
Copies of NMR spectra:	S16

General information

Melting points are uncorrected. All of the reagents, catalysts, and solvents are commercially available and were used as purchased, without further purification. Starting materials were purified on axially compressed columns, packed with SiO_2 25–40 μm, connected to a preparative pump for solvent delivery and to a refractive index detector, and eluting with *n*-hexane/EtOAc mixtures. Reaction products were purified by flash chromatography using SiO_2 as stationary phase, eluting with *n*-hexane/ethyl acetate or $MeOH/CHCl_3$ mixtures. ^{1}H NMR (400.13 MHz), ^{13}C NMR (100.6 MHz) and ^{19}F NMR (376.5 MHz) spectra were recorded with a Bruker Avance 400 spectrometer. Splitting patterns are designed as s (singlet), d (doublet), t (triplet), q (quartet), m (multiplet), or bs (broad singlet). IR spectra were recorded with a Jasco FT/IR-430 spectrometer. HRMS spectra were recorded with Orbitrap Exactive Mass spectrometer with ESI source.

The appropriate *o*-(*o*-aminophenylethynyl) trifluoroacetanilides **5** and *o*-(*o*-aminophenylethynyl)anilines **15** were prepared, usually in high yields, via a Sonogashira cross-coupling of 2-iodotrifluoroacetanilides or 2-iodoaniline with 2-ethynylanilines.[1]

2-(*o*-Aminophenyl)indoles **14** were synthesized through two different procedures depending on the substitution of the indole derivatives: indoles **14a–c** ($R^1 = R^3$, R^2 = H) were prepared via palladium-catalyzed cyclization of the corresponding *o*-(*o*-aminophenylethynyl)aniline **15a–c** (Scheme 1).[2] Indoles **14d**,**e** ($R^1 \neq R^3$) were obtained by cyclization of the corresponding *o*-(*o*-aminophenylethynyl) trifluoroacetanilides **5** with $PdCl_2(MeCN)_2$ [2] followed by hydrolysis of $-COCF_3$ on the crude (Scheme 2).

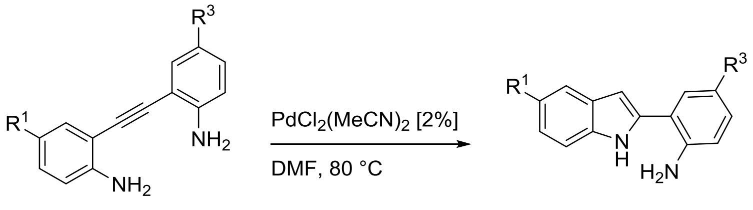

15a ($R^1 = R^3$ = H)
15b ($R^1 = R^3$ = Me)
15c ($R^1 = R^3$ = COOMe)

14a-c

Scheme S1: Preparation of **14a–c**.

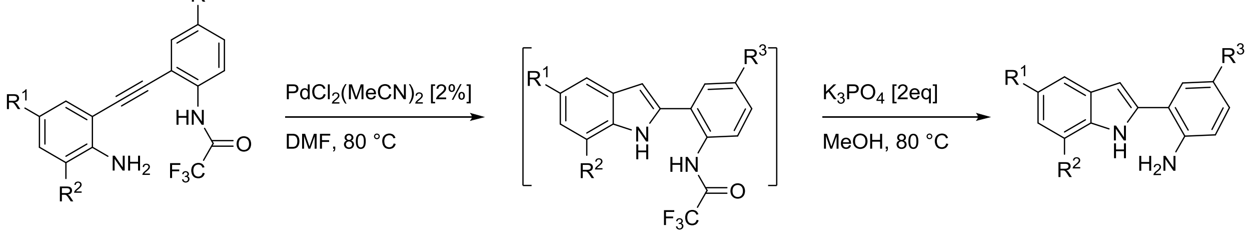

5b (R^1, R^2 = H; R^3 = Me)
5e (R^1, R^2 = Me; R^3 = H)

16

14d-e

Scheme S2: Preparation of **14d,e**.

Experimental procedures

Typical procedure for preparation of 12-(4-methoxyphenyl)indolo[1,2-*c*]quinazolines 10a–p. Preparation of 10a.

In a 50 mL Carousel Tube Reactor (Radely Discovery Technology) containing a magnetic stirring bar $Pd(OAc)_2$ (4.0 mg, 0.018 mmol) and dppp (7.4 mg, 0.018 mmol) were dissolved at room temperature with 1 mL of anhydrous MeOH. Then 2-((2-aminophenyl)ethynyl))trifluoroacetanilide (**5a**, 105.0 mg, 0.345 mmol), 4-methoxyphenyl-boronic acid (**12a**, 104.8 mg, 0.690 mmol), K_3PO_4 (146.4 mg, 0.690 mmol) and 1.0 mL of MeOH were added. The mixture was stirred at 60 °C under oxygen atmosphere until conversion of *o*-(*o*-aminophenylethynyl) trifluoroacetanilide (**5a**) into indole **11a** was completed (3 h). Then, in order to allow complete hydrolysis of **11a** to **9a**, the reaction mixture was warmed at 100 °C for 16 h, cooled to room temperature, diluted with ether and washed with a satured solution of $NaHCO_3$. The organic extract was dried over Na_2SO_4 and concentrated under reduced pressure. The residue was dissolved in 2 mL of DMF and DMFDMA (230 μL, 1.725 mmol) was added. The mixture was stirred at 100 °C for 8 h, cooled to room temperature, diluted with ether, washed with brine, dried over Na_2SO_4 and concentrated under reduced pressure. The residue was purified by flash chromatography (silica gel, *n*-hexane/EtOAc 85:15 v/v) to afford 12-(4-methoxyphenyl)indolo[1,2-*c*]quinazoline **10a** (85.0 mg, 76%).

Typical procedure for preparation of indolo[1,2-*c*]quinazolines 13a-d. Preparation of 13d.

To a solution of 2-(1*H*-indol-2-yl)-4-methylaniline (**14d**, 76.7 mg, 0.345 mmol) in 2 mL of DMF, DMFDMA (91 μL, 0.690 mmol) was added; the mixture was stirred at 100 °C. After 25 h, the reaction mixture was cooled to room temperature, diluted with ether and washed with a saturated solution of $NaHCO_3$. The organic layer was dried over Na_2SO_4 and concentrated under reduced pressure. The residue was purified by flash chromatography (silica gel, *n*-hexane/EtOAc 85/15 v/v) to afford 2-methylindolo[1,2-*c*]quinazoline **13d** (76.1 mg, 95%).

Sequential preparation of indolo[1,2-*c*]quinazoline 13a from 15a

To a stirred solution of $PdCl_2(MeCN)_2$ (6.2 mg, 0.024 mmol) in 2 mL of DMF *o*-(*o*-aminophenylethynyl)aniline (**15a**, 100 mg, 0.481 mmol) and DMFDMA (128 μL, 0.962 mmol) were added; the mixture was stirred at 100 °C under N_2 atmosphere for 24 h. Then the reaction mixture was cooled to room temperature, diluted with ether and washed with a saturated solution of $NaHCO_3$. The organic layer was dried over Na_2SO_4 and concentrated under reduced pressure. The residue was purified by flash chromatography (silica gel, *n*-hexane/EtOAc 80/20v/v) to afford indolo[1,2-*c*]quinazoline **13a** (74.8 mg, 71%).

Characterization data (5a–d; 14a–e; 9a)

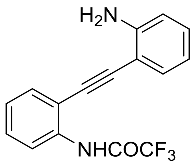

***N*-(2-((2-Aminophenyl)ethynyl)phenyl)-2,2,2-trifluoroacetamide (5a)** [3]

Pale yellow powder, **yield**: 82%; mp: 100-102 °C; **^{1}H NMR (CDCl$_3$):** δ 8.87 (bs, 1H), 8.39 (d, *J* = 7.6 Hz, 1H), 7.58 (d, *J* = 7.6 Hz, 1H), 7.46-7.43 (m, 2H), 7.36-7.23 (m, 2H), 6.77 (d, *J* = 4.0 Hz, 2H), 4.29 (bs, 2H); **^{13}C NMR (CDCl$_3$):** δ 154.5 (q, J_{CF} = 37 Hz), 147.9, 135.8, 132.2, 131.7, 130.8, 129.8, 126.2, 120.9, 119.8, 116.0 (q, J_{CF} = 241 Hz), 114.3, 113.7, 106.3, 94.7, 88.1; **^{19}F NMR (CDCl$_3$):** δ -75.7.

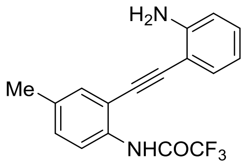

***N*-(2-((2-Aminophenyl)ethynyl)-4-methylphenyl)-2,2,2-trifluoroacetamide (5b)**[1b]

White powder, **yield**: 67%; mp: 141-143 °C; **^{1}H NMR (DMSO-*d$_6$*):** δ 11.17 (bs, 1H), 7.57 (s, 1H), 7.35 (d, *J* = 8.4 Hz, 1H), 7.27 (d, *J* = 8.0 Hz, 1 H), 7.18 (d, *J* = 7.2 Hz, 1H), 7.09 (t, *J* = 7.2 Hz, 1H), 6.73 (d, *J* = 8.4 Hz, 1H), 6.55 (t, *J* = 7.2 Hz, 1H), 5.56 (bs, 2H), 2.35 (s, 3H); **^{13}C NMR (DMSO-*d$_6$*):** δ 155.7 (q, J_{CF} = 36 Hz), 150.2, 137.6, 133.1, 132.9, 132.2, 130.6, 130.0, 126.9, 120.5, 116.6 (q, $J_{CF\,=}$ 287 Hz), 116.2, 114.4, 105.5, 92.3, 90.5, 20.8. **^{19}F NMR (DMSO-*d$_6$*):** δ: -75.6.

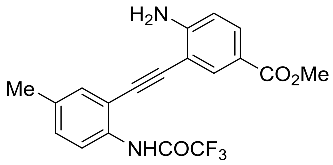

Methyl 4-amino-3-((5-methyl-2-(2,2,2-trifluoroacetamido)phenyl)ethynyl)benzoate (5c)

Pale yellow powder, **yield**: 37%; mp: 161-163 °C; **^{1}H NMR (CDCl$_3$):** δ 8.71 (bs, 1H), 8.20 (d, *J* = 8.4 Hz, 1H), 8.07 (d, *J* = 2.0 Hz, 1H), 7.88 (dd, J_1 = 8.8 Hz, J_2 = 1.8 Hz, 1H), 7.39 (s, 1H), 7.25 (d, *J* = 8.4 Hz, 1H), 6.75 (d, *J* = 8.4 Hz, 1H), 4.72 (bs, 2H), 3.90 (s, 3 H), 2.38 (s, 3H); **^{13}C NMR (CDCl$_3$):** δ 166.3, 154.5 (q, J_{CF} = 37 Hz), 151.4, 135.7, 134.6, 133.4, 132.3, 132.2, 130.8, 120.1, 119.8, 115.8 (q, J_{CF} = 287 Hz), 113.7, 113.5, 105.6, 92.8, 88.8, 51.9, 20.8.

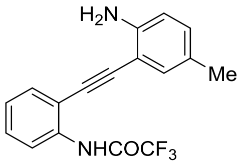

***N*-(2-((2-Amino-5-methylphenyl)ethynyl)phenyl)-2,2,2-trifluoroacetamide (5d)** [1b]

White powder, **yield**: 70%; mp :137-139 °C; **^{1}H NMR ($CDCl_3$):** δ 8.88 (bs, 1 H), 8.39 (d, J = 8,4 Hz, 1H), 7.57 (dd, J_1 = 7.6 Hz, J_2 = 1.2 Hz, 1H), 7.50-7.38 (m, 1H), 7.32-7.22 (m, 1H), 7.18 (s, 1H), 7.05 (dd, J_1 = 8.0 Hz, J_2 = 1.6 Hz, 1H), 6.70 (d, J = 8.0 Hz, 1H), 4.15 (bs, 2H), 2.28 (s, 3H); **^{13}C NMR ($CDCl_3$):** δ 154.5 (q, J = 37.5 Hz), 145.6, 135.7, 132.2, 131.8, 131.7, 129.7, 127.6, 125.6, 119.8, 115.7 (q, J = 287 Hz), 115.0, 113.8, 106.3, 95.0, 87.8, 20.3. **^{19}F NMR ($CDCl_3$):** δ -75.7.

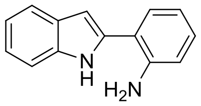

2-(1*H*-Indol-2-yl)aniline (14a) [4]

Pale yellow powder, **yield**: 82%; lit [4] mp: 163-165 °C; mp: 166-168 °C; **^{1}H NMR ($CDCl_3$):** δ 8.47 (bs, 1H), 7.67 (d, J = 7.2 Hz, 1H), 7.44-7.40 (m, 2H), 7.25-7.15 (m, 3H), 6.91-6.83 (m, 2H), 6.75 (s, 1H), 4.12 (bs, 2H); **^{13}C NMR ($CDCl_3$):** δ 144.0, 136.1, 135.9, 129.2, 129.1, 128.9, 122.1, 120.4, 120.2, 119.1, 118.8, 116.6, 110.8, 101.6.

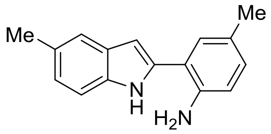

4-Methyl-2-(5-methyl-1*H*-indol-2-yl)aniline (14b) [5]

Beige powder, **yield**: 63%; lit [5] mp: 195-197 °C; mp: 194-196 °C; **^{1}H NMR ($CDCl_3$):** δ 8.33 (bs, 1H), 7.34 (s, 1H), 7.20 (t, J = 7.8 Hz, 1H), 7.13 (d, J = 1.6 Hz, 1H), 6.95 (dd, J_1 = 8.4 Hz, J_2 = 1.2 Hz, 1H), 6.91 (dd, J_1 = 8.4 Hz, J_2 = 1.6 Hz, 1H), 6.65 (d, J = 8.0 Hz, 1H), 6.55 (s, 1H), 3.84 (bs, 2H), 2.38 (s, 3H), 1.22 (s, 3H); **^{13}C NMR ($CDCl_3$):** δ 141.3, 136.2, 134.5, 129.54, 129.49, 129.3, 129.2, 128.5, 123.7, 120.0, 119.1, 116.9, 110.4, 101.0, 21.5, 20.5.

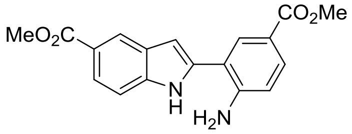

Methyl 2-(2-amino-5-(methoxycarbonyl)phenyl)-1*H*-indole-5-carboxylate (14c)

Pale yellow powder, **yield**: 82%; mp: 198-200 °C; **^{1}H NMR ($CDCl_3$):** δ 8.71 (bs, 1H), 8.42 (s, 1H), 8.08 (d, J = 1.6 Hz, 1H), 7.95 (dd, J_1 = 8.4 Hz, J_2 = 1.6 Hz, 1H), 7.87 (dd, J_1 = 8.4

Hz, J_2 = 2.0 Hz, 1H), 7.45 (d, J = 8.4 Hz, 1H), 6.85-6.80 (m, 2H), 4.62 (bs, 2H), 3.96 (s, 3H), 3.90 (s, 3H); **^{13}C NMR (CDCl$_3$):** δ 168.1, 166.9, 148.5, 138.8, 135.9,131.16, 131.11, 128.4, 123.9, 123.4, 122.4, 120.1, 116.9, 115.3, 110.6, 102.9, 51.9, 51.8.

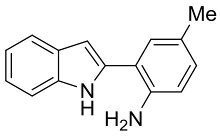

2-(1*H*-Indol-2-yl)-4-methylaniline (14d) [5]

Off-white powder, **yield**: 72%; lit [5] mp: 162-163 °C; mp: 165-167 °C; **^{1}H NMR (DMSO d_6):** δ 11.2 (s, 1H), 7.52 (d, J = 7.6, Hz 1H), 7.38 (d, J = 8.0 Hz, 1H), 7.20 (s, 1H), 7.05 (t, J = 7.4 Hz, 1H), 7.00 (t, J = 7.4 Hz 1H), 6.89 (d, J = 8.0 Hz, 1H), 6.74 (d, J = 8.4 Hz, 1H), 6.66 (s, 1H), 4.98 (s, 2H), 2.23 (s, 3H); **^{13}C NMR (DMSO d_6):** δ 143.6, 136.69, 136.68, 129.4, 129.1, 125.4, 121.4, 120.1, 119.4, 117.5, 116.5, 111.5, 100.4, 20.6.

2-(5,7-Dimethyl-1*H*-indol-2-yl)aniline (14e)

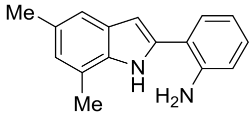

Beige powder, **yield**: 80%; mp: 192-194 °C; **^{1}H NMR (CDCl$_3$):** δ 8.27 (bs, 1H), 7.43 (dd, J_1 = 7.6 Hz, J_2 = 1.6 Hz, 1H), 7.32 (s, 1H), 7.21 (td, J_1 = 7.7 Hz, J_2 = 1.2 Hz, 1H), 6.92-6.88 (m, 2H), 6.84 (d, J = 8.0 Hz, 1H), 6.67 (s, 1H), 4.13 (bs, 2H), 2.52 (s, 3H), 2.47 (s, 3H); **^{13}C NMR (CDCl$_3$):** δ 144.1, 135.6, 134.1, 129.7, 129.2, 128.9, 128.7, 124.5, 119.7, 119.1, 118.9, 117.7, 116.4, 101.7, 21.4, 16.7.

2-(3-(4-Methoxyphenyl)-1*H*-indol-2-yl)aniline (9a)

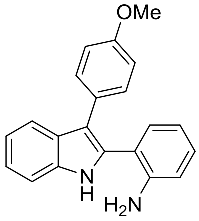

Brown oil, **yield**: 70%; mp: 155-157 °C; **^{1}H NMR (CDCl$_3$):** δ 8.29 (bs, 1H), 7.84 (d, J = 8.0 Hz, 1H), 7.41(d, J = 8.8 Hz, 3H),7.42-7.26 (m, 2H), 7.23-7.19 (m, 2H), 6.92 (d, J = 8.8 Hz, 2H), 6.82 (t, J = 7.2 Hz, 1H), 6.71 (d, J = 8.0 Hz, 1H), 3.84 (s, 3H), 3.75 (bs, 2H); **^{13}C NMR (CDCl$_3$):** δ.158.0, 144.6, 136.0, 131.7, 131.3, 129.9, 129.6, 127.7, 127.3, 122.5, 120.3, 119.5, 118.6, 118.4, 116.1, 114.9, 114.1, 111.0, 55.2.

Characterization data (10a–p; 13a–e)

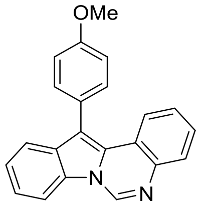

12-(4-Methoxyphenyl)indolo[1,2-*c*]quinazoline (10a)
Pale yellow powder, **yield**: 76%, mp: 135-136 °C; **IR (KBr)**: 2930, 2858, 2828, 1614, 1556, 1455 cm^{-1}; **^{1}H NMR ($CDCl_3$)**: δ 9.16 (s, 1H), 8.02 (d, *J* = 7.9 Hz, 1H), 7.82 (t, *J* = 7.0 Hz, 2H), 7.63 (d, *J* = 7.7 Hz, 1H), 7.65-7.41 (m, 5H), 7.28-7.22 (m, 1H), 7.13 (d, J_1 = 7.9 Hz, 2H), 3.96 (s, 3H); **^{13}C NMR ($CDCl_3$)**: δ 159.2, 139.4, 137.5, 131.7, 130.8, 129.4, 128.9, 127.7, 127.31, 127.29, 126.0, 124.3, 123.7, 123.0, 121.8, 119.8, 114.5, 112.6, 109.7, 55.4; **HRMS [M+H]$^+$**: calcd for $C_{22}H_{17}N_2O$ 325.1335, found 325.1335.

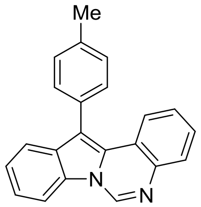

12-(*p*-Tolyl)indolo[1,2-*c*]quinazoline (10b)
Off-white powder, **yield**: 61%, mp: 192-193 °C; **IR (KBr):** 3060, 2931, 2847, 1740, 1560,1450 (cm^{-1}); **^{1}H NMR ($CDCl_3$):** δ 8.92 (s, 1H), 7.85 (d, *J* = 8.4 Hz, 1H), 7.73 (d, *J* = 8.4 Hz, 1H), 7.66 (d, *J* = 8.0 Hz, 1H), 7.53-7.49 (m, 1H), 7.40-7.26 (m, 7H), 7.15-7.09 (m, 1H), 2.40 (s, 3H); **^{13}C NMR ($CDCl_3$):** 140.1, 137.4, 137.3, 131.2, 130.54, 130.50, 129.8, 129.4, 128.8, 128.1, 127.4, 127.1, 124.0, 123.7, 122.8, 121.9, 119.8, 112.5, 109.5, 21.4; **HRMS [M+H]$^+$**: calcd for $C_{22}H_{17}N_2$ 309.1386, found 309.1385.

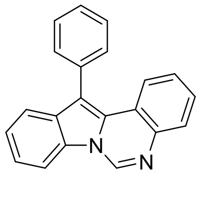

12-Phenylindolo[1,2-*c*]quinazoline (10c)
Yellow powder, **yield**: 75%,mp: 155-156 °C; **IR (KBr):** 3066, 2934, 2853, 1740, 1579,1450 (cm^{-1}); **^{1}H NMR ($CDCl_3$):** δ 8.90 (s, 1H), 7.83 (d, *J* = 7.6 Hz, 1H), 7.67 (t, *J* = 8.4 Hz, 2H), 7.54-7.26 (m, 9H), 7.10 (d, J_1 = 7.6 Hz, J_2 = 0.8 Hz, 1H); **^{13}C NMR ($CDCl_3$):**140.2, 137.2, 134.3, 130.7, 130.4, 129.4, 129.0, 128.9, 128.2, 127.7, 127.5, 127.2, 124.2, 123.7, 122.9, 121.8, 119.7, 112.5, 109.5; **HRMS [M+H]$^+$**: calcd for $C_{21}H_{15}N_2$ 295.1230, found 295.1229.

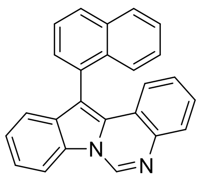

12-(Naphthalen-1-yl)indolo[1,2-*c*]quinazoline (10d)

Pale yellow powder, **yield**: 60%, mp: 192-193 °C; **IR (KBr):** 3042, 2925, 1618, 1469, 1445 (cm^{-1}); **^{1}H NMR ($CDCl_3$):** δ 9.07 (s, 1H), 8.00-7.90 (m, 3H), 7.70 (d, *J* = 7.7 Hz, 1H), 7.59 (s, 1H), 7.58 (d, *J* = 1.3 Hz, 1H), 7.52 (d, *J* = 8.3 Hz, 1H), 7.46-7.36 (m, 2H), 7.34-7.25 (m, 3H), 7.24-7.18 (m, 1H), 7.13 (dd, J_1 = 8.0 Hz, J_2 = 0.9 Hz, 1H), 6.98-6.90 (m, 1H); **^{13}C NMR ($CDCl_3$):** δ 140.2, 137.3, 134.1, 132.7, 131.8, 131.1, 129.6, 129.0, 128.8, 128.7, 128.5, 128.4, 128.0, 127.3, 126.4, 126.20, 126.19, 126.0, 124.2, 122.9, 121.7, 120.2, 109.9, 109.6; **HRMS [M+H]$^+$**: calcd for $C_{25}H_{17}N_2$ 345.1386, found 345.1385.

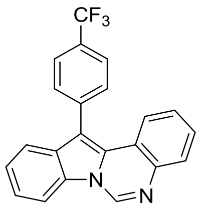

12-(4-(Trifluoromethyl)phenyl)indolo[1,2-*c*]quinazoline (10e)

Yellow powder, **yield**: 69%, mp: 188-189 °C; **IR (KBr):** 2924, 2855, 1616, 1473(cm^{-1}); **^{1}H NMR ($CDCl_3$):** δ 9.00 (s, 1H), 7.93 (d, *J* = 7.8 Hz, 1H), 7.80-7.63 (m, 6H), 7.54-7.50 (m, 1H), 7.47-7.33 (m, 3H), 7.22-7.15 (m, 1H); **^{13}C NMR ($CDCl_3$):** δ 140.3 (q, $J_{C\text{-}F}$ = 1.5 Hz, 1H), 138.5, 137.0, 131.0, 129.9, 129.8 (q, J_{CF} = 32 Hz, 1H), 129.6, 129.3, 128.5, 127.9, 127.4, 126.0 (q, J_{CF} = 4 Hz), 124.3 (q, J_{CF} = 272 Hz), 124.5, 123.5, 123.2, 121.3, 119.3, 110.8, 109.7; **^{19}F NMR ($CDCl_3$):** δ -63.33. **HRMS [M+H]$^+$**: calcd for $C_{22}H_{14}F_3N_2$ 363.1104, found 363.1101.

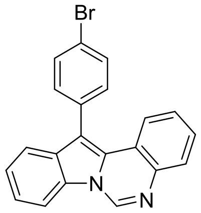

12-(4-Bromophenyl)indolo[1,2-*c*]quinazoline (10f)

beige powder, **yield**: 60%, mp: 207-208 °C; **IR (KBr):** 3042, 2921, 1629, 1457 cm^{-1}; **^{1}H NMR ($CDCl_3$):** δ 8.96 (s, 1H), 7.89 (d, *J* = 7.4 Hz, 1H), 7.66-7.62 (m, 2H), 7.61 (d, *J* = 8.4 Hz, 2H), 7.55-7.46 (m, 1H), 7.44-7.30 (m 5H), 7.21-7.14 (m, 1H); **^{13}C NMR ($CDCl_3$):** δ 140.2, 137.1, 133.4, 132.4, 132.3, 130.1, 129.5, 129.1, 128.3, 127.7, 127.3, 124.3, 123.6,

123.0, 121.8, 121.5, 119.4, 111.0, 109.7; **HRMS [M+H]$^+$**: calcd for $C_{21}H_{14}BrN_2$ 373.0345, found 373.0334.

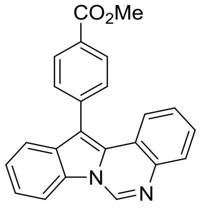

Methyl 4-(indolo[1,2-*c*]quinazolin-12-yl)benzoate (10g)
Pale yellow powder, **yield**: 59%, mp: 170-171 °C; **IR (KBr):** 3404, 3048, 2923, 1637, 1475 (cm^{-1}); **^{1}H NMR (CDCl$_3$):** δ 9.03 (s, 1H), 8.26 (d, *J* = 8.4 Hz, 2H), 8.02-7.99 (m, 1H), 7.80 (td, J_1 = 8.3 Hz, J_2 = 1.1 Hz, 2H), 7.72 (d, *J* = 8.4 Hz, 2H), 7.65-7.61 (m, 1H), 7.54-7.42 (m, 3H), 7.28-7.22 (m, 1H), 4.00 (s, 3H); **^{13}C NMR (CDCl$_3$):** δ 167.02, 140.3, 139.6, 137.1, 130.7, 130.3, 129.9, 129.6, 129.4, 129.3, 128.4, 127.9, 127.3, 124.4, 123.6, 123.1, 121.4, 119.4, 111.4, 109.7, 52.3; **HRMS [M+H]$^+$**: calcd for $C_{23}H_{17}N_2O_2$ 353.1285, found 353.1276.

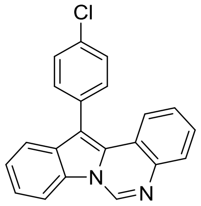

12-(4-Chlorophenyl)indolo[1,2-*c*]quinazoline (10h)
Off-white powder, **yield**: 52%, mp: 236-237 °C; **IR (KBr):** 3048, 2921, 1619, 1474 (cm^{-1}); **^{1}H NMR (CDCl$_3$):** δ 9.03(s, 1H), 7.97 (d, *J* = 7.2 Hz, 1H), 7.77 (t, *J* = 8.2 Hz, 2H), 7.60-7.40 (m, 8H), 7.26-7.22 (m, 1H); **^{13}C NMR (CDCl$_3$):** δ 140.2, 137.1, 133.6, 132.9, 132.0, 130.1, 129.4, 129.3, 129.1, 128.3, 127.7, 127.3, 124.3, 123.5, 123.0, 121.5, 119.4, 111.1, 109.7; **HRMS [M+H]$^+$**: calcd for $C_{21}H_{14}ClN_2$ 329.0840, found 329.0839.

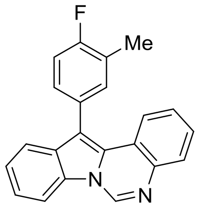

12-(4-Fluoro-3-methylphenyl)indolo[1,2-*c*]quinazoline (10i)
Pale yellow powder, **yield**: 65%, mp: 165-166 °C; **IR (KBr):** 3034, 2943, 1625, 1445 (cm^{-1}); **^{1}H NMR (CDCl$_3$):** δ 9.02 (s, 1H), 7.95 (d, *J* = 7.4 Hz, 1H), 7.75 (t, *J* = 8.4 Hz, 2H), 7.59-7.54 (m, 1H), 7.48-7.30 (m, 5H), 7.27-7.14 (m, 2H), 2.37 (s, 3H); **^{13}C NMR (CDCl$_3$):** δ 161.0 (d, J_{CF} = 244 Hz), 140.2, 137.2, 133.6 (d, J_{CF} =5 Hz),130.4, 129.9 (d, J_{CF} = 4 Hz),

129.6, 129.4, 128.9, 128.2, 127.6, 127.2, 125.6 (d, J_{CF} = 17 Hz), 124.1, 123.6, 122.9, 121.8, 119.6, 115.7 (d, J_{CF} = 23 Hz), 111.6, 109.7, 14.7; **^{19}F NMR ($CDCl_3$):** δ -118.7; **HRMS [M+H]$^+$**: calcd for $C_{22}H_{16}FN_2$, 327.1292, found 327.1297.

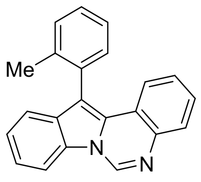

12-(*o*-Tolyl)indolo[1,2-*c*]quinazoline (10j)
Beige powder, **yield**: 56%, mp: 195-196 °C; **IR (KBr):** 3055, 3014, 1617, 1604, 1469, 1449 (cm^{-1}); **^{1}H NMR (CDCl$_3$):** δ 9.00 (s, 1H), 7.93 (d, *J* = 8.0 Hz, 1H), 7.70 (d, *J* = 8.0 Hz, 1H), 7.41-7.25 (m, 9H), 7.16-7.10 (m, 1H), 2.02 (s, 3H); **^{13}C NMR (CDCl$_3$):** δ 140.0, 138.3, 137.3, 135.4, 133.5, 131.3, 130.5, 130.2, 129.1, 128.8, 128.2, 128.0, 127.5, 126.4, 124.1, 123.5, 122.8, 122.1, 119.9, 111.4, 109.6, 20.0; **HRMS [M+H]$^+$**: calcd for $C_{22}H_{17}N_2$ 309.1386, found 309.1384.

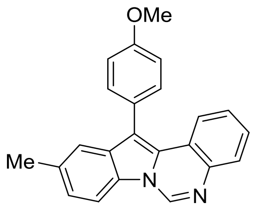

12-(4-Methoxyphenyl)-10-methylindolo[1,2-*c*]quinazoline (10k)
Pale yellow powder, **yield**: 63%**,** mp: 177-178 °C**; IR (KBr):** 2930, 2858, 2828, 1614, 1556, 1455 (cm^{-1}); **^{1}H NMR (CDCl$_3$):** δ 9.01 (s, 1H), 8.86 (d, *J* = 8.4 Hz, 1H), 7.84-7.75 (m, 2H), 7.52 (d, *J* = 8.6 Hz, 2H), 7.49-7.41 (m, 1H), 7.39 (s, 1H), 7.30-7.20 (m, 2H), 7.14 (d, *J* = 8.6 Hz, 2H), 3.96 (s, 3H), 2.51 (s, 3H); **^{13}C NMR (CDCl$_3$):** δ 159.2, 140.1, 137.3, 133.9, 131.8, 130.9, 128.7, 128.0, 127.7, 127.6, 127.1, 126.5, 124.4, 123.6, 122.0, 119.3, 114.5, 111.8, 109.2, 55.4, 21.7; **HRMS [M+H]$^+$**: calcd for $C_{23}H_{19}N_2O$ 339.1492, found 339.1493.

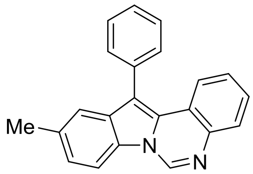

10-Methyl-12-phenylindolo[1,2-*c*]quinazoline (10l)
Off-white powder, **yield**: 72%, mp: 173-174 °C; **IR (KBr):** 3056, 2921, 2853, 1740, 1599,1450 (cm^{-1}); **^{1}H NMR (CDCl$_3$):** δ 8.94 (s, 1H), 7.79 (d, *J* = 8.4 Hz, 1H), 7.68 (d, *J* = 8.2 Hz, 2H), 7.55-7.46 (m, 4H), 7.45-7.34 (m, 2H), 7.30 (s, 1H), 7.21-7.09 (m, 2H), 2.41 (s, 3H); **^{13}C NMR (CDCl$_3$):** δ 140.2, 137.3, 134.5, 134.0, 130.71, 130.68, 129.0, 128.8, 128.1,

127.8, 127.7, 127.6, 127.1, 124.5, 123.7, 121.9, 119.2, 112.1, 109.2, 21.7; **HRMS [M+H]$^+$**: calcd for $C_{22}H_{17}N_2$ 309.1386, found 309.1392.

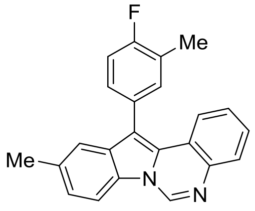

12-(4-Fluoro-3-methylphenyl)-10-methylindolo[1,2-*c*]quinazoline (10m)
Pale yellow powder, **yield**: 76%, mp: 170-171 °C; **IR (KBr):** 3058, 2922, 1639, 1445 (cm^{-1}); **^{1}H NMR (CDCl$_3$):** δ 9.01 (s, 1H), 7.87 (d, J = 8.4 Hz, 1H), 7.79 (d, J = 8.0 Hz, 1H), 7.75 (d, J = 8.0 Hz, 1H), 7.47 (t, J = 7.6 Hz, 1H), 7.44-7.33 (m, 3H), 7.32-7.18 (m, 3H), 2.52 (s, 3H), 2.42 (s, 3H); **^{13}C NMR (CDCl$_3$):** δ 161.0 (d, J_{CF} = 245 Hz), 140.1, 137.3, 134.0, 133.6 (d, J_{CF} = 5 Hz), 130.7, 130.0 (d, J_{CF} = 4 Hz), 129.64, 129.56, 128.8, 128.2, 127.7, 121.1, 125.6 (d, J_{CF} = 17 Hz), 124.5, 123.5, 121.8, 119.1, 115.7 (d, J_{CF} = 23 Hz), 111.2, 109.3, 21.8, 14.8 (d, J_{CF} = 3 Hz); **^{19}F NMR (CDCl$_3$):** δ -118.7; **HRMS [M+H]$^+$**: calcd for $C_{23}H_{18}FN_2$ 341.1449, found 341.1448.

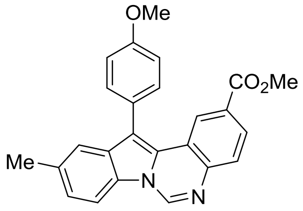

Methyl 12-(4-methoxyphenyl)-10-methylindolo[1,2-c]quinazoline-2-carboxylate (10n)
Beige powder, **yield**: 60%, mp: 187.0-188 °C; **IR (KBr):** 3400, 2935, 1634, 1450 (cm^{-1}); **^{1}H NMR (CDCl$_3$):** δ 9.04 (s, 1H), 8.55 (d, J = 1.7 Hz, 1H), 8.07 (dd, J_1 = 8.4, J_2 = 1.7 Hz, 1H), 7.88 (d, J = 8.4 Hz, 1H), 7.78 (d, J = 8.4 Hz, 2H), 7.54 (dd, J_1 = 8.7, J_2 = 1.9 Hz, 2H), 7.44 (s, 1H), 7.31 (dd, J_1 = 8.4, J_2 = 0.9 Hz, 1H), 7.17 (d, J = 8.7 Hz 1H), 3.97 (s, 3H), 3.85 (s, 3H), 2.52 (s, 3H); **^{13}C NMR (CDCl$_3$):** δ 166.3, 159.4, 143.3, 138.9, 134.3, 131.5, 130.8, 129.2, 128.2, 128.0, 127.8, 126.9, 125.7, 125.4, 124.9, 121.8, 119.5, 114.6, 113.0, 109.3, 55.4, 52.1, 21.7; **HRMS [M+H]$^+$**: calcd for $C_{25}H_{21}N_2O_3$ 397.1547, found 397.1556.

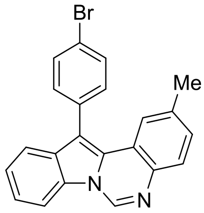

12-(4-Bromophenyl)-2-methylindolo[1,2-*c*]quinazoline (10o)
Light brown powder, **yield**: 58%, mp: 221-222 °C; **IR (KBr):** 3056, 2922, 1620, 1559, 1469, 1221 (cm^{-1}); **^{1}H NMR ($CDCl_3$):** δ 8.95 (s, 1H), 7.91 (d, *J* = 7.3 Hz, 1H), 7.64-7.58 (m, 3H), 7.58-7.49 (m, 2H), 7.46-7.32 (m, 4H), 7.24 (dd, J_1 = 8.0 Hz, J_2 = 1.6 Hz, 1H), 2.23 (s, 3H); **^{13}C NMR ($CDCl_3$):** 138.1, 137.4, 136.5, 133.5, 132.3, 132.1, 130.4, 130.0, 129.5, 128.2, 127.7, 124.2, 123.6, 123.0, 121.7, 121.3, 119.4, 110.8, 109.7, 21.7; **HRMS $[M+H]^+$**: calcd for $C_{22}H_{16}BrN_2$ 387.0491, found 387.0492.

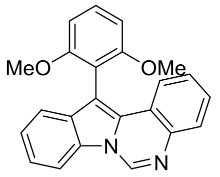

12-(2,6-Dimethoxyphenyl)indolo[1,2-*c*]quinazoline (10p)
Off-white powder, **yield**: 12%, mp: 210-211 °C; **IR (KBr):** 3022**,** 2925, 1618, 1470(cm^{-1}); **^{1}H NMR ($CDCl_3$):** δ 9.07 (s, 1H), 7.98 (d, *J* = 7.3 Hz, 1H), 7.76 (d, *J* = 8.1 Hz, 1H), 7.59 (dd, J_1 = 8.1 Hz, J_2 = 0.9 Hz, 1H), 7.50-7.34 (m, 5H), 7.26-7.21 (m, 1H), 6.78 (d, *J* = 8.4 Hz, 2H), 3.66 (s, 6H); **^{13}C NMR ($CDCl_3$):** δ 159.1, 140.0, 137.4, 130.2, 129.71, 129.70, 128.67, 128.4, 127.5, 127.0, 123.9, 123.7, 122.7, 122.2, 120.5, 111.1, 109.6, 104.3, 103.7, 55.9; **HRMS $[M+H]^+$**: calcd for $C_{23}H_{19}N_2O_2$ 355.1441, found 355.1436.

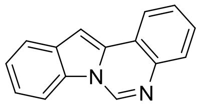

Indolo[1,2-*c*]quinazoline (13a)
White powder, **yield**: 86%, mp: 205-206 °C; **IR (KBr):** 2922, 1593, 1458 (cm^{-1}); **^{1}H NMR ($CDCl_3$):** δ 9.0 (s, 1H), 8.05 (d, *J* = 7.8 Hz, 1H), 7.95 (d, *J* = 7.8 Hz, 1H), 7.86-7.78 (m, 2H), 7.60-7.38 (m, 4H), 7.12 (s, 1H); **^{13}C NMR ($CDCl_3$):** δ 139.4, 137.0, 132.9, 130.3, 129.7, 129.1, 128.1, 127.7, 124.1, 123.1, 122.2, 121.3, 120.9, 109.9, 94.7 **HRMS $[M+H]^+$**: calcd for $C_{15}H_{11}N_2$ 219.0917, found 219.0918.

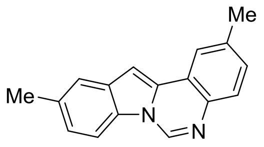

2,10-Dimethylindolo[1,2-*c*]quinazoline (13b)
Light brown powder, **yield**: 87%, mp: 202.0-203.3 °C; **IR (KBr):** 2922, 1540, 1370 (cm^{-1}); **^{1}H NMR (CDCl$_3$):** δ 8.86 (s, 1H), 7.77-7.71 (m, 2H), 7.61 (d, *J* = 8.2 Hz, 1H), 7.51 (s, 1H), 7.27 (dd, J_1 = 8.2 Hz, J_2 = 1.5 Hz, 1H), 7.14 (dd, J_1 = 8.4 Hz, J_2 = 1.1 Hz, 1H), 6.93 (s, 1H), 2.47 (s, 3H), 2.45 (s, 3H); **^{13}C NMR (CDCl$_3$):** δ 137.6, 137.4, 136.5, 133.7, 133.1, 130.3, 130.0, 128.7, 127.9, 123.7, 123.0, 121.1, 120.5, 109.5, 93.9, 21.8, 21.6; **HRMS [M+H]$^+$**: calcd for $C_{17}H_{15}N_2$ 247.1230, found 247.1230.

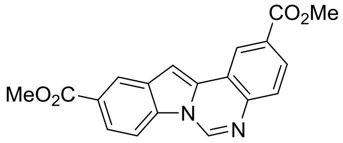

Dimethyl indolo[1,2-*c*]quinazoline-2,10-dicarboxylate (13c)
Yellow powder, **yield**: 81%, mp: 207-208 °C; **IR (KBr):** 2916, 1701, 1386 (cm^{-1}); **^{1}H NMR (CDCl$_3$):** δ 9.01 (s, 1H), 8.69 (d, *J* = 1.8 Hz, 1H), 8.53 (d, *J* = 1.0 Hz, 1H), 8.14 (dd, J_1 = 8.4 Hz, J_2 = 1.8 Hz, 1H), 8.08 (dd, J_1 = 8.7 Hz, J_2 = 1.5 Hz, 1H), 7.93 (d, *J* = 8.7 Hz, 1H), 7.78 (d, *J* = 8.5 Hz, 1H), 7.26 (s, 1H), 3.95 (s, 3H), 3.93 (s, 3H); **^{13}C NMR (CDCl$_3$):** δ 167.3, 166.3, 142.6, 138.4, 133.5, 132.6, 130.2, 129.3, 129.28, 128.5, 126.4, 125.3, 123.9, 123.8, 121.0, 109.8, 96.7, 52.5, 52.2; **HRMS [M+H]$^+$**: calcd for $C_{19}H_{15}N_2O_4$ 335.1026, found 335.1022.

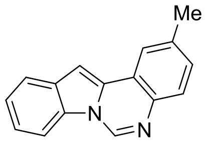

2-Methylindolo[1,2-*c*]quinazoline (13d)
Off-white powder, **yield**: 95%, mp: 145-146 °C; **IR (KBr):** 2916, 2857, 1617, 1488, 1376 (cm^{-1}); **^{1}H NMR (CDCl$_3$):** δ 8.89 (s, 1H), 7.86 (d, *J* = 8.0 Hz, 1H), 7.78-7.72 (m, 2H), 7.61 (d, *J* = 8.0 Hz, 1H), 7.39-7.26 (m, 3H), 7.01 (s, 1H), 2.45 (s, 3H); **^{13}C NMR (CDCl$_3$):** δ 137.8, 137.4, 136.4, 133.0, 130.4, 130.3, 129.7, 127.9, 123.9, 123.0, 122.1, 121.1, 120.9, 109.8, 94.3, 21.62; **HRMS [M+H]$^+$**: calcd for $C_{16}H_{13}N_2$ 233.1073, found 233.1073.

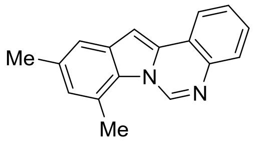

8,10-Dimethylindolo[1,2-*c*]quinazoline (13e)
Pale yellow powder, **yield**: 73%, mp: 196-197 °C; **IR (KBr):** 2926, 1460,1383 (cm^{-1}); **^{1}H NMR ($CDCl_3$):** δ 9.22 (s, 1H), 7.94 (dd, J_1 = 7.7 Hz, J_2 = 1.1 Hz, 1H), 7.68 (d, J = 8.1 Hz, 1H), 7.48-7.34 (m, 3H), 6.99 (s, 1H), 6.91 (s, 1H), 2.77 (s, 3H), 2.42 (s, 3H); **^{13}C NMR ($CDCl_3$):** δ 139.3, 138.8, 133.4, 133.2, 130.6, 128.9, 128.0, 127.7, 127.4, 127.0, 122.9, 122.2, 121.5, 118.3, 95.1, 21.4, 21.3; **HRMS [M+H]$^+$**: calcd for $C_{17}H_{15}N_2$ 247.1232, found 247.1230.

References

[1] (a) Cacchi, S.; Fabrizi, G.; Pace, P. *J. Org. Chem.* **1998**, *63*, 1001-1011; (b) Arcadi, A.; Cacchi, S.; Fabrizi, G.; Ghirga, F.; Goggiamani, A.; Iazzetti, A.; Marinelli, F. *Synthesis* **2018**, 50, 1133-1140.

[2] Arcadi, A.; Cacchi, S.; Fabrizi, G.; Ghirga, F.; Marinelli, F.; Parisi L. M. *Heterocycles.* **2004**, *64,* 475.

[3] Cacchi, S.; Fabrizi, G.; Pace, P. Marinelli, F. *Synlett* **1999**, 620.

[4] Kumar, K. S; Ramulu, M. S.; Rajesham, B.; Kumar, N. P.; Voorab, V.; Kanchab, R. K. *Org. Biomol. Chem*., **2017**,*15*, 4468.

[5] Nitin T. Patil, Rahul D. Kavthe, Vivek S. Raut, Vaddu V. N. Reddy *J. Org. Chem.* **2009**, 6315.

NMR Spectra

10a

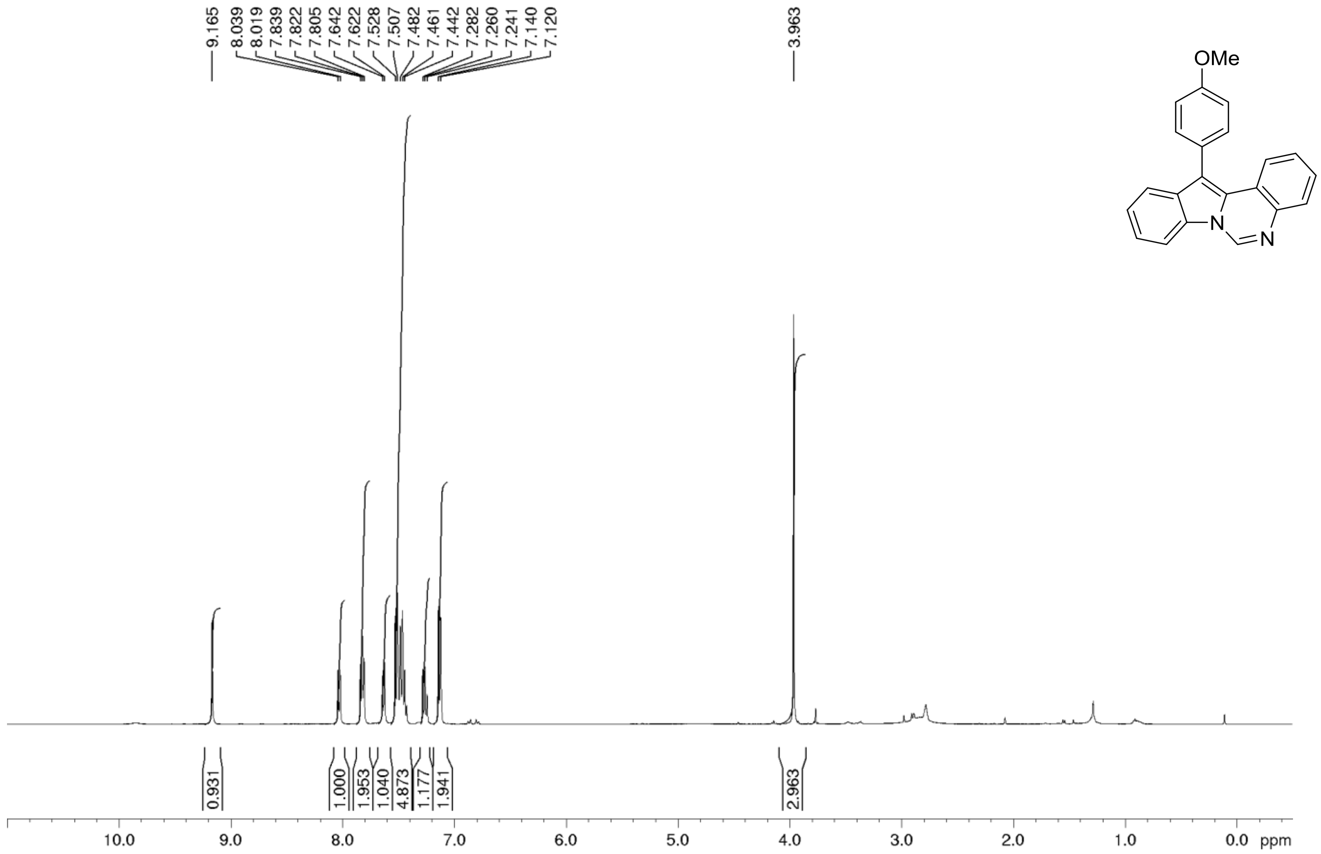

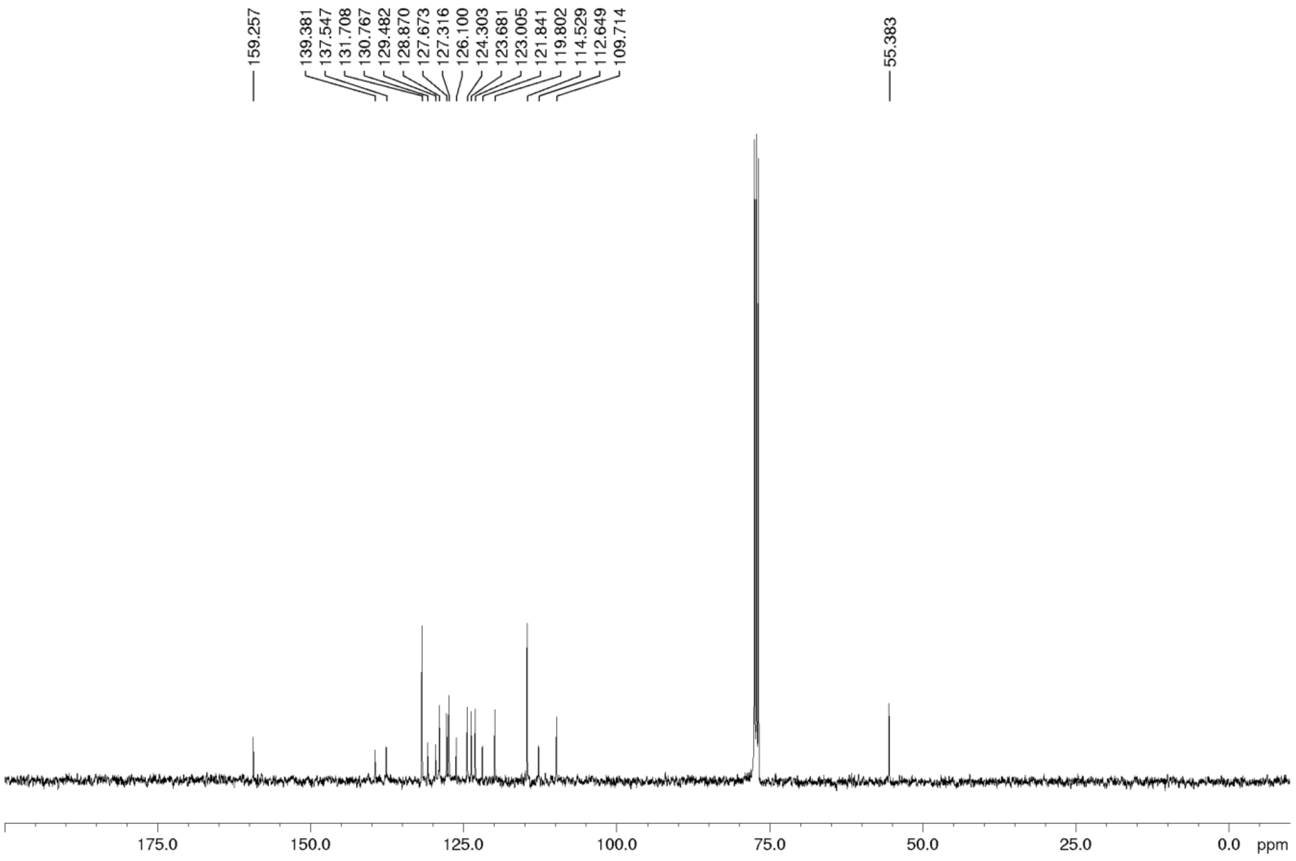

10b

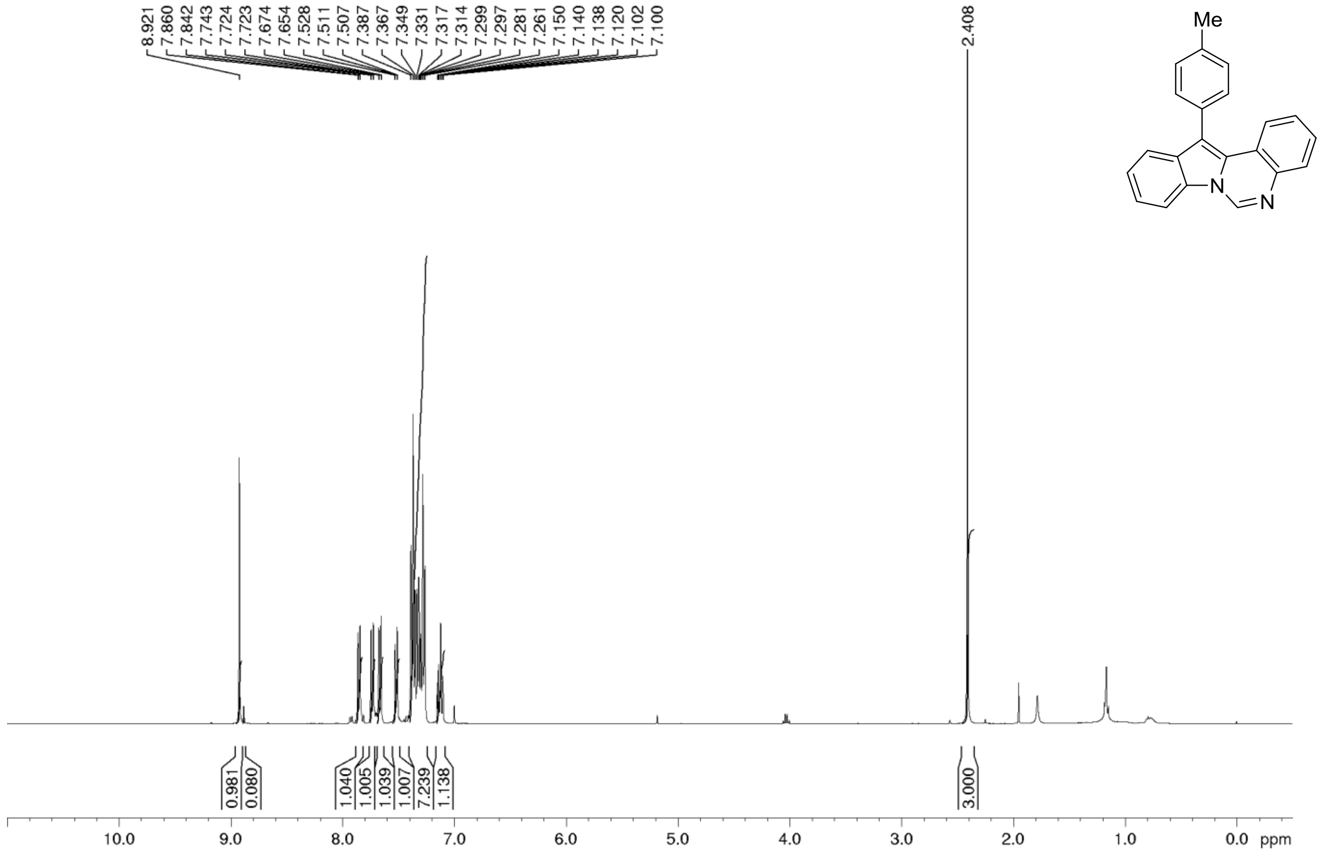
8.921
7.860
7.842
7.743
7.724
7.723
7.674
7.654
7.528
7.511
7.507
7.387
7.367
7.349
7.331
7.317
7.314
7.299
7.297
7.281
7.261
7.150
7.140
7.138
7.120
7.102
7.100
2.408
Me
N
N
0.981
0.080
1.040
1.005
1.039
1.007
7.239
1.138
3.000
10.0
9.0
8.0
7.0
6.0
5.0
4.0
3.0
2.0
1.0
0.0 ppm

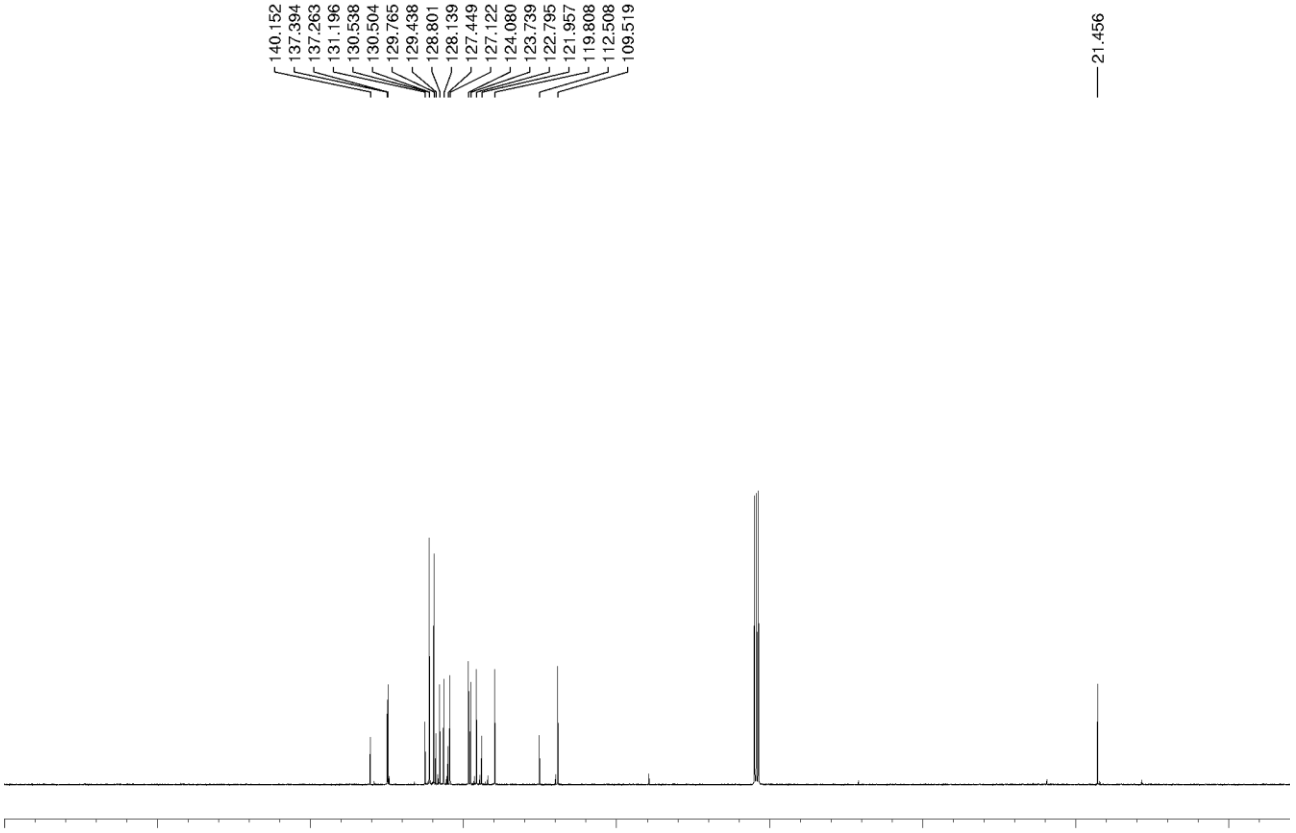
140.152
137.394
137.263
131.196
130.538
130.504
129.765
129.438
128.801
128.139
127.449
127.122
124.080
123.739
122.795
121.957
119.808
112.508
109.519
21.456
175.0
150.0
125.0
100.0
75.0
50.0
25.0
0.0 ppm

10c

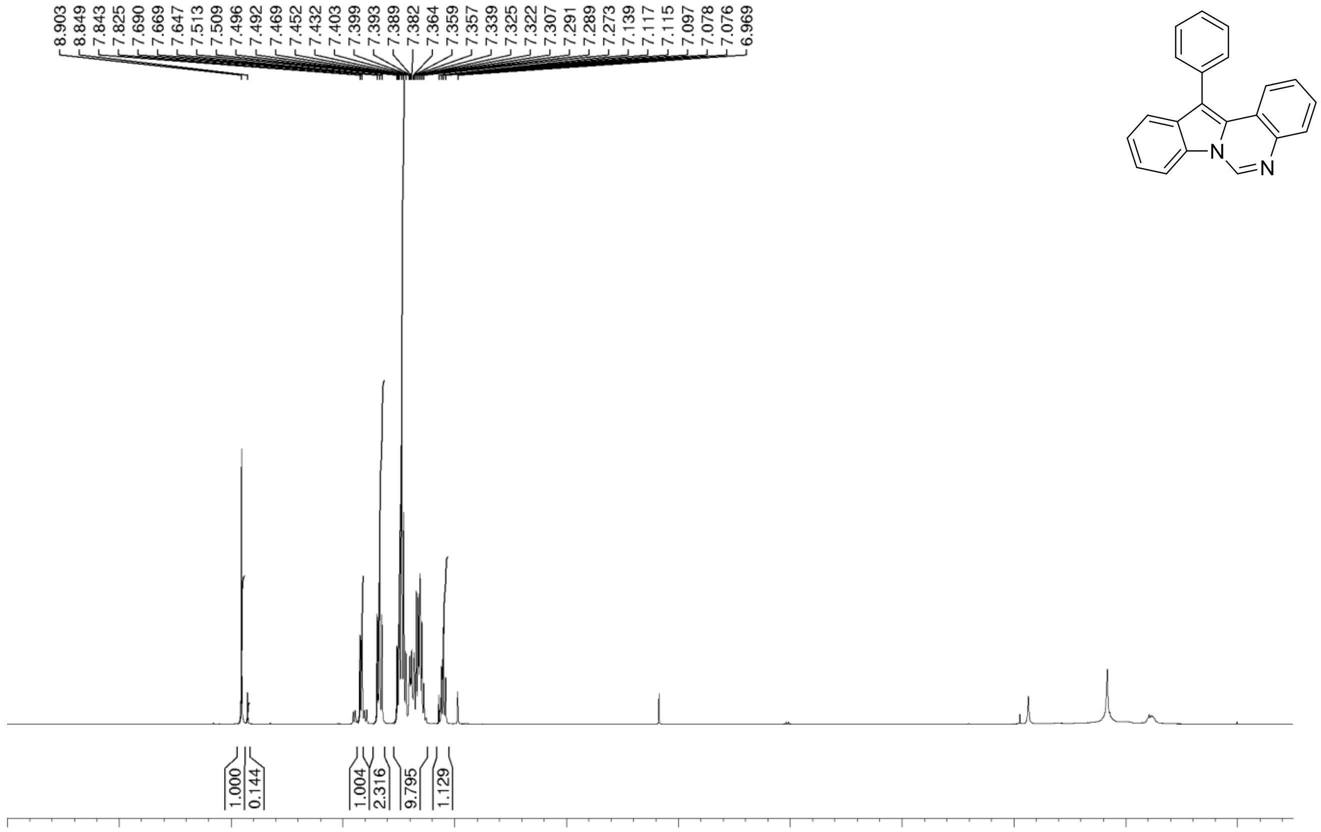
8.903
8.849
7.843
7.825
7.690
7.669
7.647
7.513
7.509
7.496
7.492
7.469
7.452
7.432
7.403
7.399
7.393
7.389
7.382
7.364
7.359
7.357
7.339
7.325
7.322
7.307
7.291
7.289
7.273
7.139
7.117
7.115
7.097
7.076
6.969
N
1.000
0.144
1.004
2.316
9.795
1.129
10.0
9.0
8.0
7.0
6.0
5.0
4.0
3.0
2.0
1.0
0.0 ppm

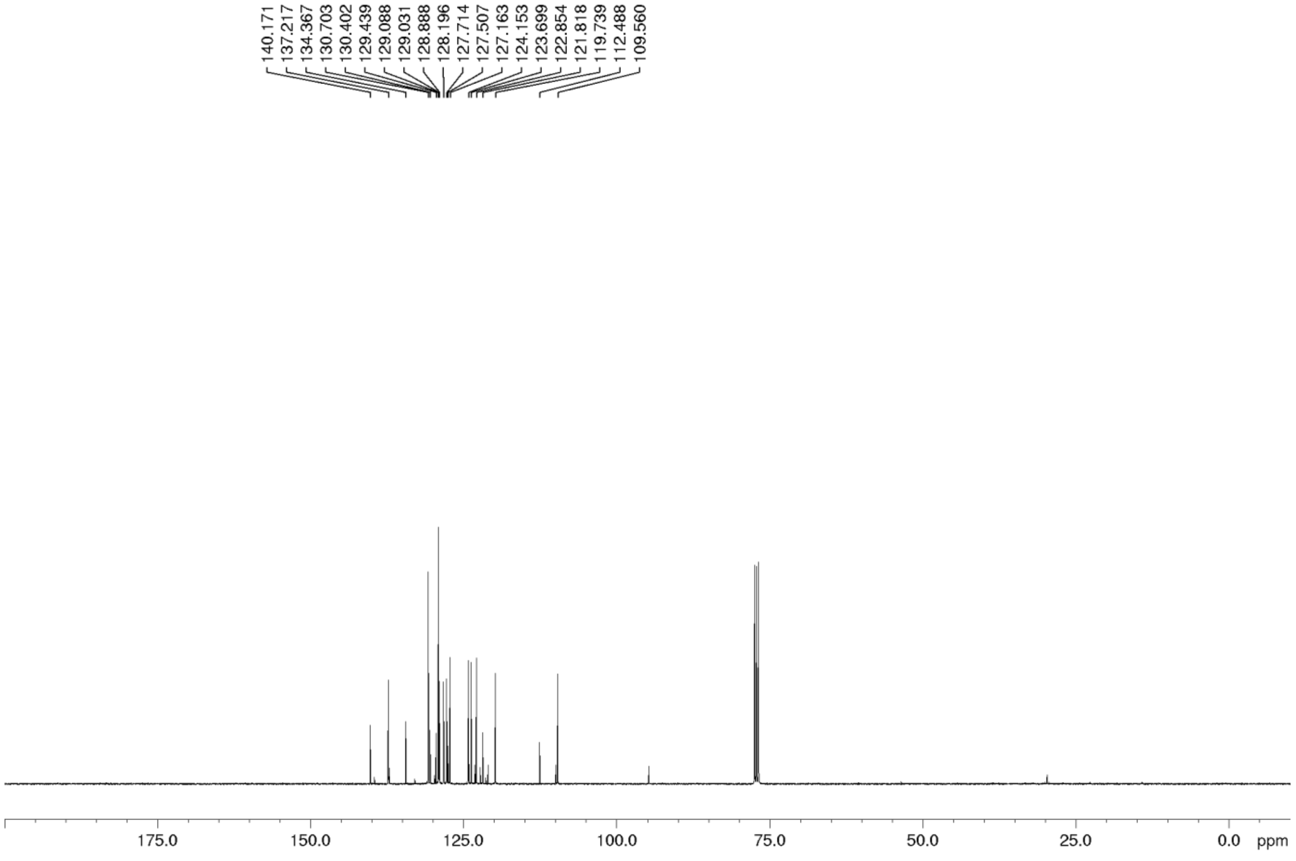
140.171
137.217
134.367
130.703
130.402
129.439
129.088
129.031
128.888
128.196
127.714
127.507
127.163
124.153
123.699
122.854
121.818
119.739
112.488
109.560
175.0
150.0
125.0
100.0
75.0
50.0
25.0
0.0 ppm

10d

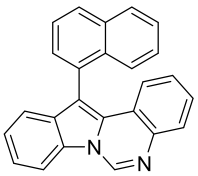

N
N

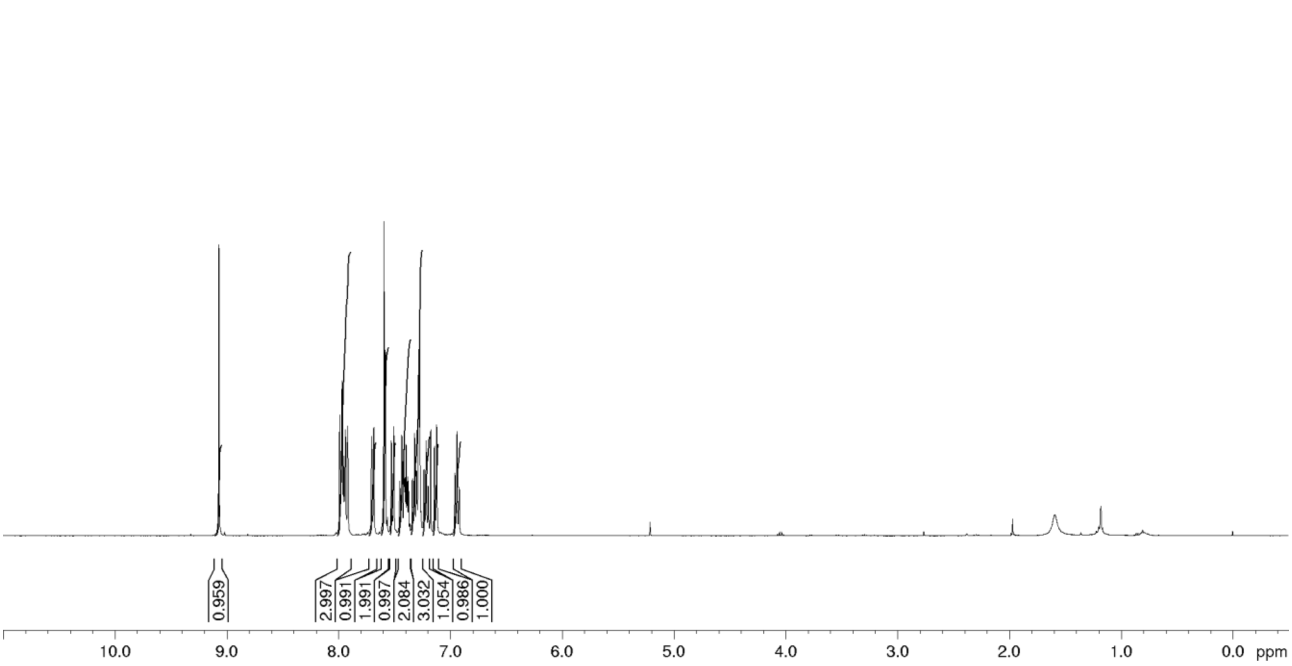

7.950
7.937
7.916
7.702
7.683
7.592
7.582
7.579
7.526
7.505
7.452
7.449
7.432
7.414
7.406
7.401
7.393
7.385
7.381
7.373
7.339
7.335
7.318
7.301
7.297
7.283
7.280
7.272
7.235
7.232
7.215
7.197
7.194
7.174
7.141
7.139
7.121
7.119
6.959
6.956
6.938
6.920
6.918
0.959
2.997
0.991
1.991
0.997
2.084
3.032
1.054
0.986
1.000
10.0
9.0
8.0
7.0
6.0
5.0
4.0
3.0
2.0
1.0
0.0 ppm

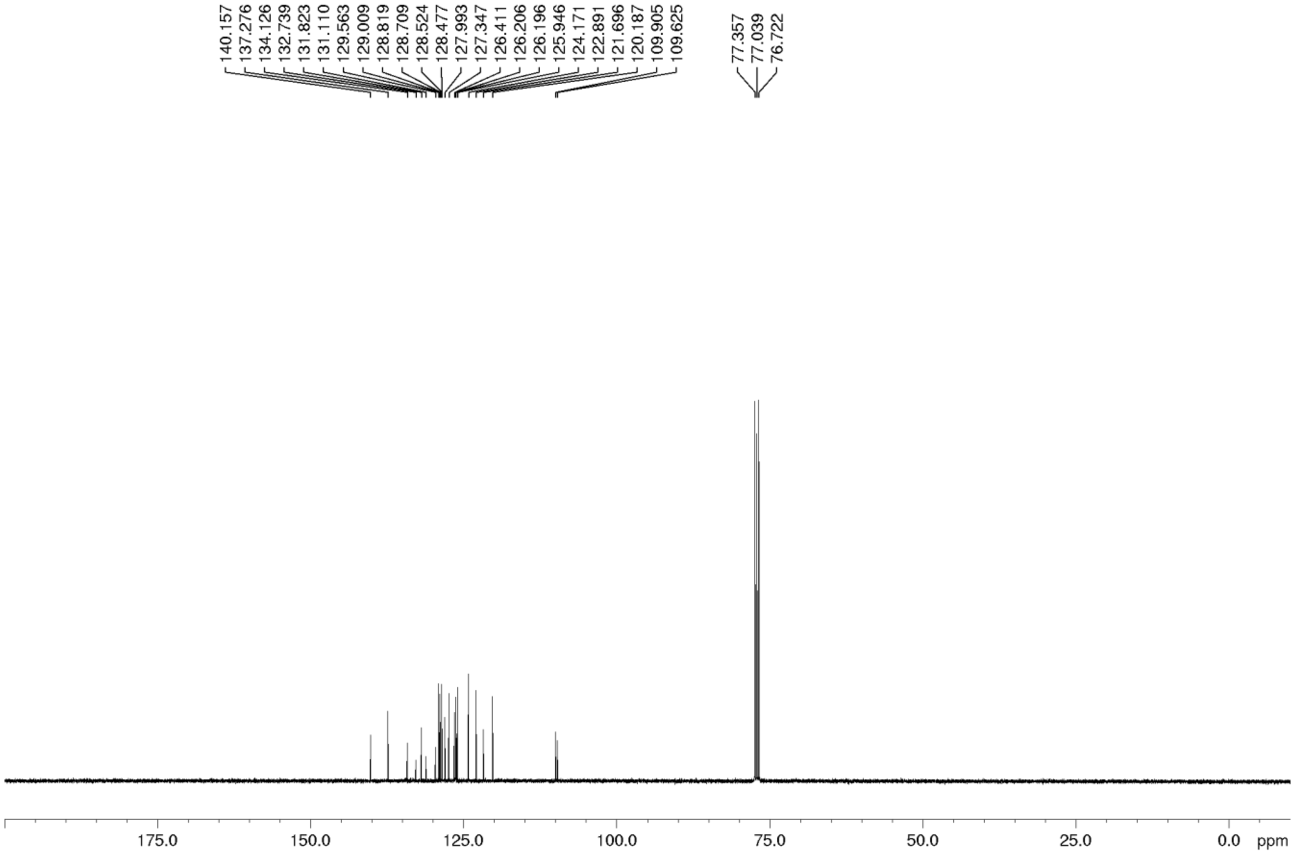

140.157
137.276
134.126
132.739
131.823
131.110
129.563
129.009
128.819
128.709
128.524
128.477
127.993
127.347
126.411
126.206
126.196
125.946
124.171
122.891
121.696
120.187
109.905
109.625
77.357
77.039
76.722
175.0
150.0
125.0
100.0
75.0
50.0
25.0
0.0 ppm

10e

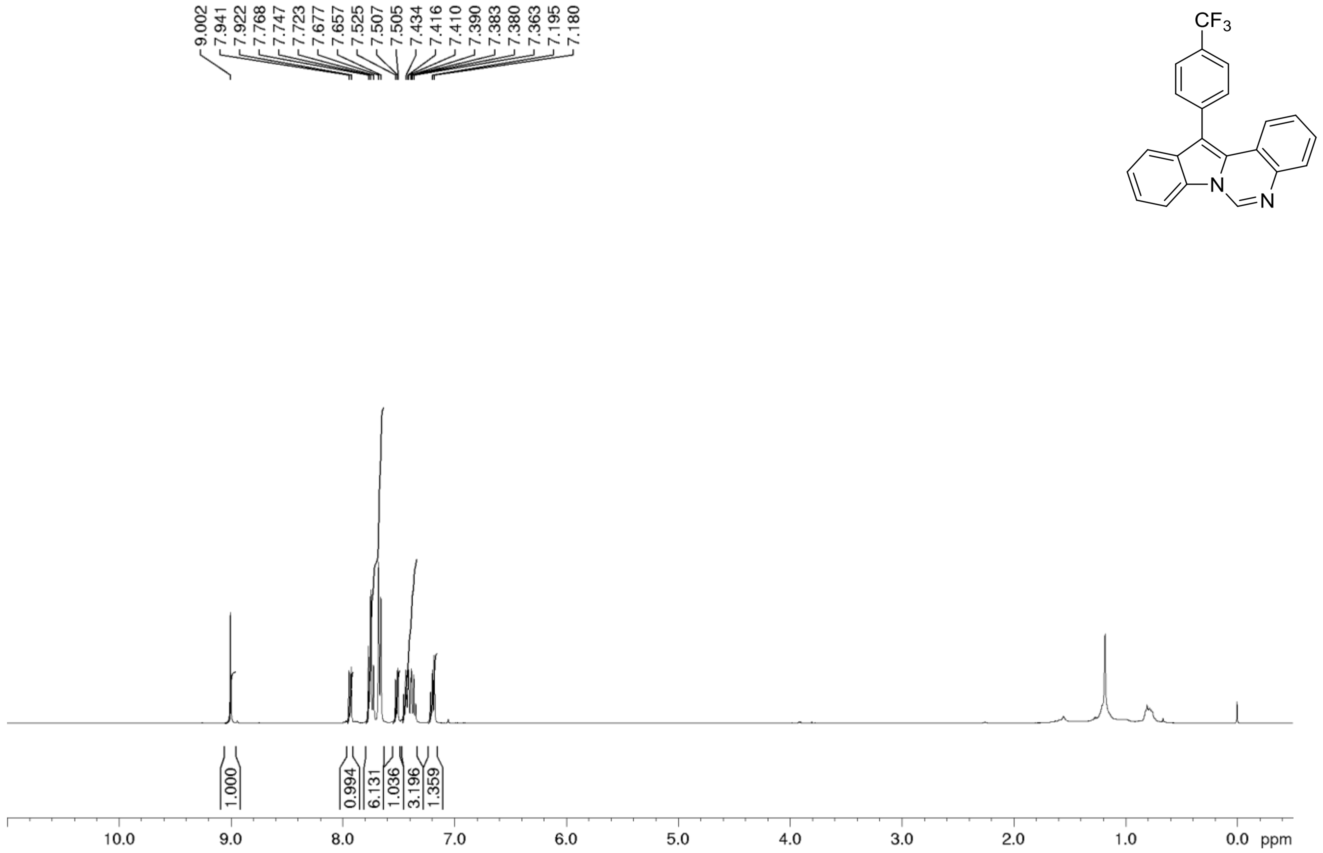
CF3
N
N
9.002
7.941
7.922
7.768
7.747
7.723
7.677
7.657
7.525
7.507
7.505
7.434
7.416
7.410
7.390
7.383
7.380
7.363
7.195
7.180
1.000
0.994
6.131
1.036
3.196
1.359
10.0
9.0
8.0
7.0
6.0
5.0
4.0
3.0
2.0
1.0
0.0 ppm

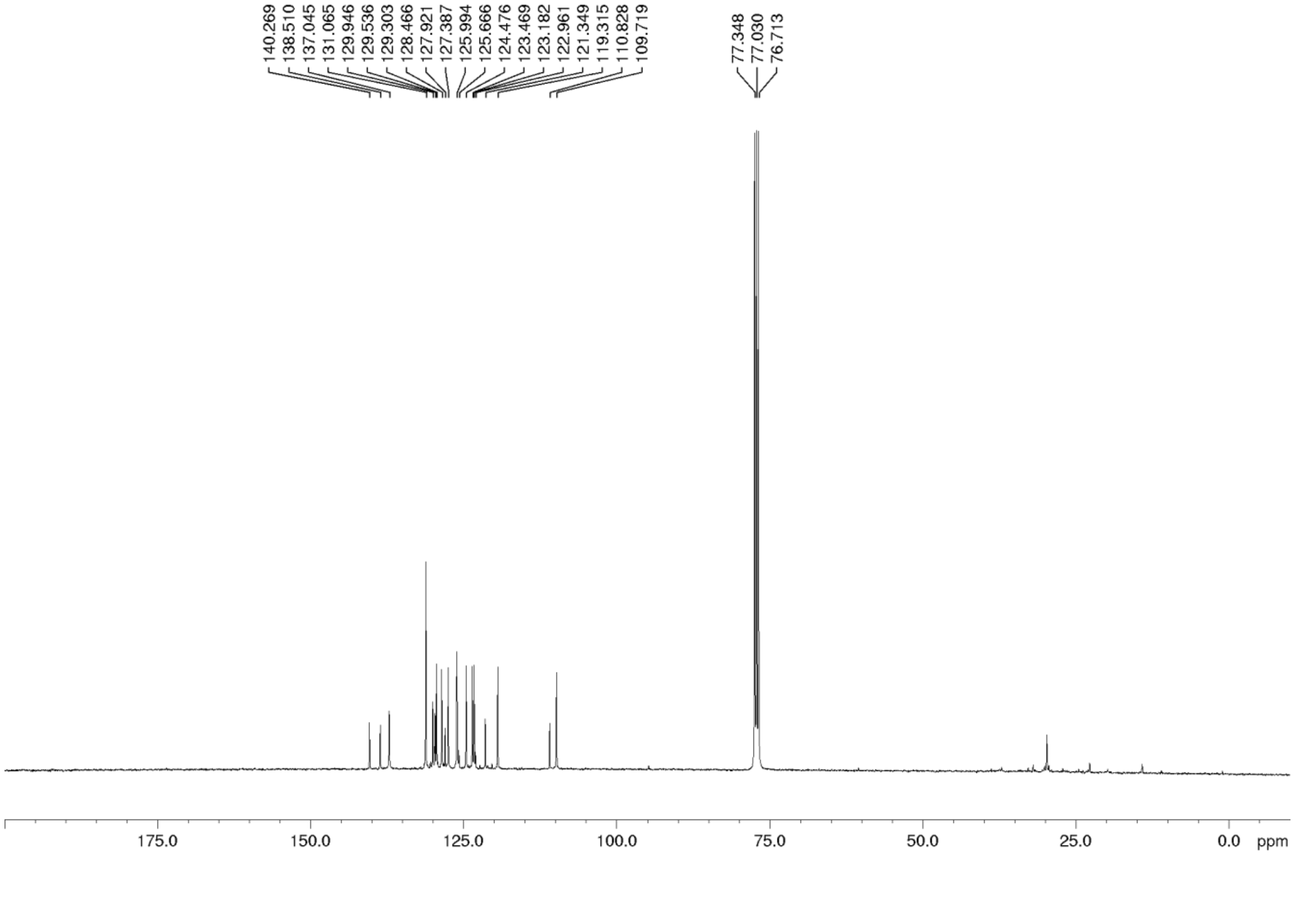
140.269
138.510
137.045
131.065
129.946
129.536
129.303
128.466
127.921
127.387
125.994
125.666
124.476
123.469
123.182
122.961
121.349
119.315
110.828
109.719
77.348
77.030
76.713
175.0
150.0
125.0
100.0
75.0
50.0
25.0
0.0 ppm

10e

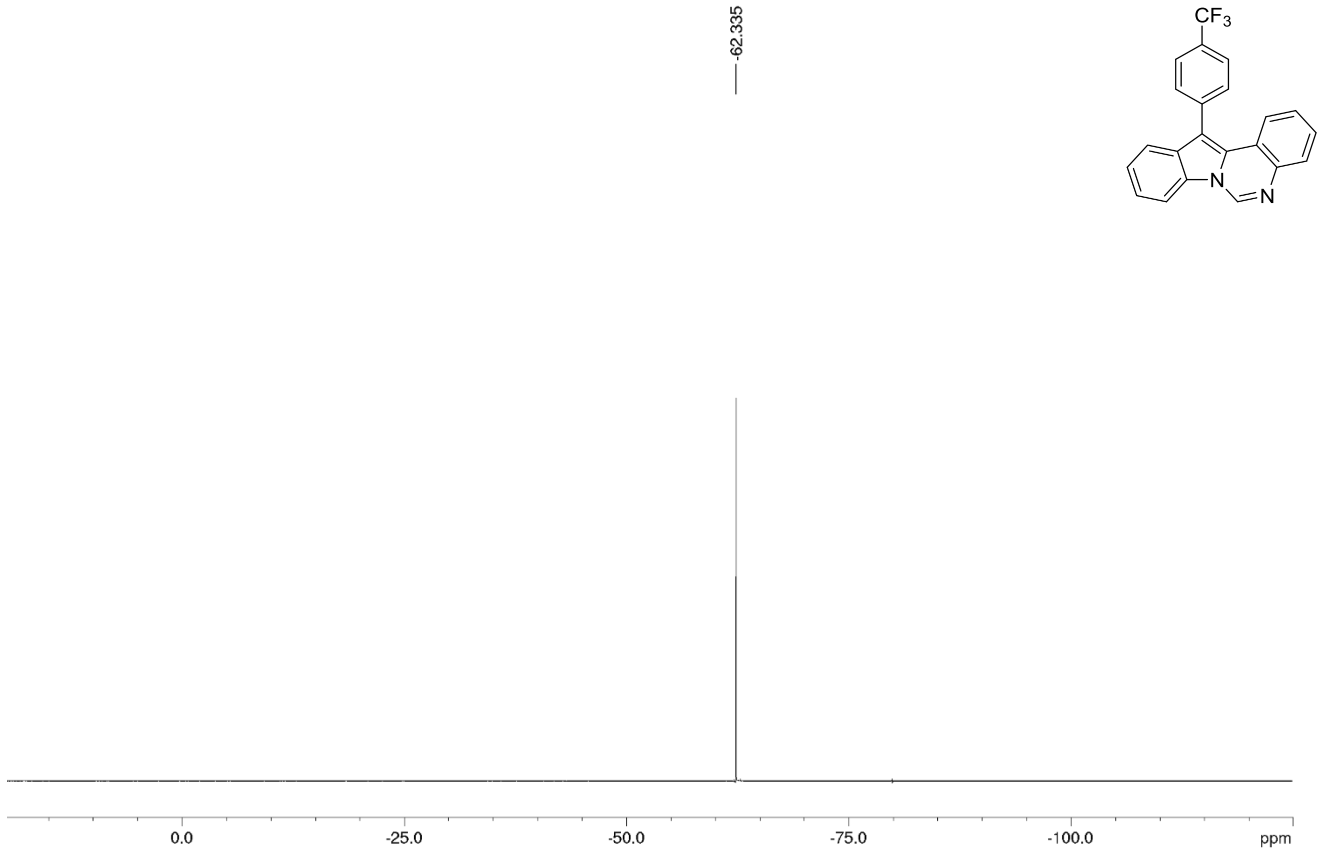

10f

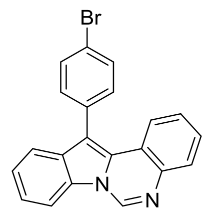
Br
N
N

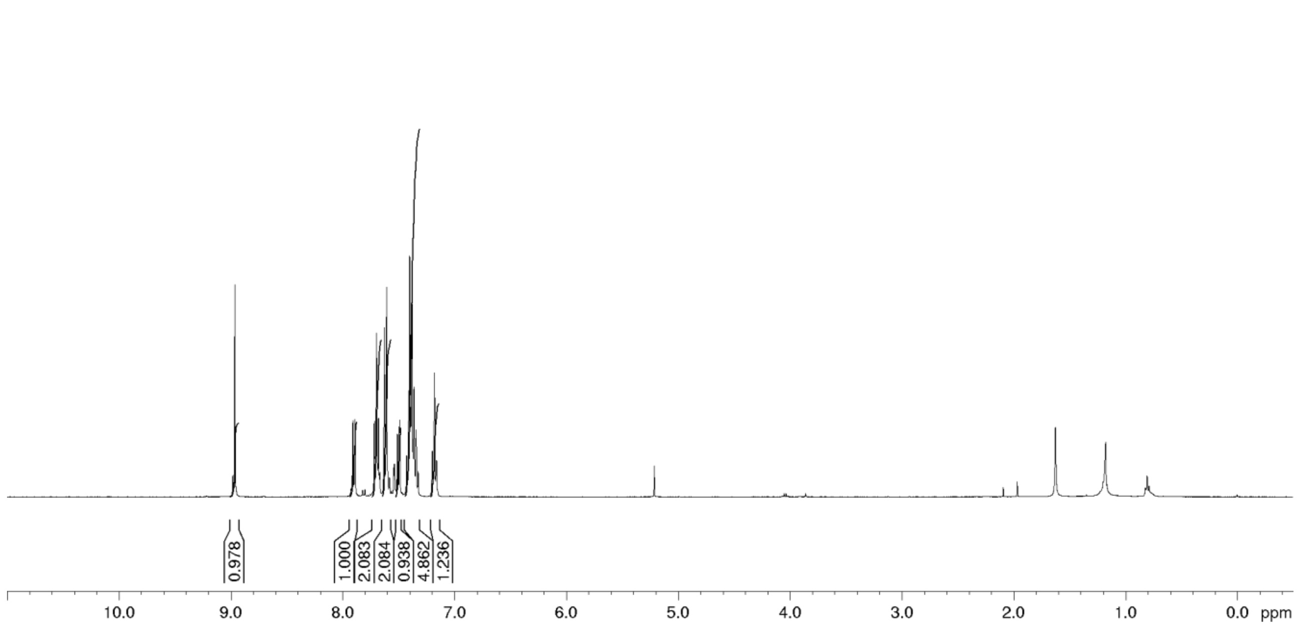
8.962
7.907
7.888
7.715
7.696
7.679
7.677
7.626
7.605
7.540
7.533
7.509
7.491
7.488
7.428
7.425
7.405
7.399
7.390
7.383
7.378
7.362
7.359
7.342
7.340
7.199
7.196
7.176
7.160
7.158
0.978
1.000
2.083
2.084
0.938
4.862
1.236
10.0
9.0
8.0
7.0
6.0
5.0
4.0
3.0
2.0
1.0
0.0 ppm

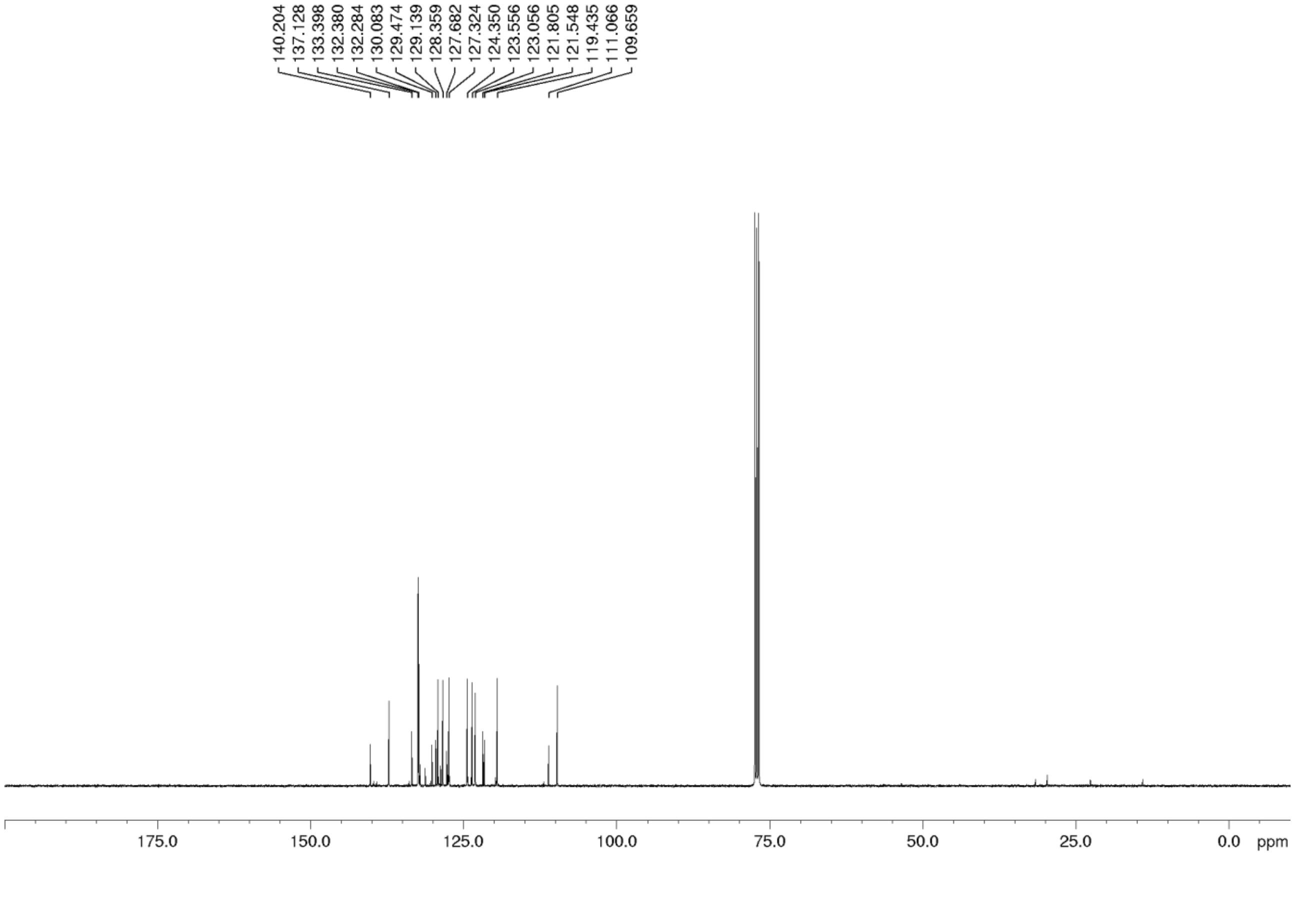
140.204
137.128
133.398
132.380
132.284
130.083
129.474
129.139
128.359
127.682
127.324
124.350
123.556
123.056
121.805
121.548
119.435
111.066
109.659
175.0
150.0
125.0
100.0
75.0
50.0
25.0
0.0 ppm

10g

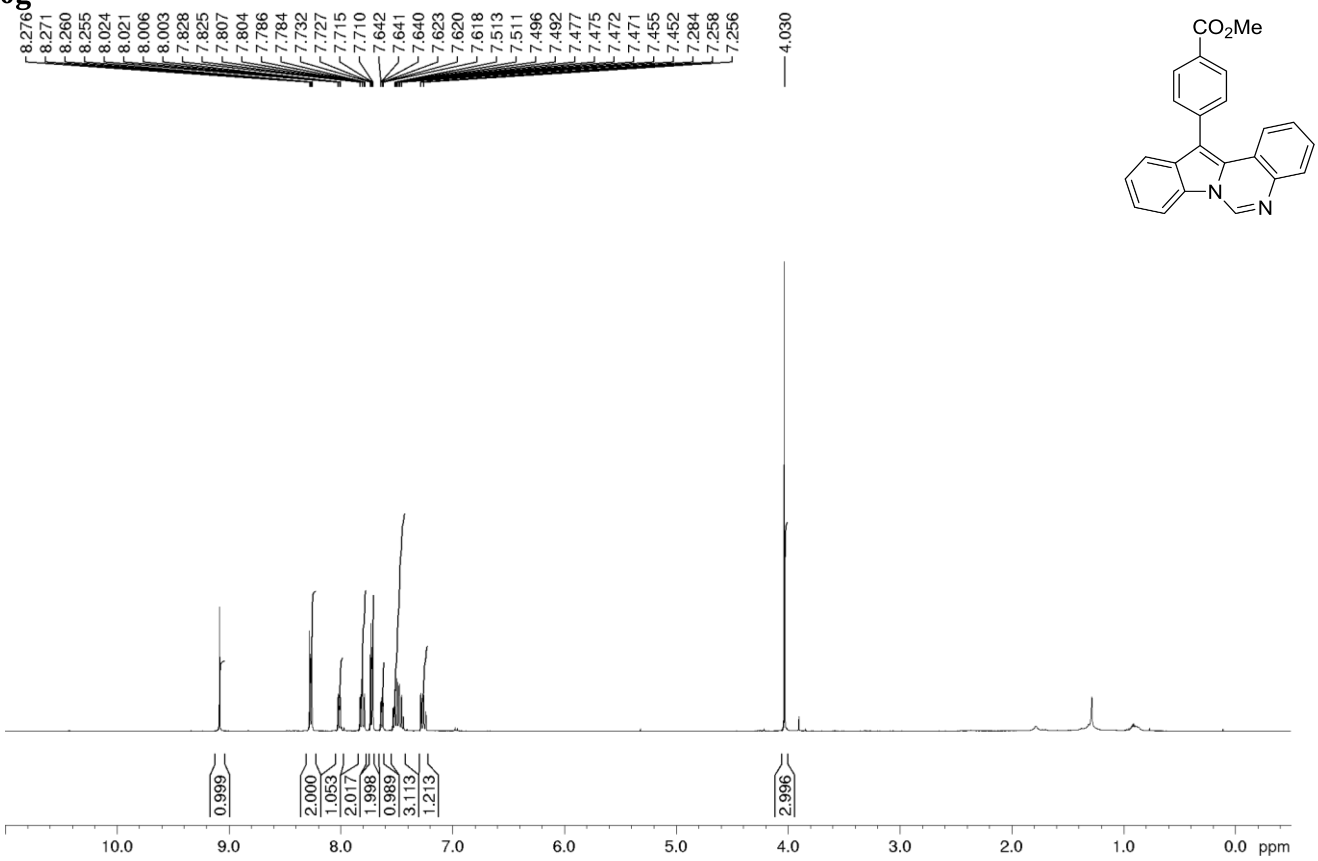
8.276
8.271
8.260
8.255
8.024
8.021
8.006
8.003
7.828
7.825
7.807
7.804
7.786
7.784
7.732
7.727
7.715
7.710
7.642
7.641
7.640
7.623
7.620
7.618
7.513
7.511
7.496
7.492
7.477
7.475
7.472
7.471
7.455
7.452
7.284
7.258
7.256
4.030
CO2Me
N
N
0.999
2.000
1.053
2.017
1.998
0.989
3.113
1.213
2.996
10.0
9.0
8.0
7.0
6.0
5.0
4.0
3.0
2.0
1.0
0.0 ppm

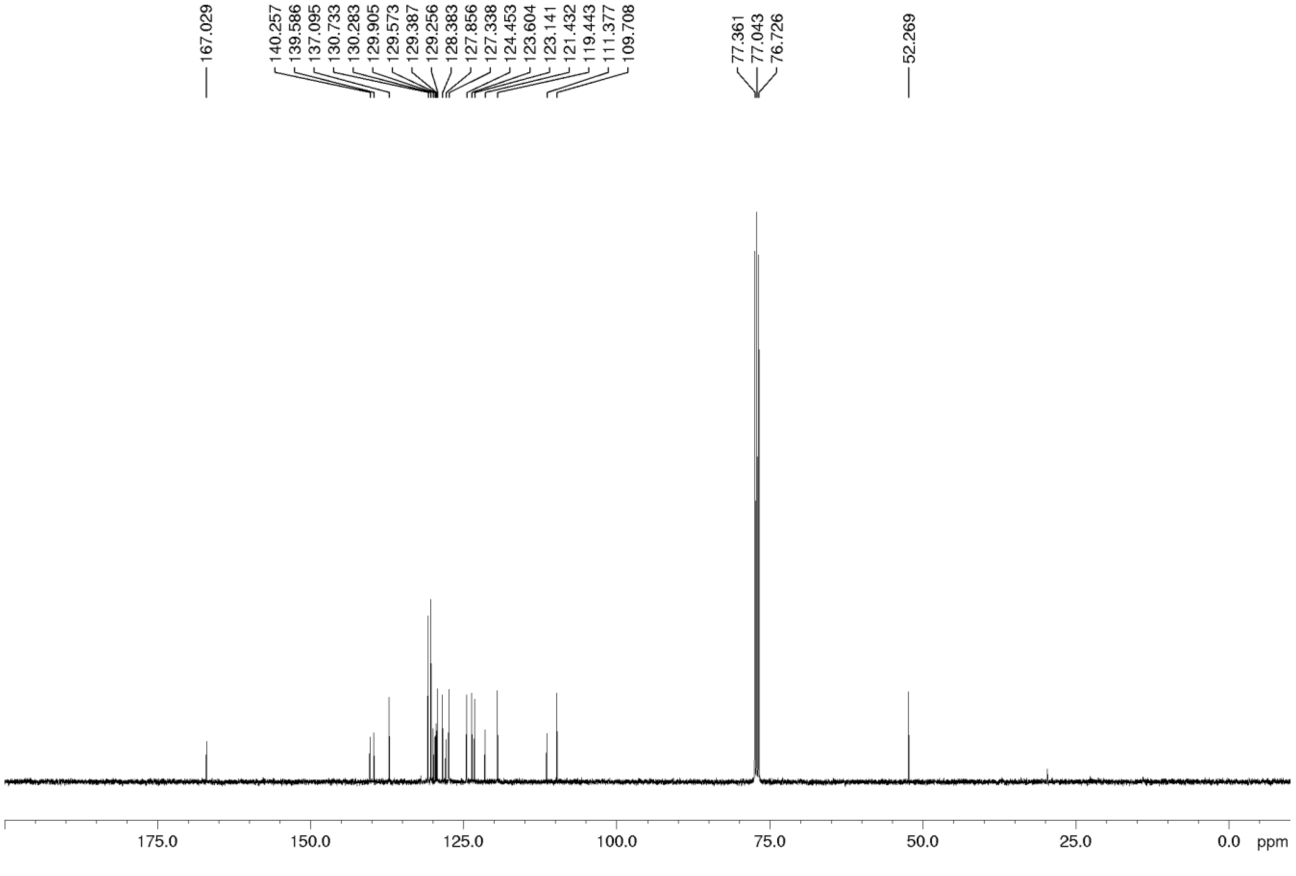
167.029
140.257
139.586
137.095
130.733
130.283
129.905
129.573
129.387
129.256
128.383
127.856
127.338
124.453
123.604
123.141
121.432
119.443
111.377
109.708
77.361
77.043
76.726
52.269
175.0
150.0
125.0
100.0
75.0
50.0
25.0
0.0 ppm

10h

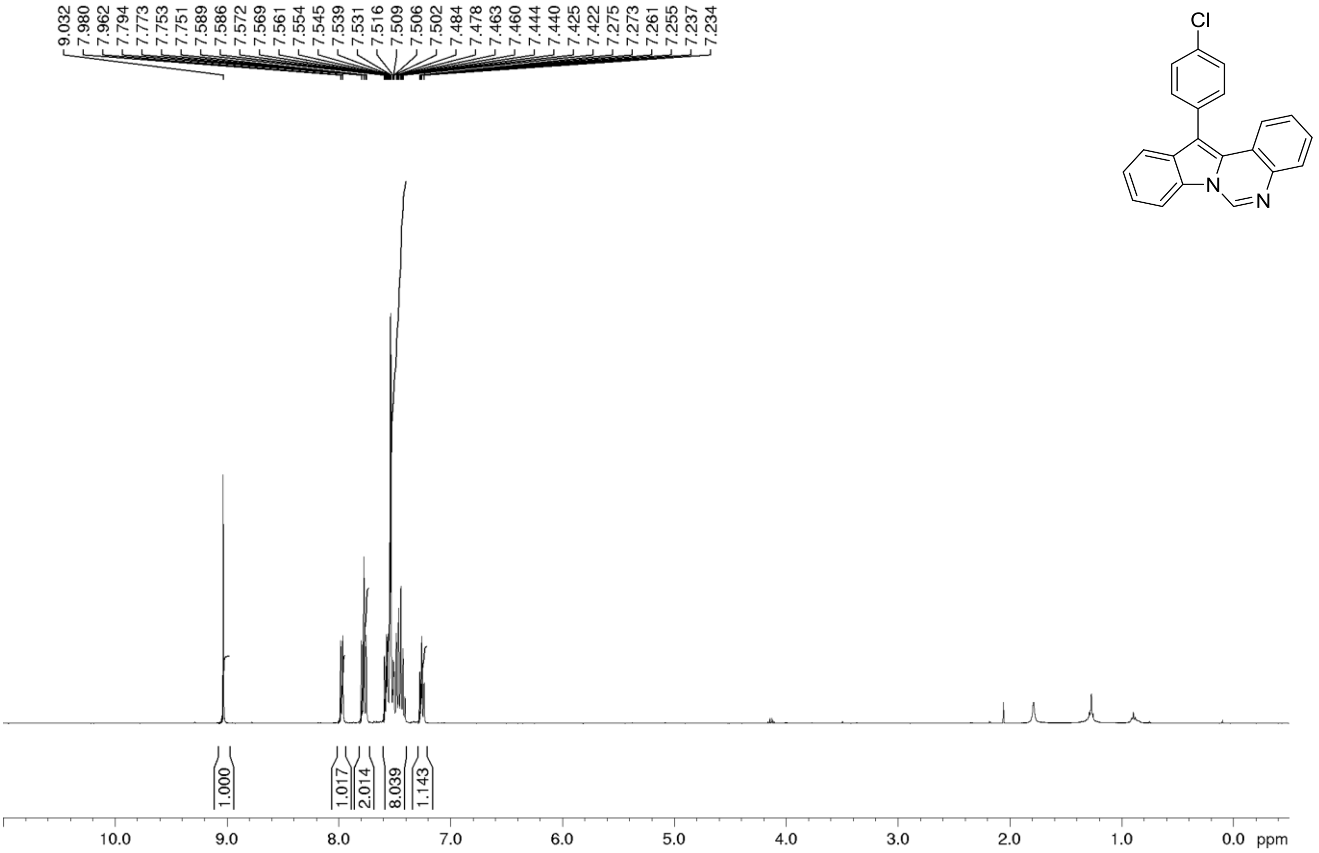
9.032
7.980
7.962
7.794
7.773
7.753
7.751
7.589
7.586
7.572
7.569
7.561
7.554
7.545
7.539
7.531
7.516
7.509
7.506
7.502
7.484
7.478
7.463
7.460
7.444
7.440
7.425
7.422
7.275
7.273
7.261
7.255
7.237
7.234
1.000
1.017
2.014
8.039
1.143
10.0
9.0
8.0
7.0
6.0
5.0
4.0
3.0
2.0
1.0
0.0 ppm

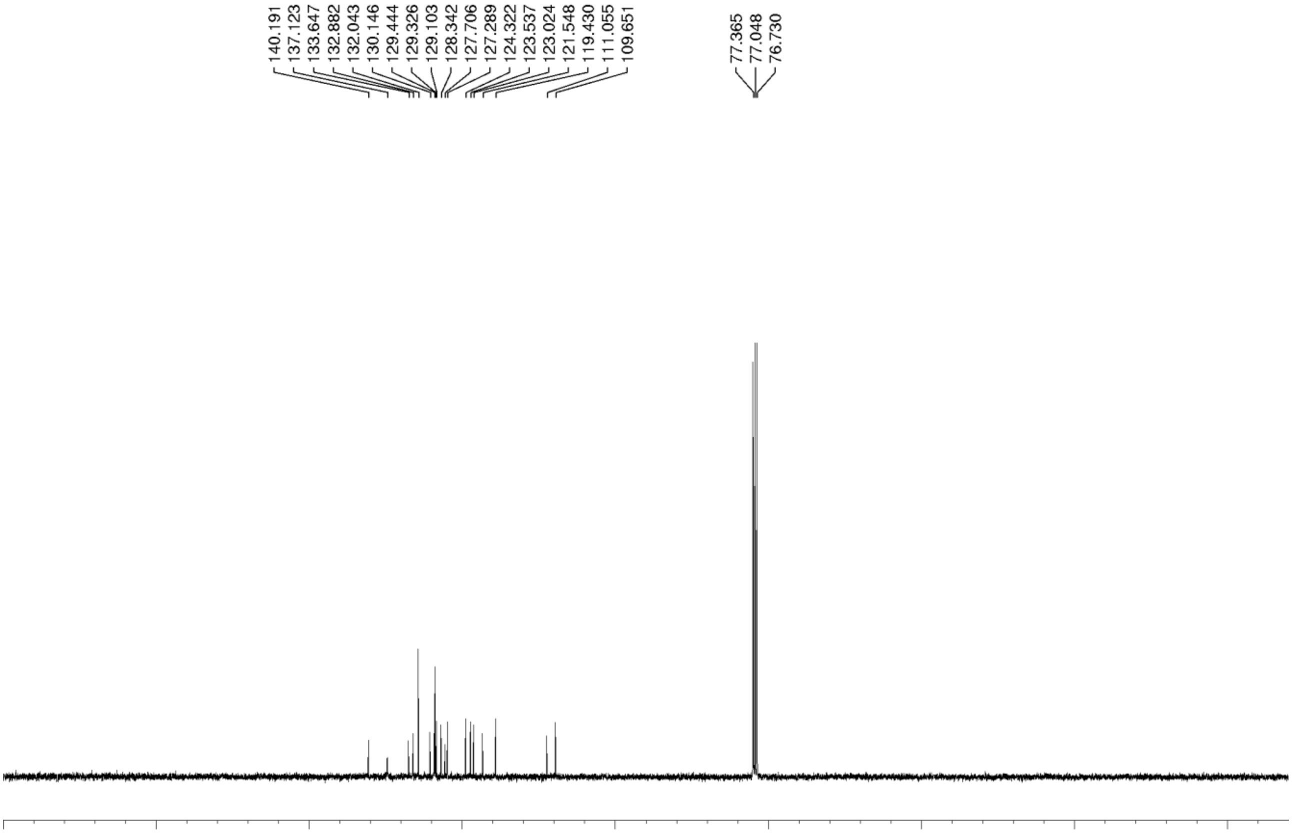
140.191
137.123
133.647
132.882
132.043
130.146
129.444
129.326
129.103
128.342
127.706
127.289
124.322
123.537
123.024
121.548
119.430
111.055
109.651
77.365
77.048
76.730
175.0
150.0
125.0
100.0
75.0
50.0
25.0
0.0 ppm

10i

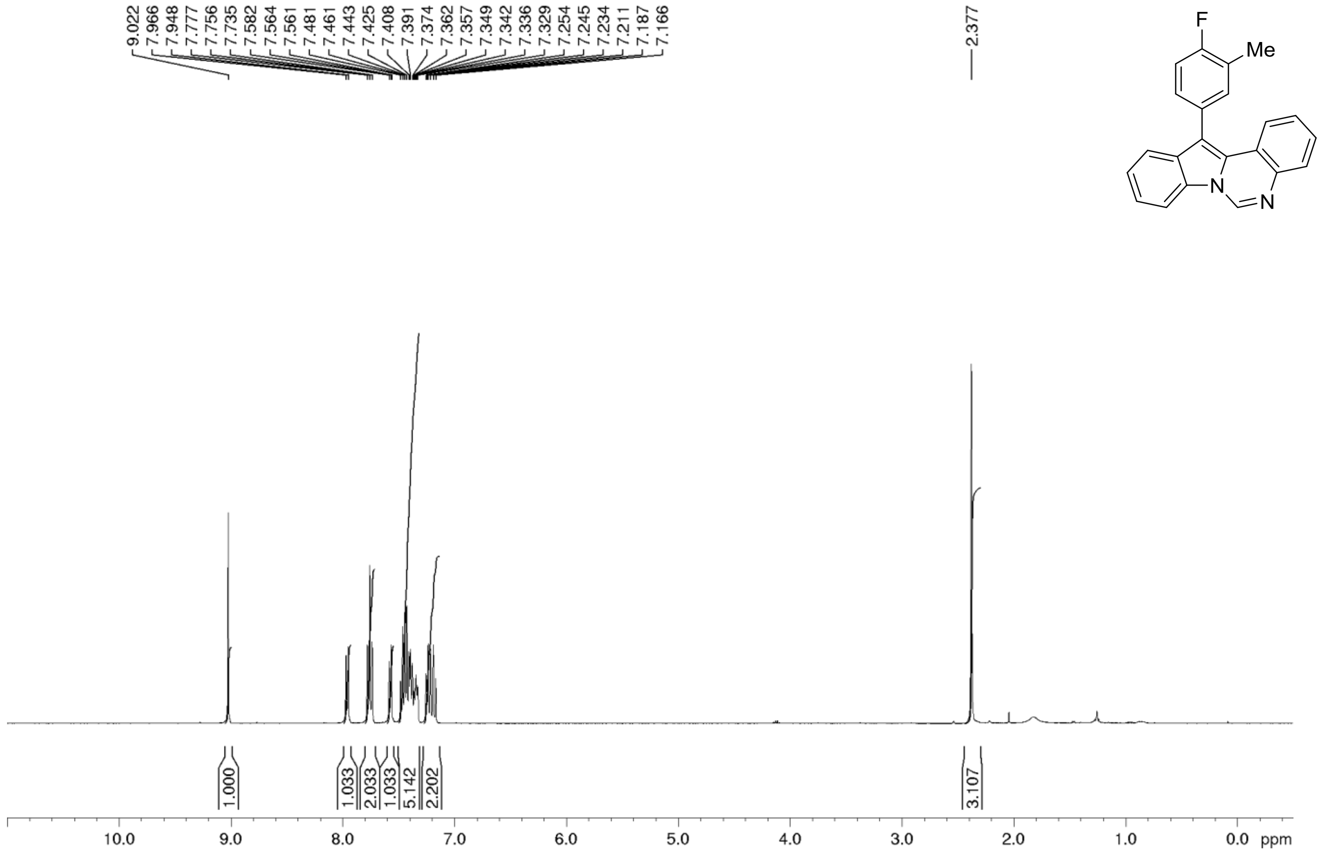
9.022
7.966
7.948
7.777
7.756
7.735
7.582
7.564
7.561
7.481
7.461
7.443
7.425
7.408
7.391
7.374
7.362
7.357
7.349
7.342
7.336
7.329
7.254
7.245
7.234
7.211
7.187
7.166
2.377
F
Me
N
N
1.000
1.033
2.033
1.033
5.142
2.202
3.107
10.0
9.0
8.0
7.0
6.0
5.0
4.0
3.0
2.0
1.0
0.0 ppm

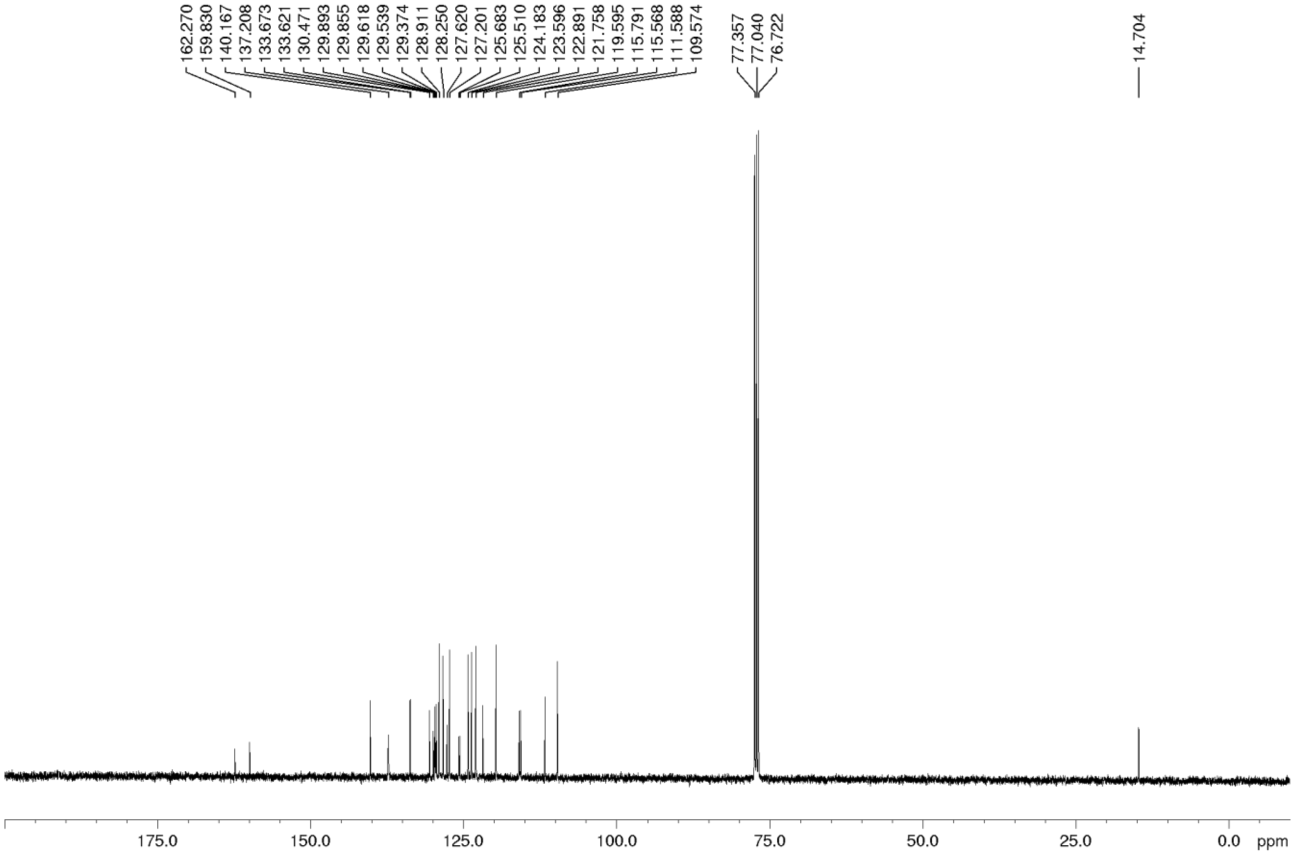
162.270
159.830
140.167
137.208
133.673
133.621
130.471
129.893
129.855
129.618
129.539
129.374
128.911
128.250
127.620
127.201
125.683
125.510
124.183
123.596
122.891
121.758
119.595
115.791
115.568
111.588
109.574
77.357
77.040
76.722
14.704
175.0
150.0
125.0
100.0
75.0
50.0
25.0
0.0 ppm

10i

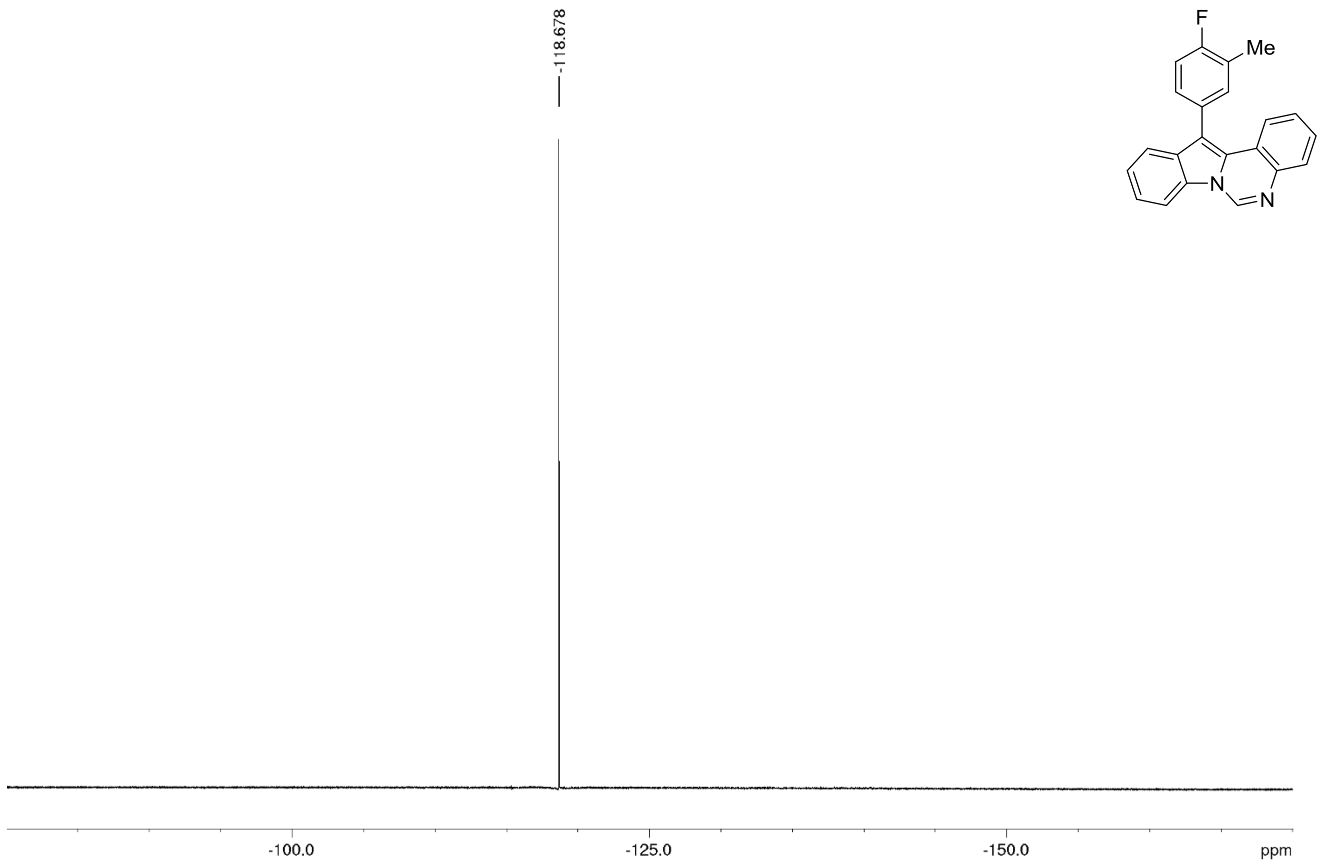

10j

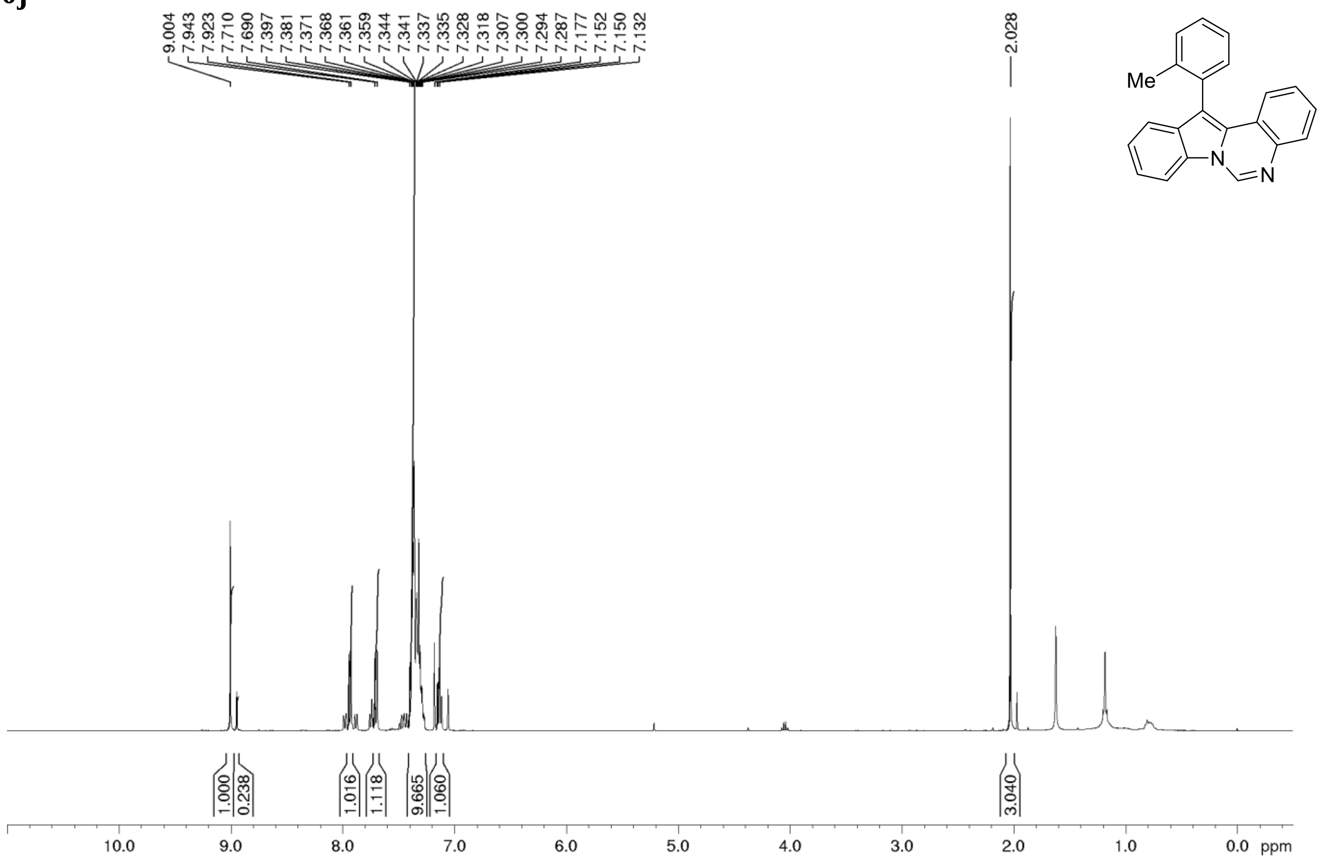
9.004
7.943
7.923
7.710
7.690
7.397
7.381
7.371
7.368
7.361
7.359
7.344
7.341
7.337
7.335
7.328
7.318
7.307
7.300
7.294
7.287
7.177
7.152
7.150
7.132
2.028
1.000
0.238
1.016
1.118
9.665
1.060
3.040
10.0
9.0
8.0
7.0
6.0
5.0
4.0
3.0
2.0
1.0
0.0
ppm
Me
N
N

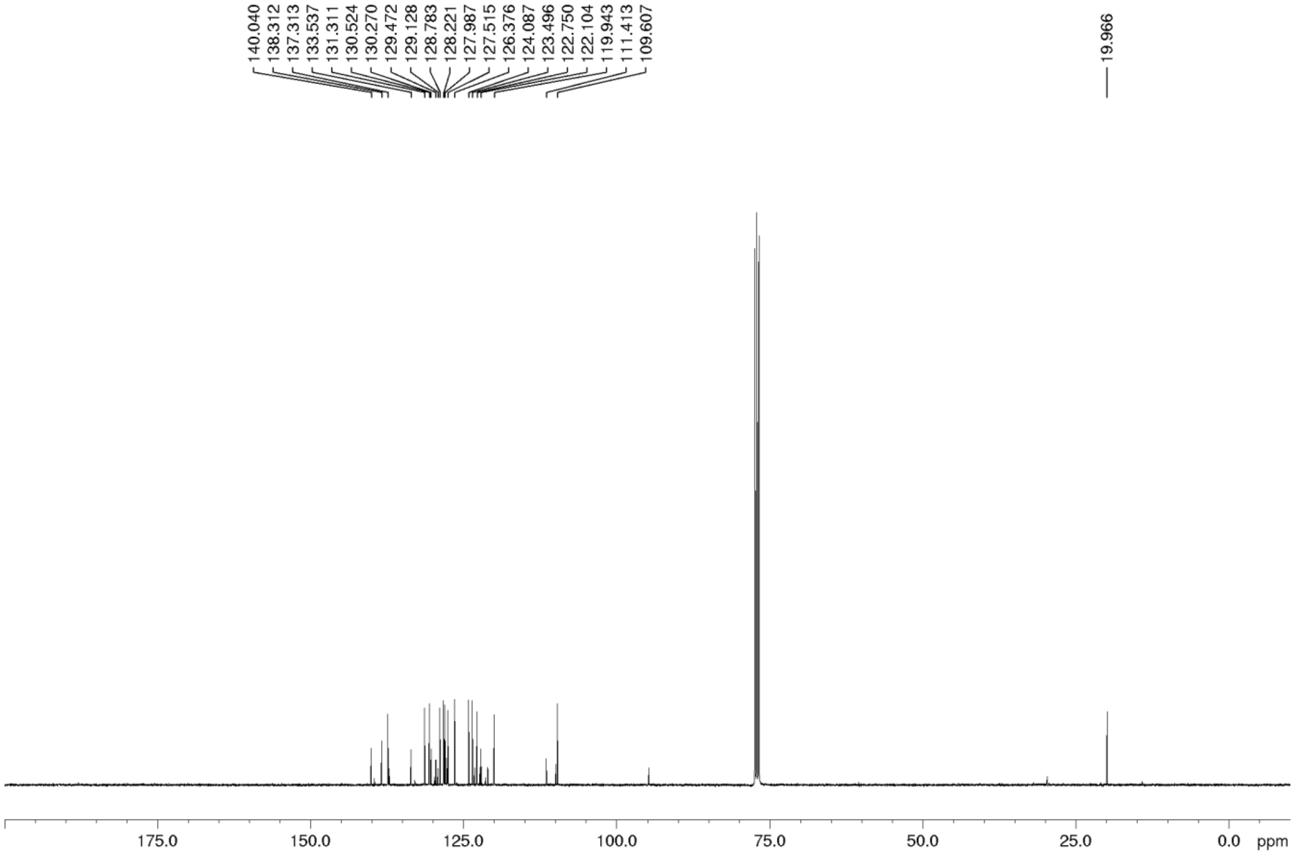
140.040
138.312
137.313
133.537
131.311
130.524
130.270
129.472
129.128
128.783
128.221
127.987
127.515
126.376
124.087
123.496
122.750
122.104
119.943
111.413
109.607
19.966
175.0
150.0
125.0
100.0
75.0
50.0
25.0
0.0
ppm

10k

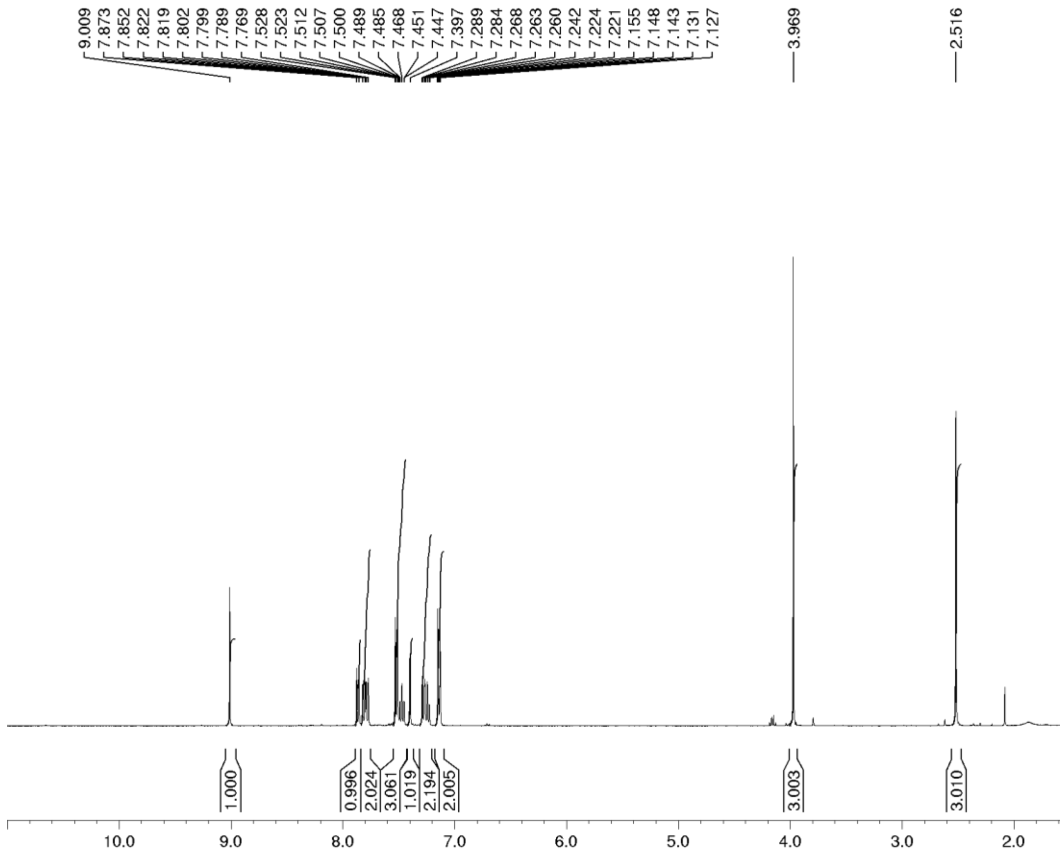

9.009
7.873
7.852
7.822
7.819
7.802
7.799
7.789
7.769
7.528
7.523
7.512
7.507
7.500
7.489
7.485
7.468
7.451
7.447
7.397
7.289
7.284
7.268
7.263
7.260
7.242
7.224
7.221
7.155
7.148
7.143
7.131
7.127
3.969
2.516
1.000
0.996
2.024
3.061
1.019
2.194
2.005
3.003
3.010
10.0
9.0
8.0
7.0
6.0
5.0
4.0
3.0
2.0
1.0
0.0 ppm

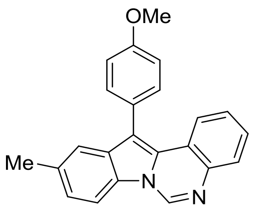

OMe
Me
N
N

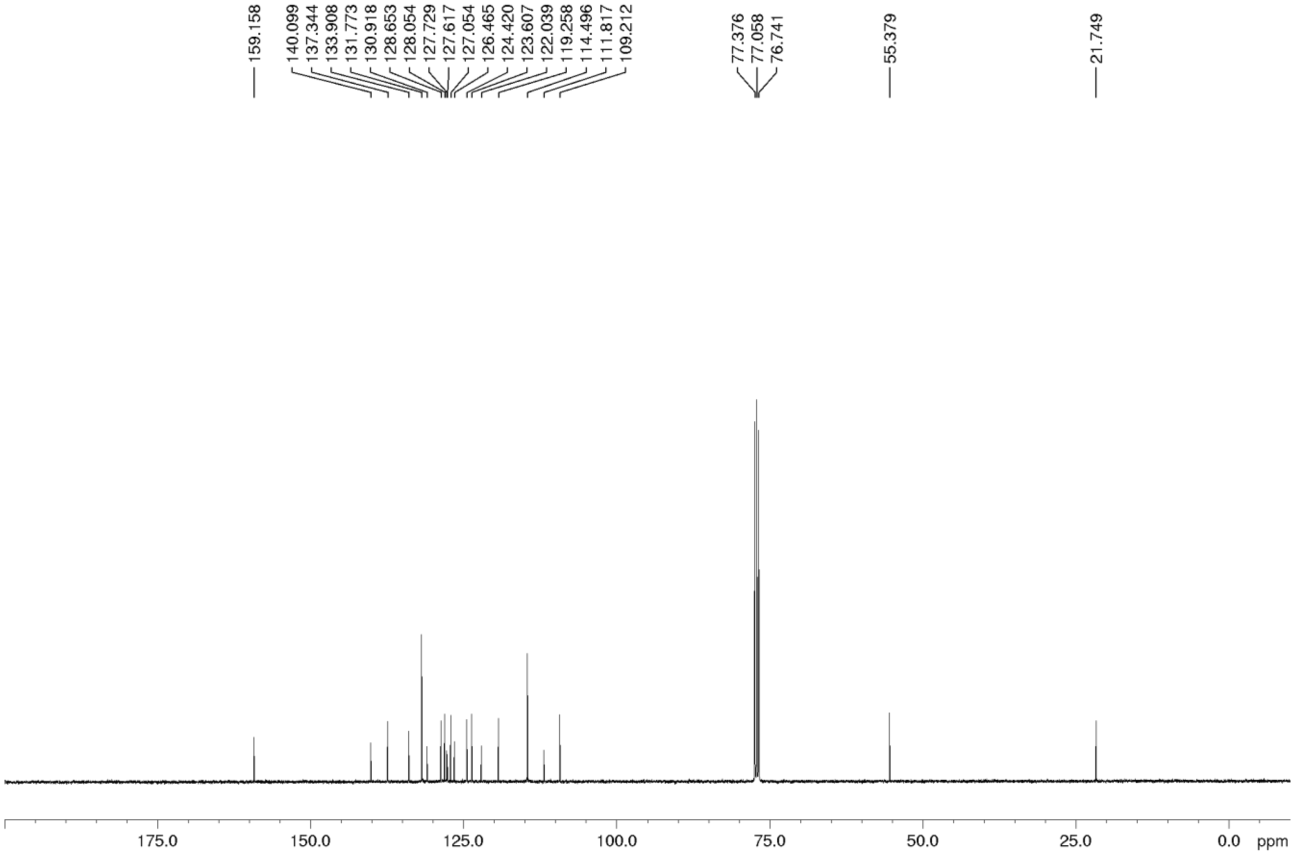

159.158
140.099
137.344
133.908
131.773
130.918
128.653
128.054
127.729
127.617
127.054
126.465
124.420
123.607
122.039
119.258
114.496
111.817
109.212
77.376
77.058
76.741
55.379
21.749
175.0
150.0
125.0
100.0
75.0
50.0
25.0
0.0 ppm

10l

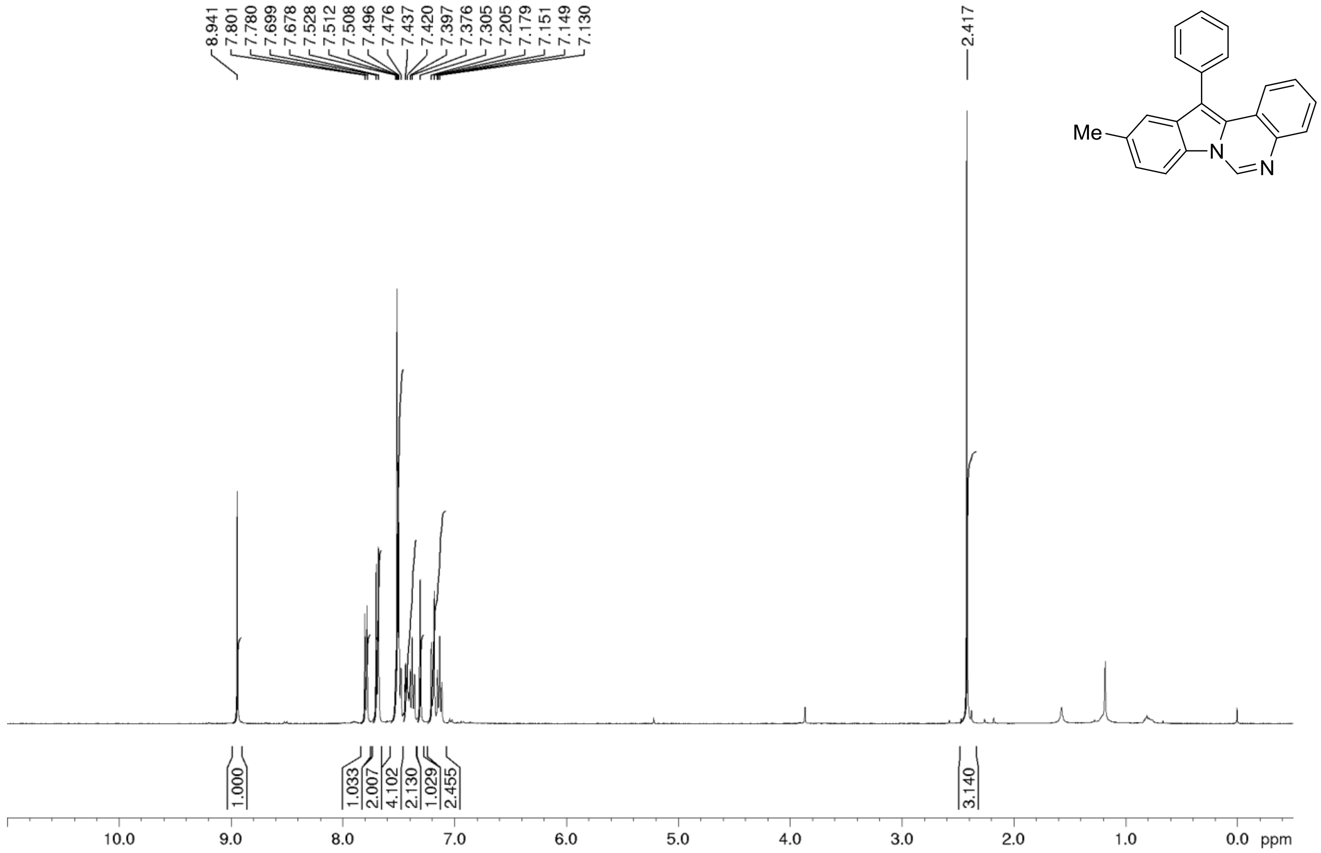

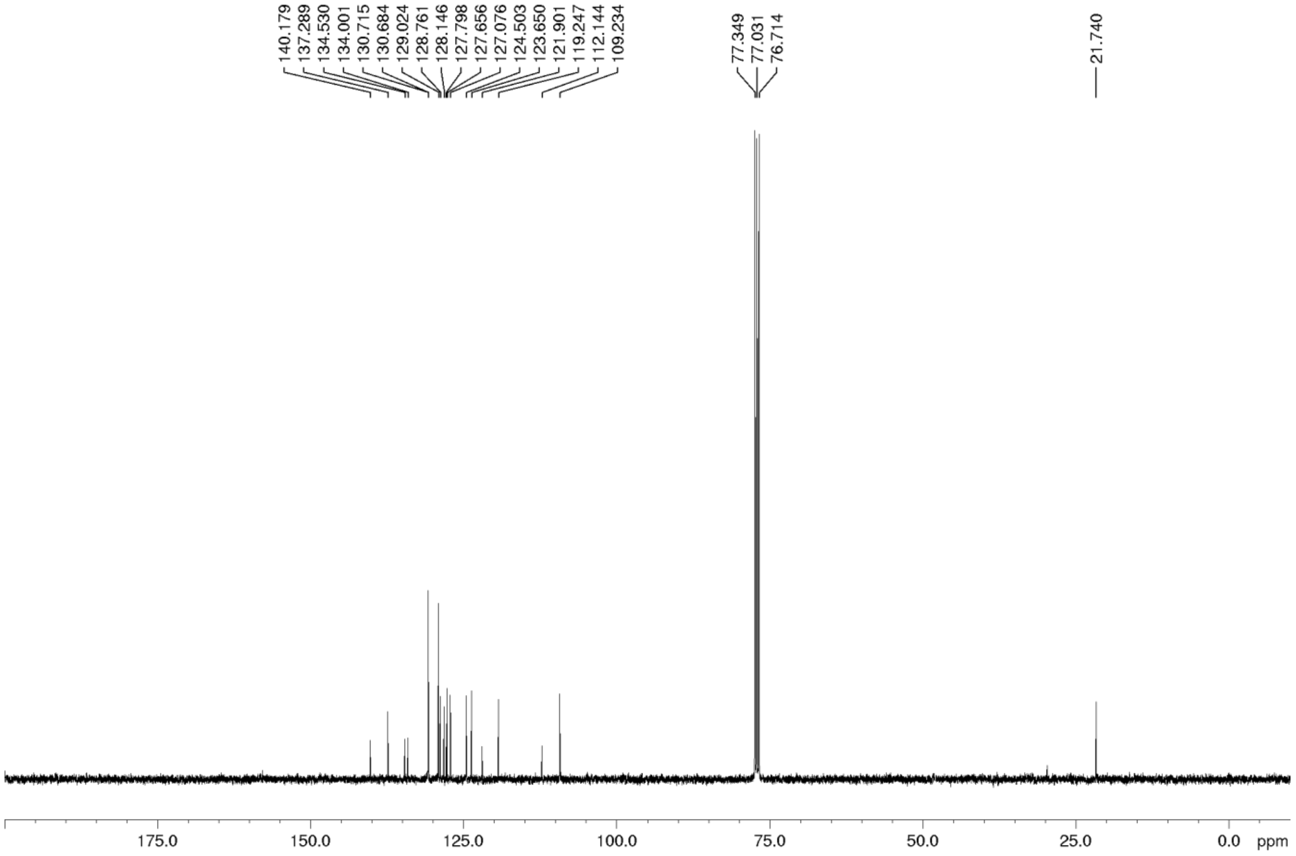

10m

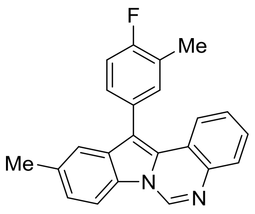
F
Me
Me
N
N

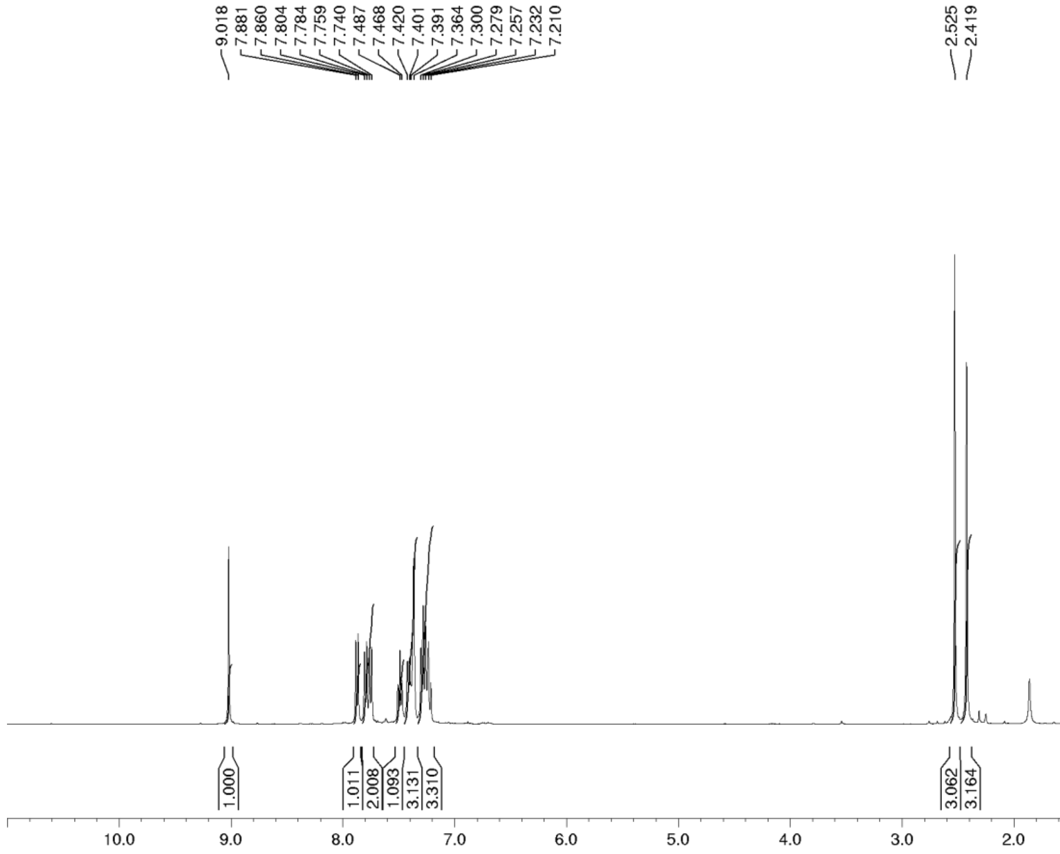
9.018
7.881
7.860
7.804
7.784
7.759
7.740
7.487
7.468
7.420
7.401
7.391
7.364
7.300
7.279
7.257
7.232
7.210
2.525
2.419
1.000
1.011
2.008
1.093
3.131
3.310
3.062
3.164
10.0
9.0
8.0
7.0
6.0
5.0
4.0
3.0
2.0
1.0
0.0 ppm

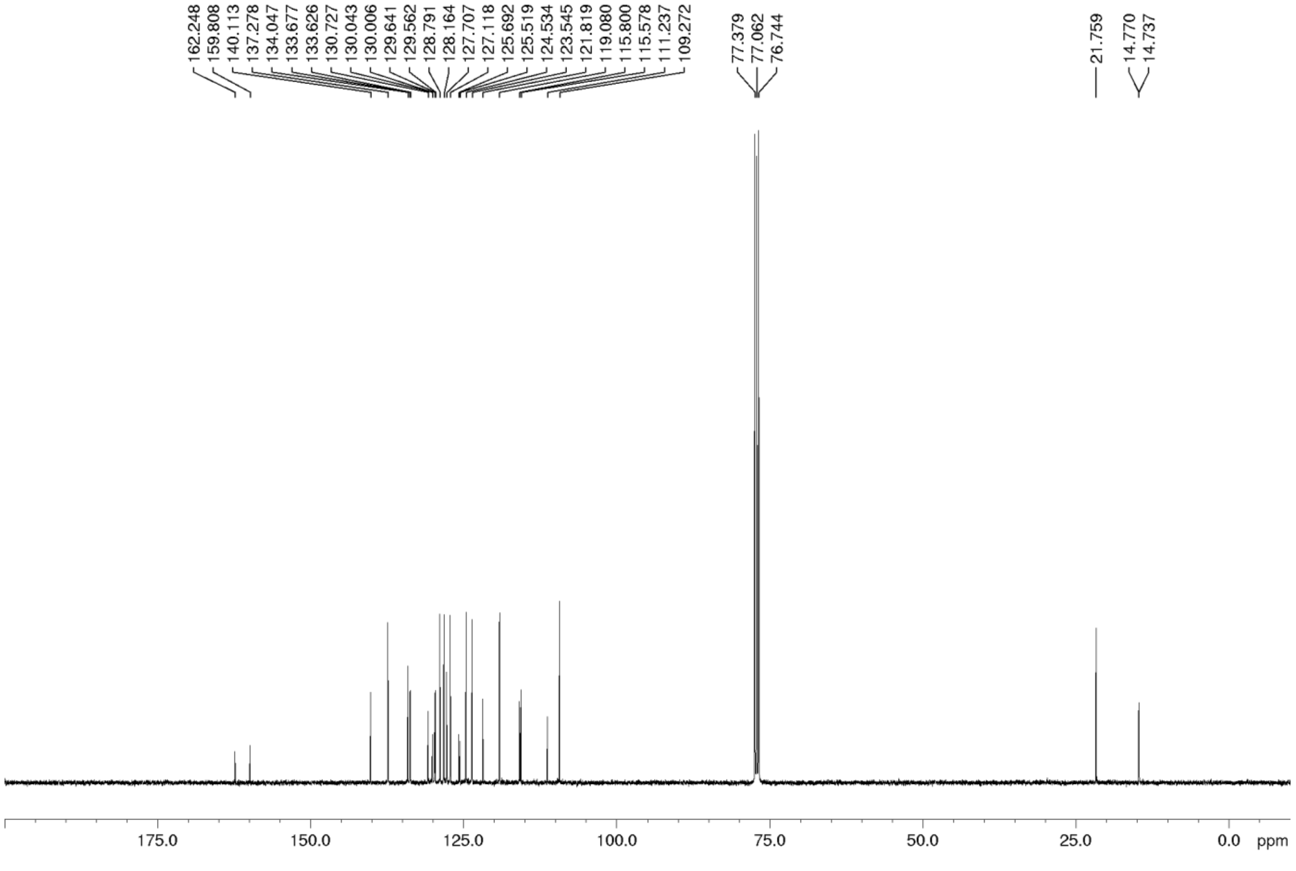
162.248
159.808
140.113
137.278
134.047
133.677
133.626
130.727
130.043
130.006
129.641
129.562
128.791
128.164
127.707
127.118
125.692
125.519
124.534
123.545
121.819
119.080
115.800
115.578
111.237
109.272
77.379
77.062
76.744
21.759
14.770
14.737
175.0
150.0
125.0
100.0
75.0
50.0
25.0
0.0 ppm

10m

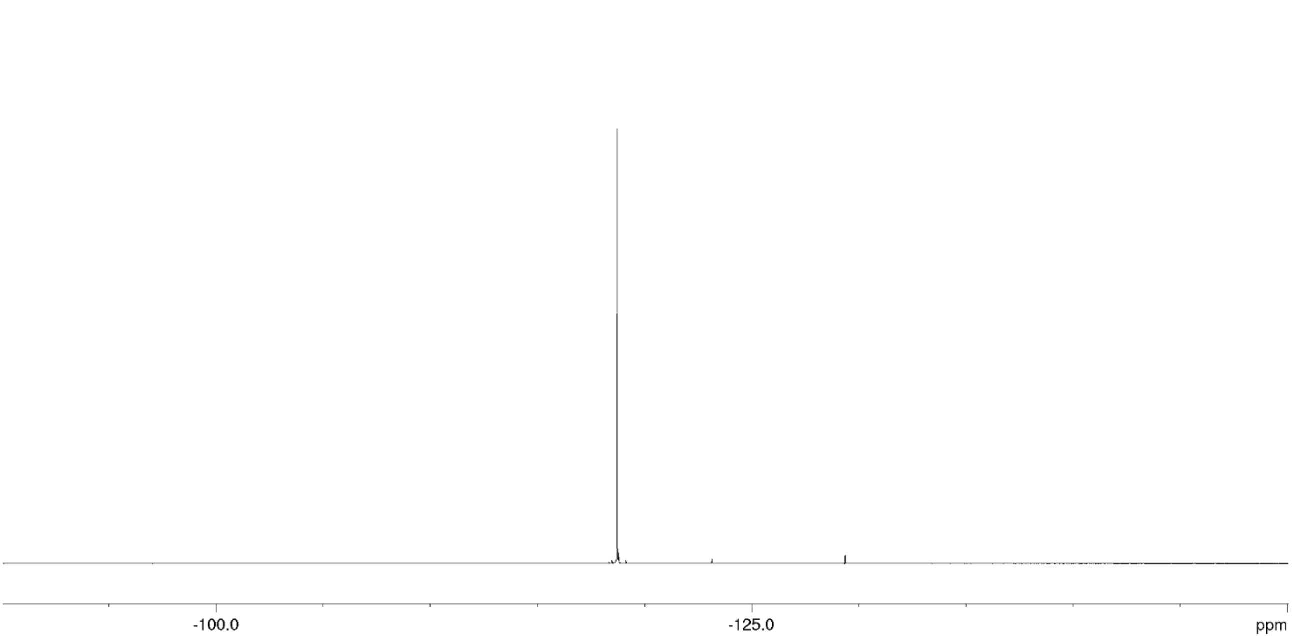

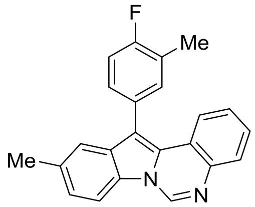

10n

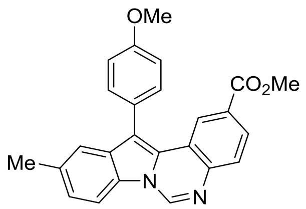
OMe
CO_2Me
Me
N
N

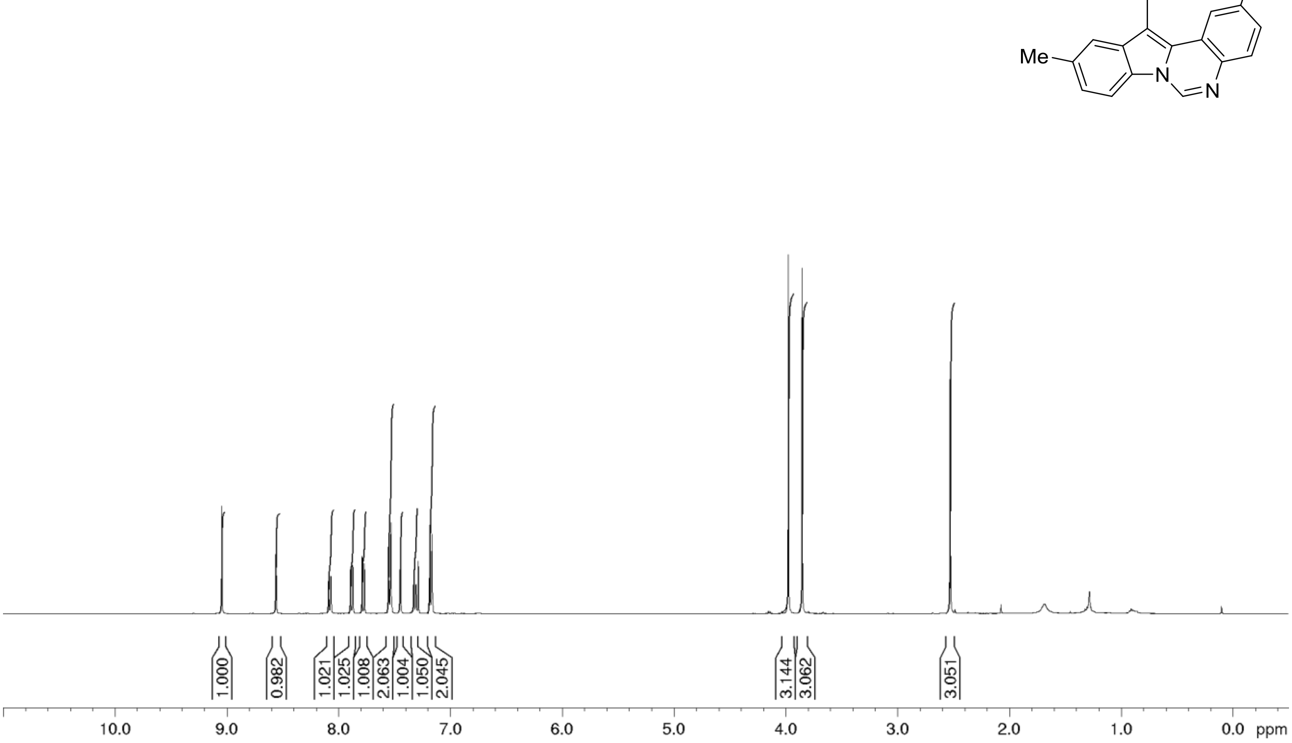
9.043
8.560
8.555
8.090
8.085
8.069
8.064
7.891
7.870
7.788
7.767
7.551
7.546
7.534
7.529
7.445
7.326
7.324
7.305
7.303
7.284
7.183
7.161
3.975
3.851
2.527
1.000
0.982
1.021
1.025
1.008
2.063
1.004
1.050
2.045
3.144
3.062
3.051
10.0
9.0
8.0
7.0
6.0
5.0
4.0
3.0
2.0
1.0
0.0 ppm

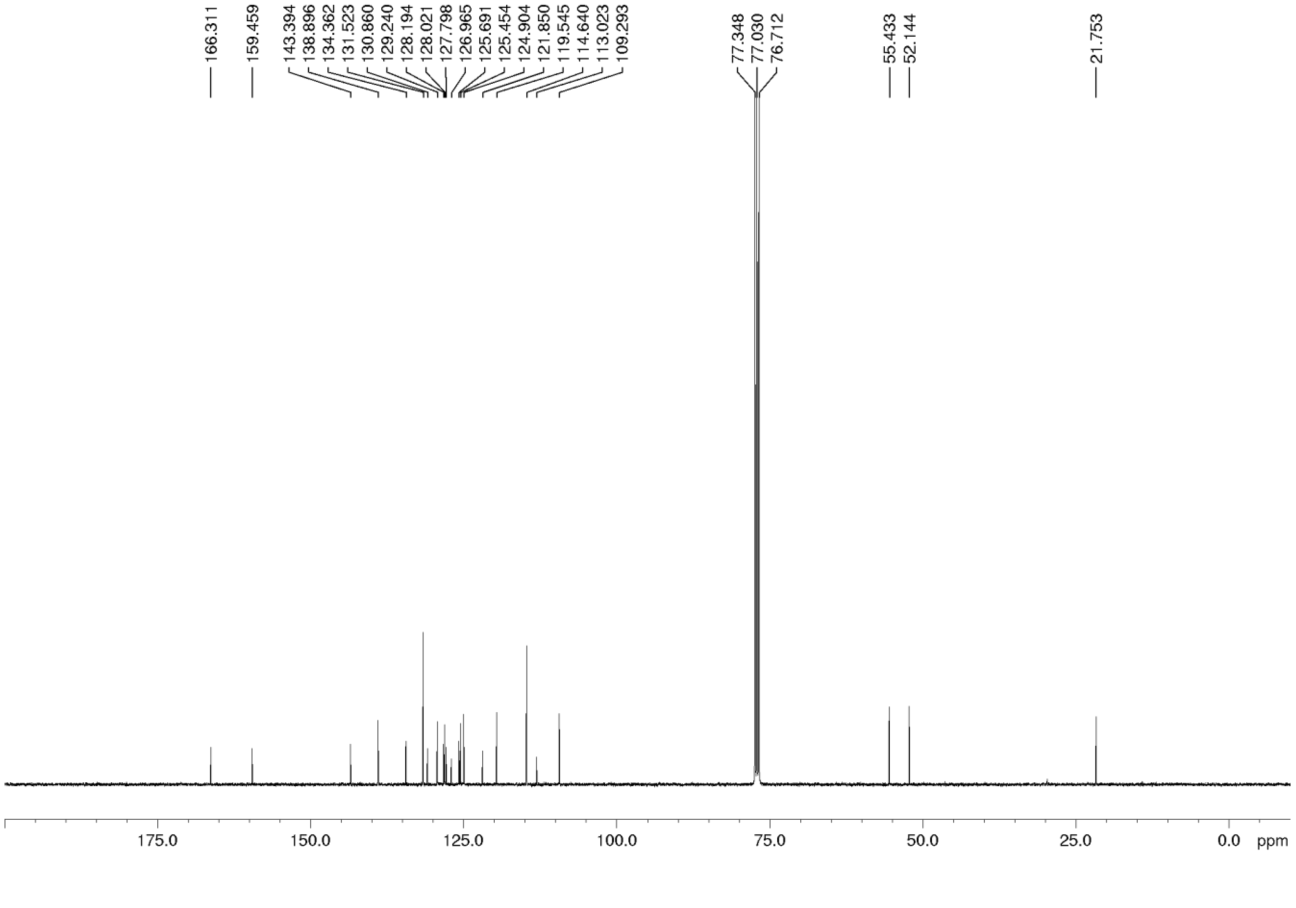
166.311
159.459
143.394
138.896
134.362
131.523
130.860
129.240
128.194
128.021
127.798
126.965
125.691
125.454
124.904
121.850
119.545
114.640
113.023
109.293
77.348
77.030
76.712
55.433
52.144
21.753
175.0
150.0
125.0
100.0
75.0
50.0
25.0
0.0 ppm

10o

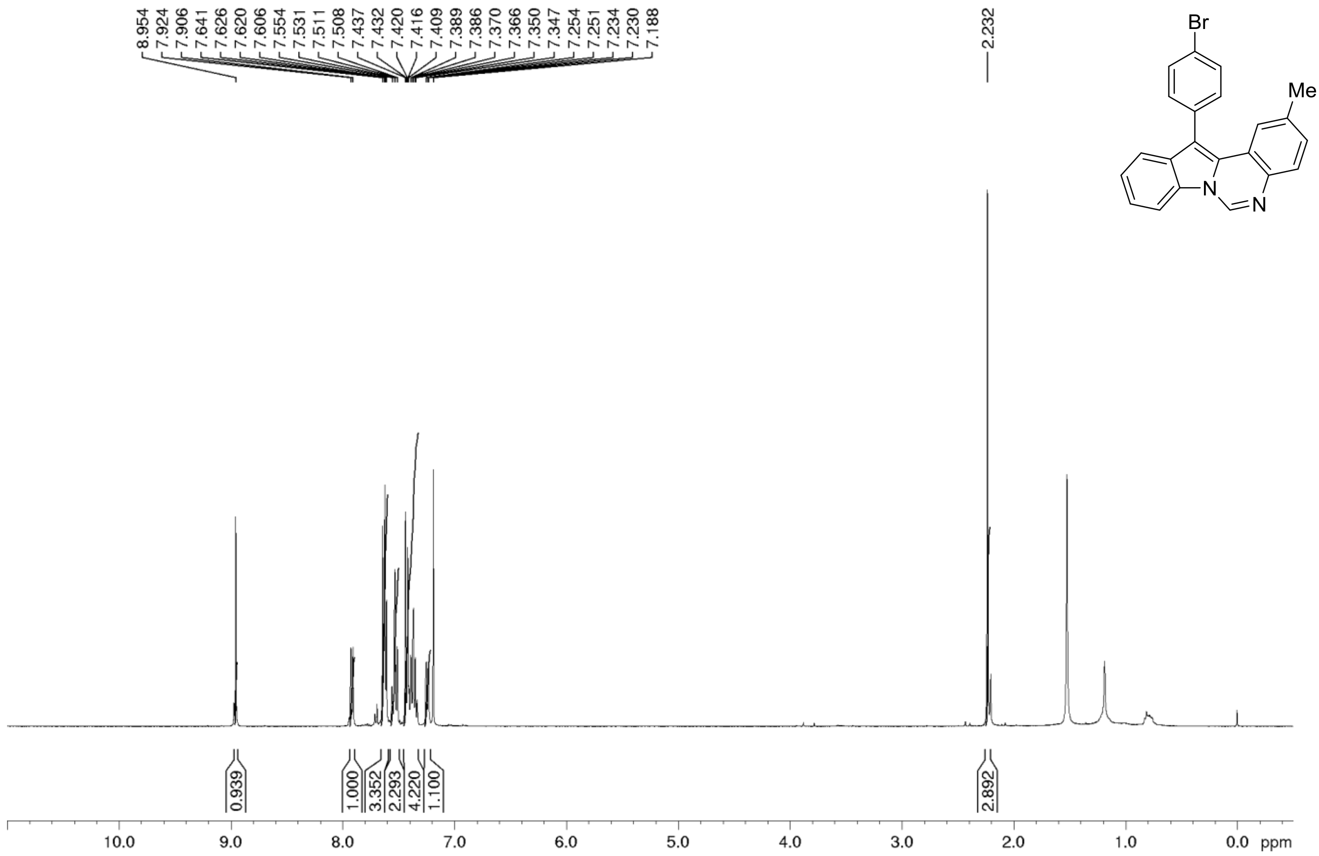

8.954
7.924
7.906
7.641
7.626
7.620
7.606
7.554
7.531
7.511
7.508
7.437
7.432
7.420
7.416
7.409
7.389
7.386
7.370
7.366
7.350
7.347
7.254
7.251
7.234
7.230
7.188
2.232
0.939
1.000
3.352
2.293
4.220
1.100
2.892
10.0
9.0
8.0
7.0
6.0
5.0
4.0
3.0
2.0
1.0
0.0 ppm
Br
Me
N
N

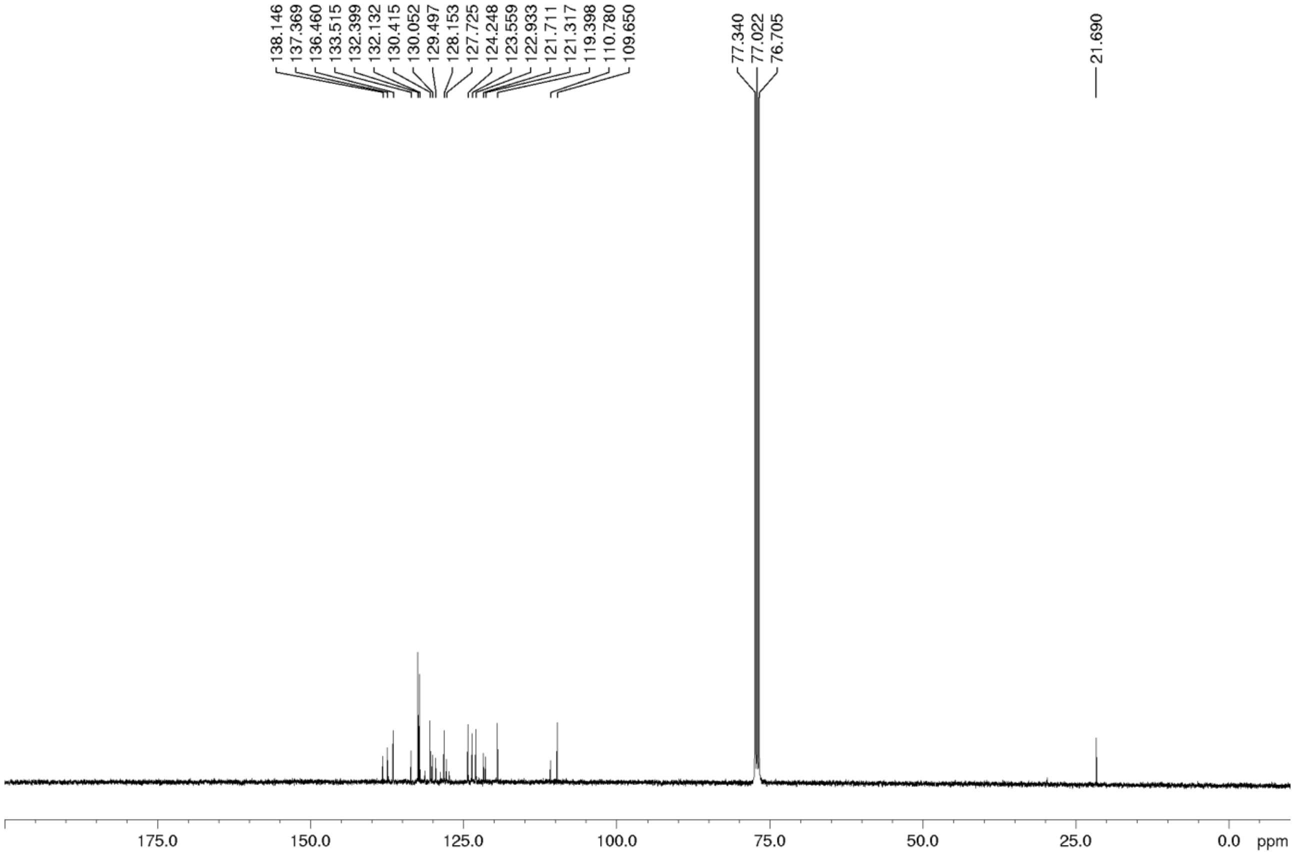

138.146
137.369
136.460
133.515
132.399
132.132
130.415
130.052
129.497
128.153
127.725
124.248
123.559
122.933
121.711
121.317
119.398
110.780
109.650
77.340
77.022
76.705
21.690
175.0
150.0
125.0
100.0
75.0
50.0
25.0
0.0 ppm

10p

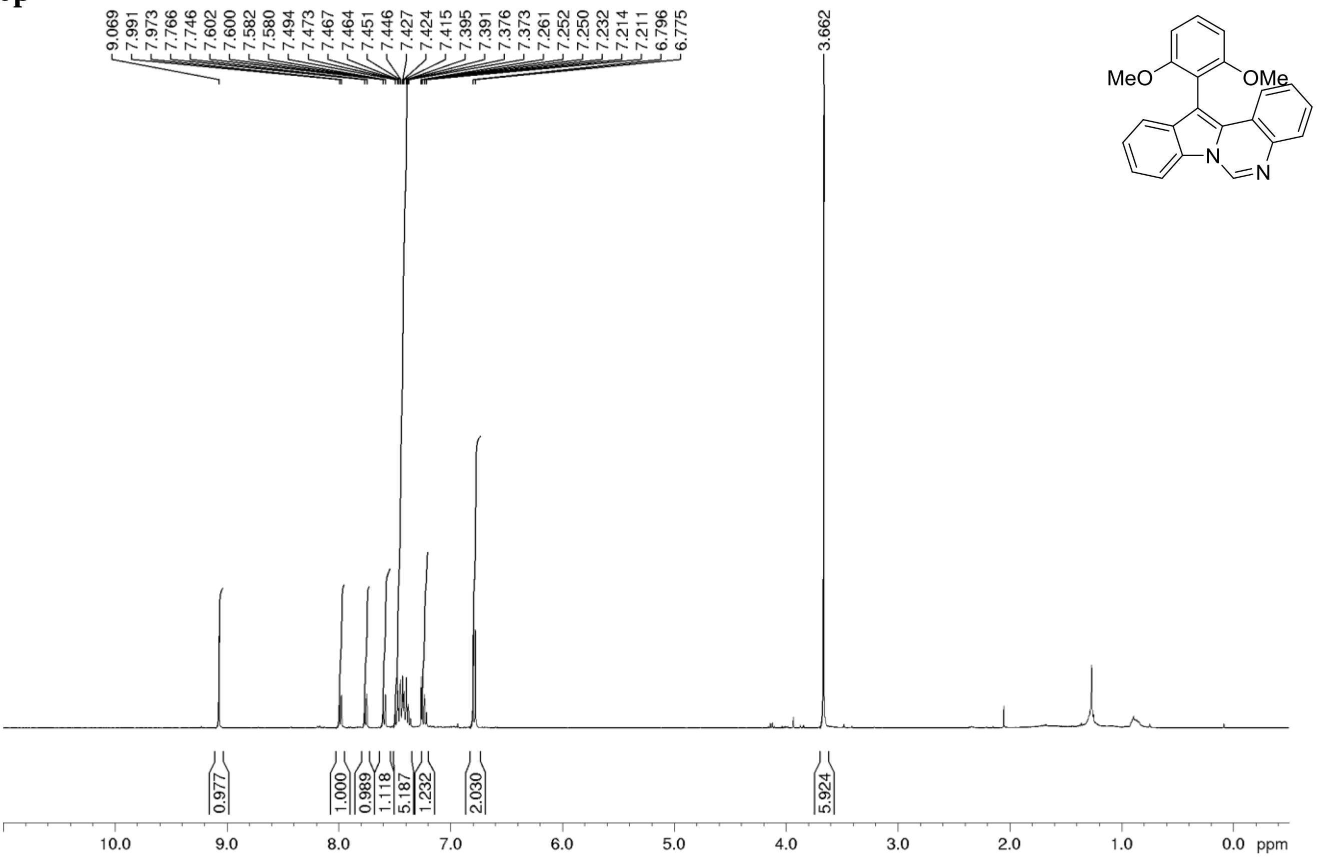
9.069
7.991
7.973
7.766
7.746
7.602
7.600
7.582
7.580
7.494
7.473
7.467
7.464
7.451
7.446
7.427
7.424
7.415
7.395
7.391
7.376
7.373
7.261
7.252
7.250
7.232
7.214
7.211
6.796
6.775
3.662
MeO
OMe
N
N
0.977
1.000
0.989
1.118
5.187
1.232
2.030
5.924
10.0
9.0
8.0
7.0
6.0
5.0
4.0
3.0
2.0
1.0
0.0
ppm

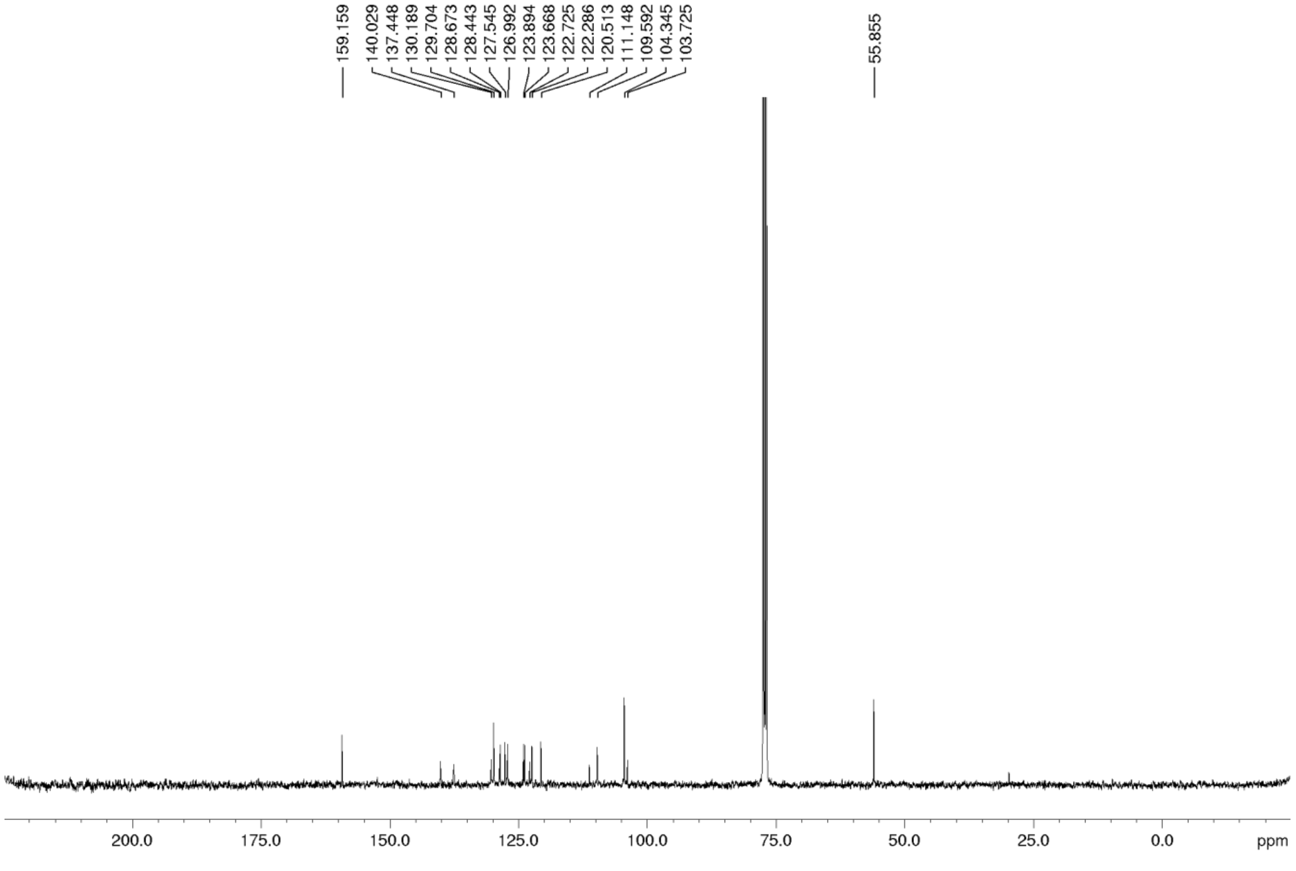
159.159
140.029
137.448
130.189
129.704
128.673
128.443
127.545
126.992
123.894
123.668
122.725
122.286
120.513
111.148
109.592
104.345
103.725
55.855
200.0
175.0
150.0
125.0
100.0
75.0
50.0
25.0
0.0
ppm

13a

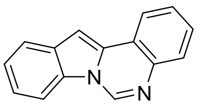

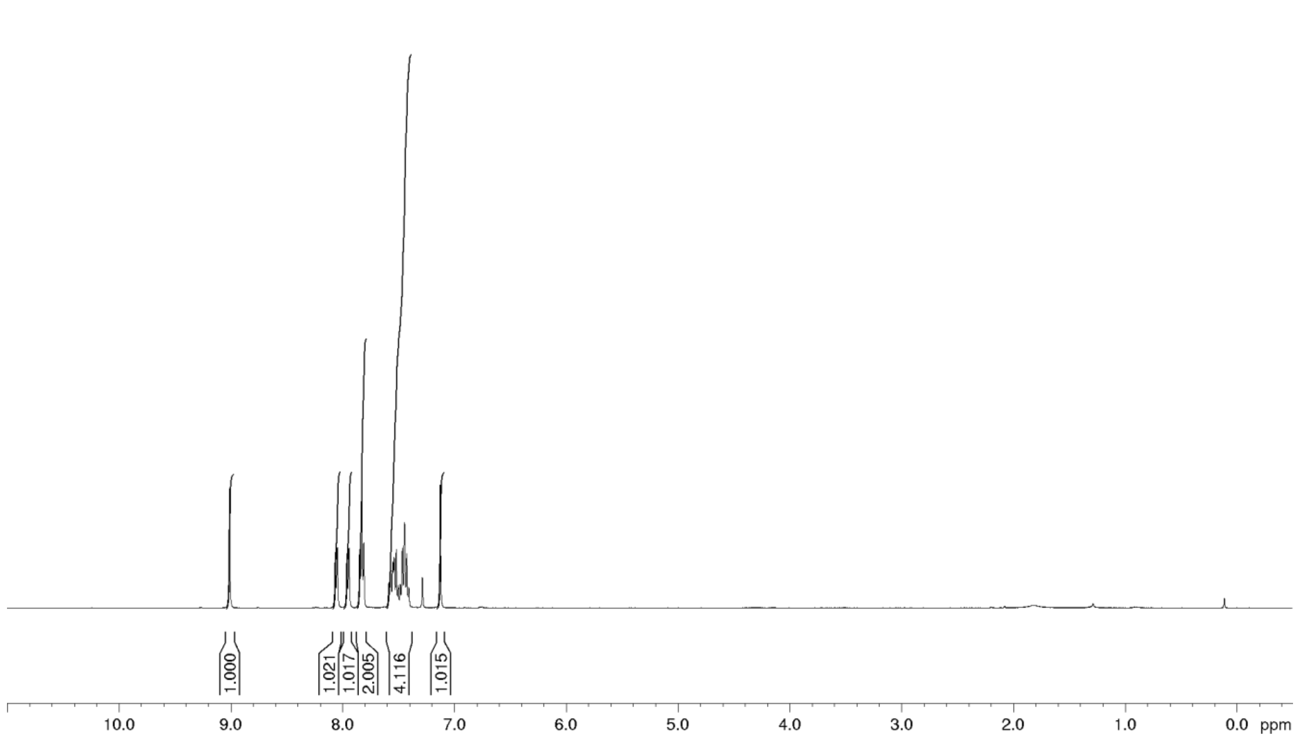
9.010
8.063
8.044
7.959
7.940
7.845
7.828
7.809
7.585
7.567
7.548
7.536
7.517
7.499
7.481
7.463
7.445
7.423
7.405
7.284
7.125
1.000
1.021
1.017
2.005
4.116
1.015
10.0
9.0
8.0
7.0
6.0
5.0
4.0
3.0
2.0
1.0
0.0 ppm

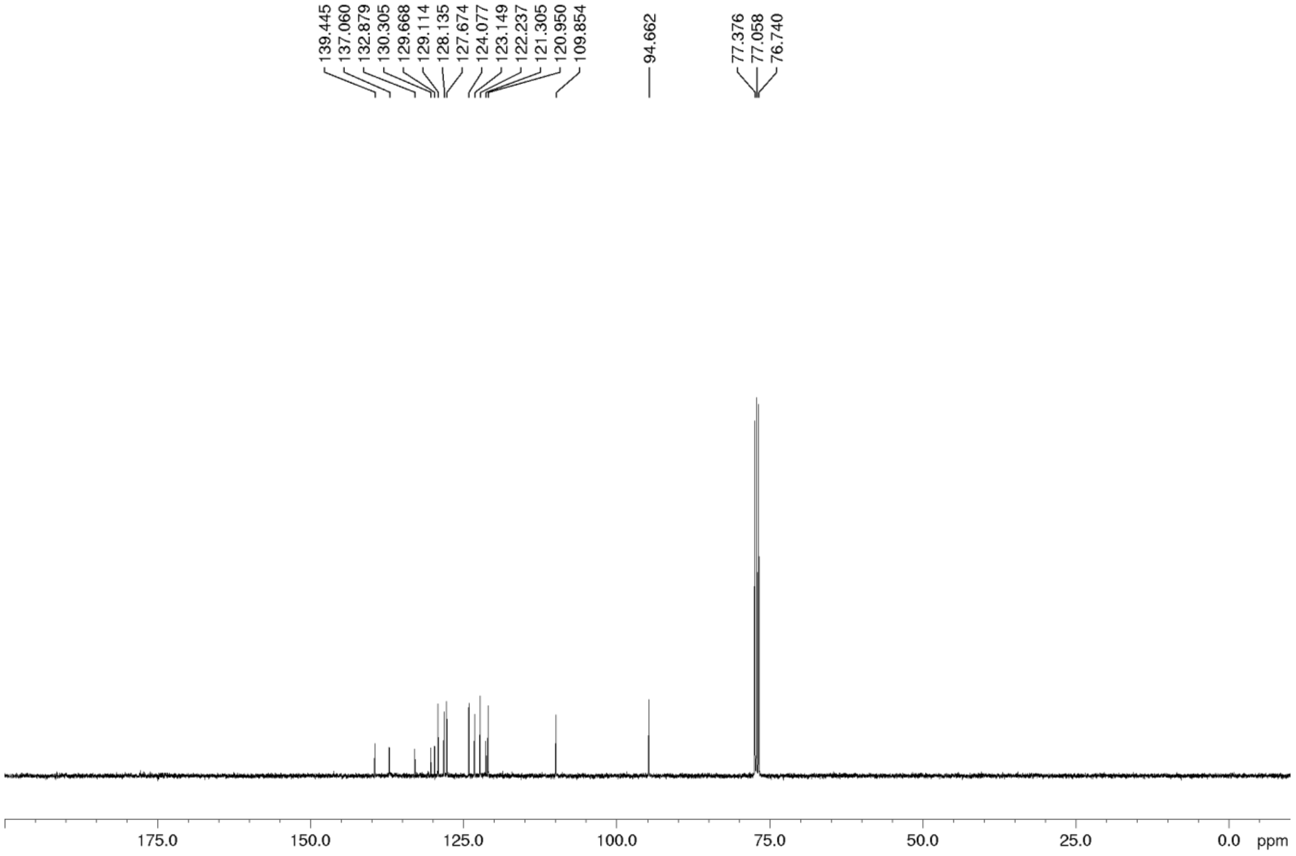
139.445
137.060
132.879
130.305
129.668
129.114
128.135
127.674
124.077
123.149
122.237
121.305
120.950
109.854
94.662
77.376
77.058
76.740
175.0
150.0
125.0
100.0
75.0
50.0
25.0
0.0 ppm

13b

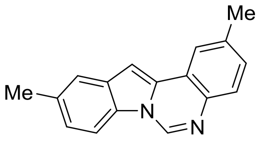
Me
Me
N
N

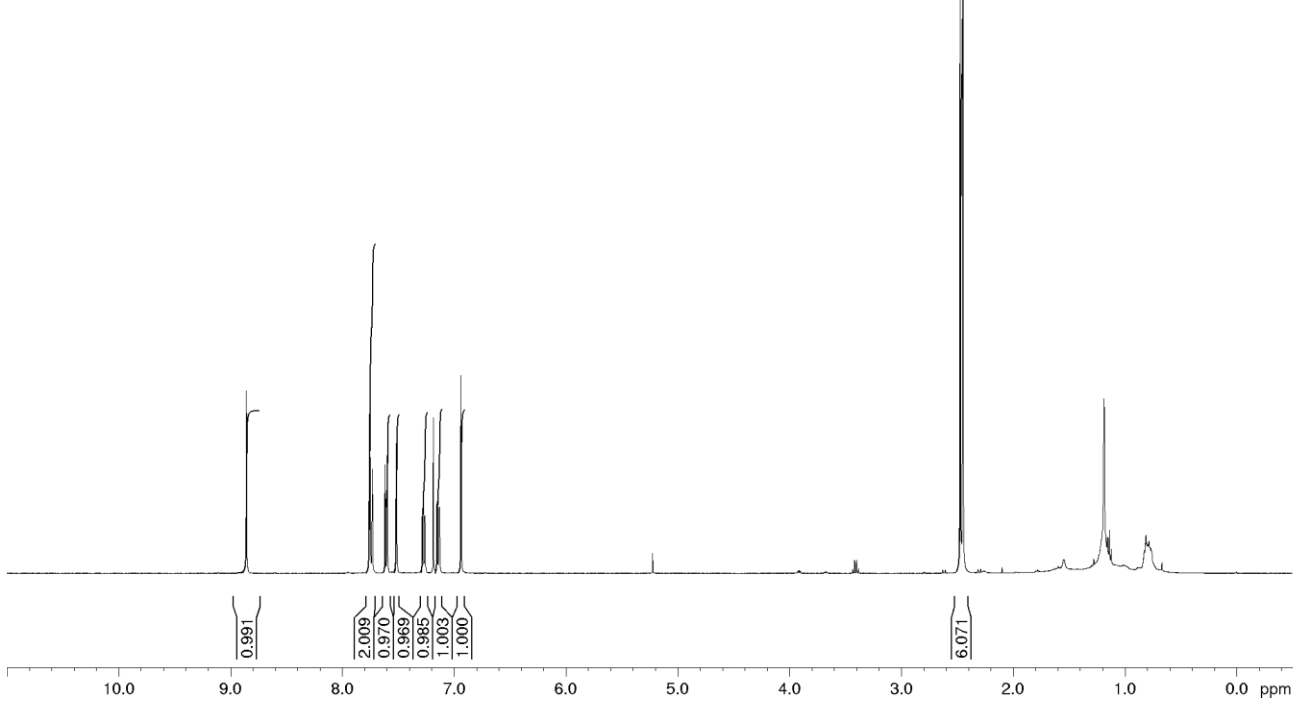
8.856
7.752
7.730
7.616
7.595
7.515
7.282
7.278
7.184
7.152
7.149
7.131
7.128
6.936
2.471
2.446
0.991
2.009
0.970
0.969
0.985
1.003
1.000
6.071
10.0
9.0
8.0
7.0
6.0
5.0
4.0
3.0
2.0
1.0
0.0
ppm

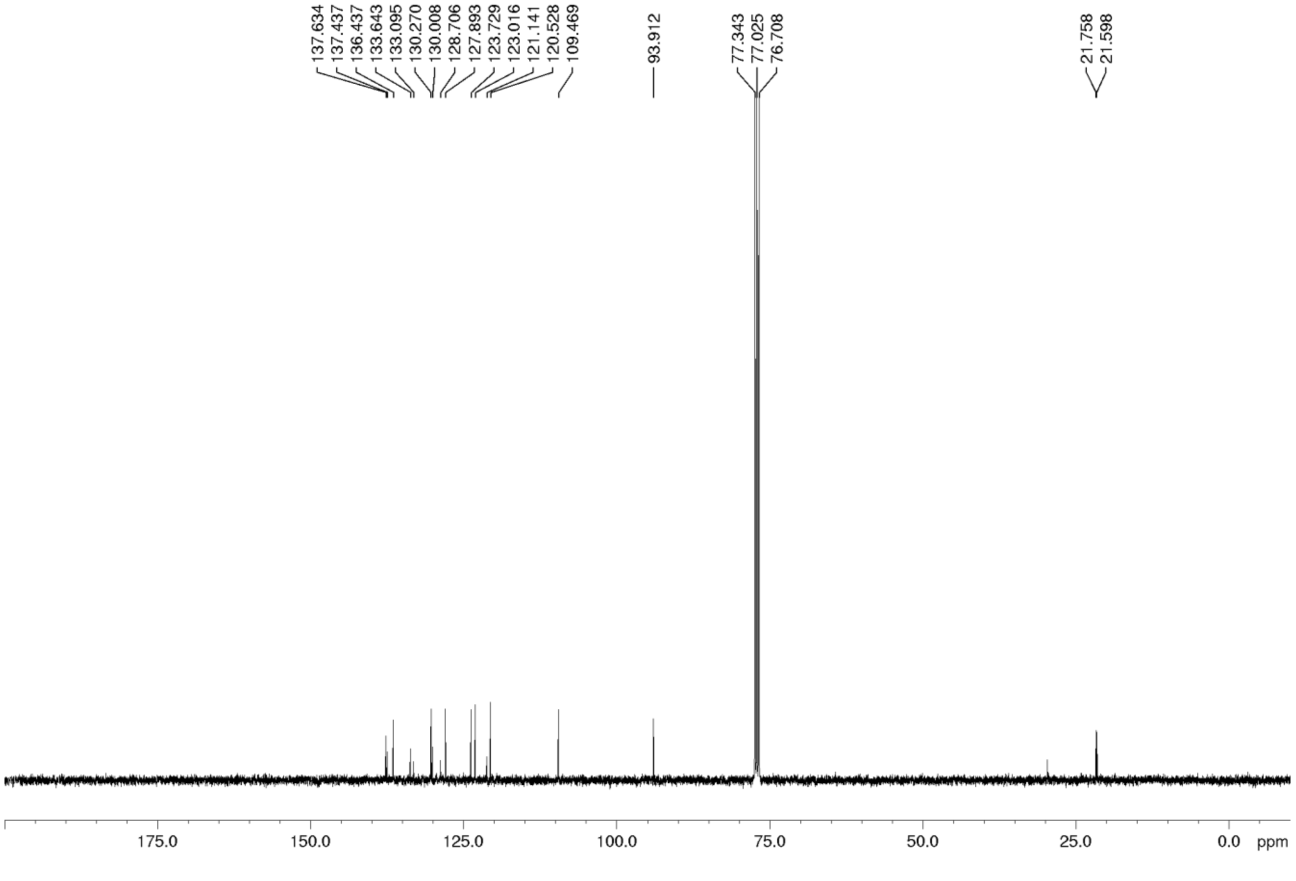
137.634
137.437
136.437
133.643
133.095
130.270
130.008
128.706
127.893
123.729
123.016
121.141
120.528
109.469
93.912
77.343
77.025
76.708
21.758
21.598
175.0
150.0
125.0
100.0
75.0
50.0
25.0
0.0
ppm

13c

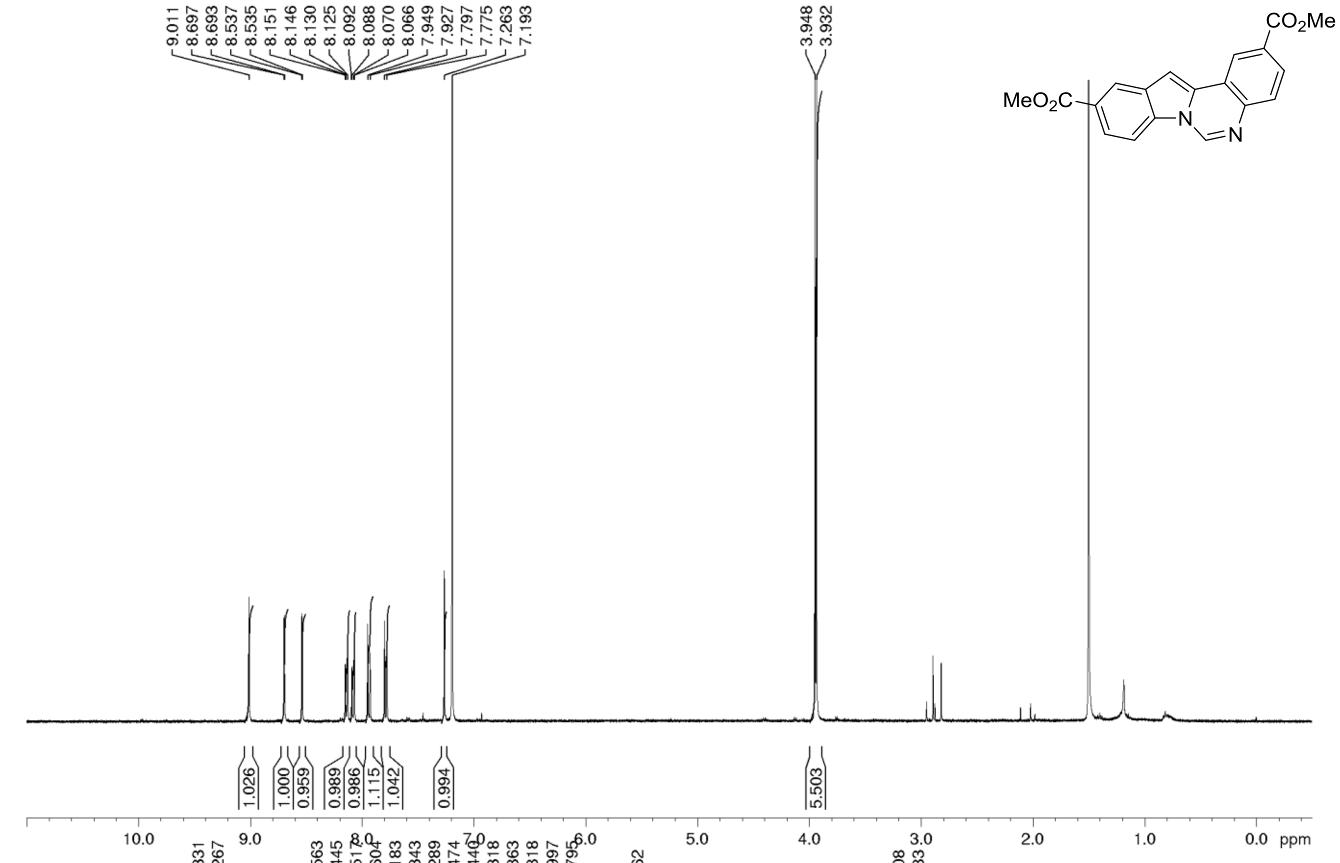
9.011
8.697
8.693
8.537
8.535
8.151
8.146
8.130
8.125
8.092
8.088
8.070
8.066
7.949
7.927
7.797
7.775
7.263
7.193
3.948
3.932
CO_2Me
MeO_2C
N
N
1.026
1.000
0.959
0.989
0.986
1.115
1.042
0.994
5.503
10.0
9.0
8.0
7.0
6.0
5.0
4.0
3.0
2.0
1.0
0.0 ppm

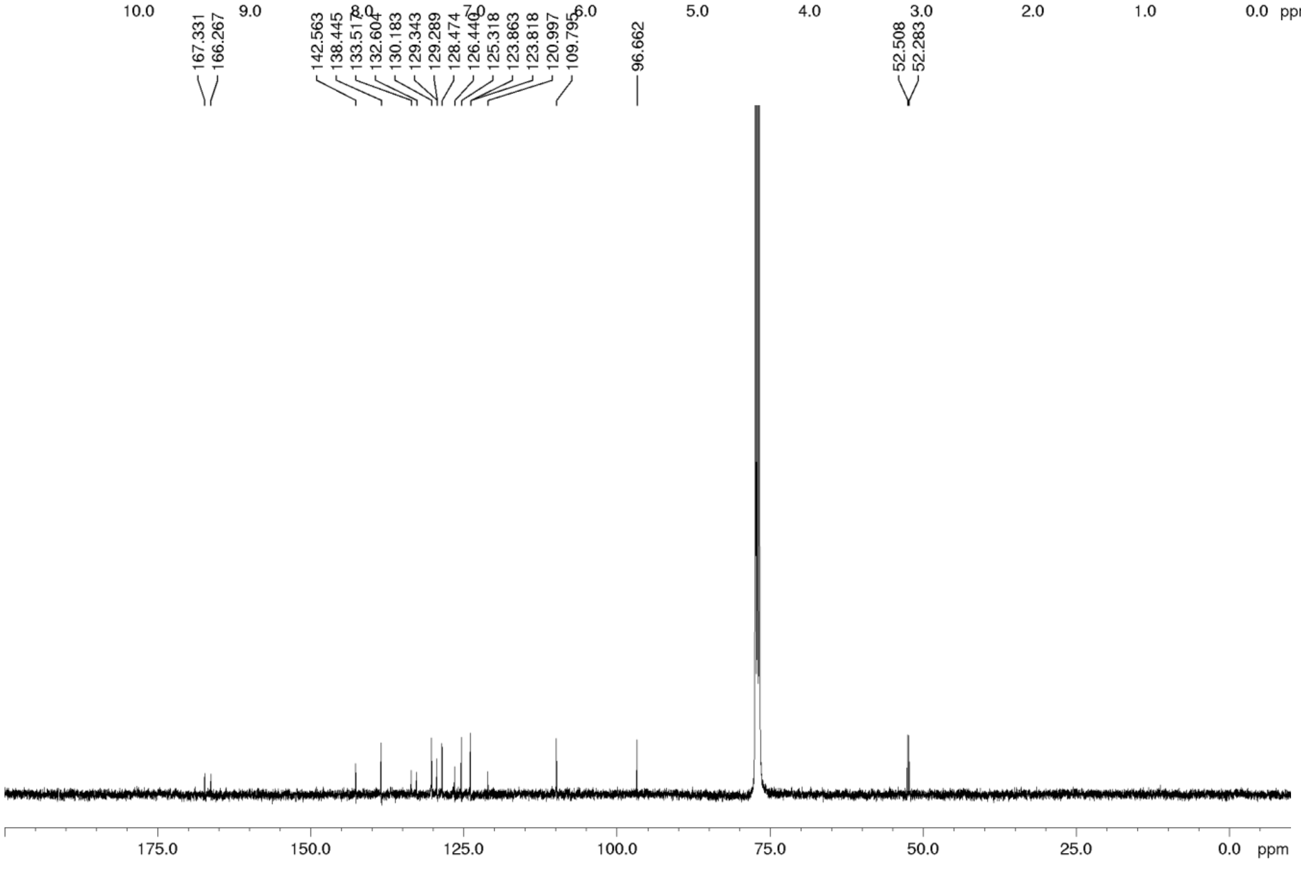
167.331
166.267
142.563
138.445
133.517
132.604
130.183
129.343
129.289
128.474
126.440
125.318
123.863
123.818
120.997
109.795
96.662
52.508
52.283
175.0
150.0
125.0
100.0
75.0
50.0
25.0
0.0 ppm

13d

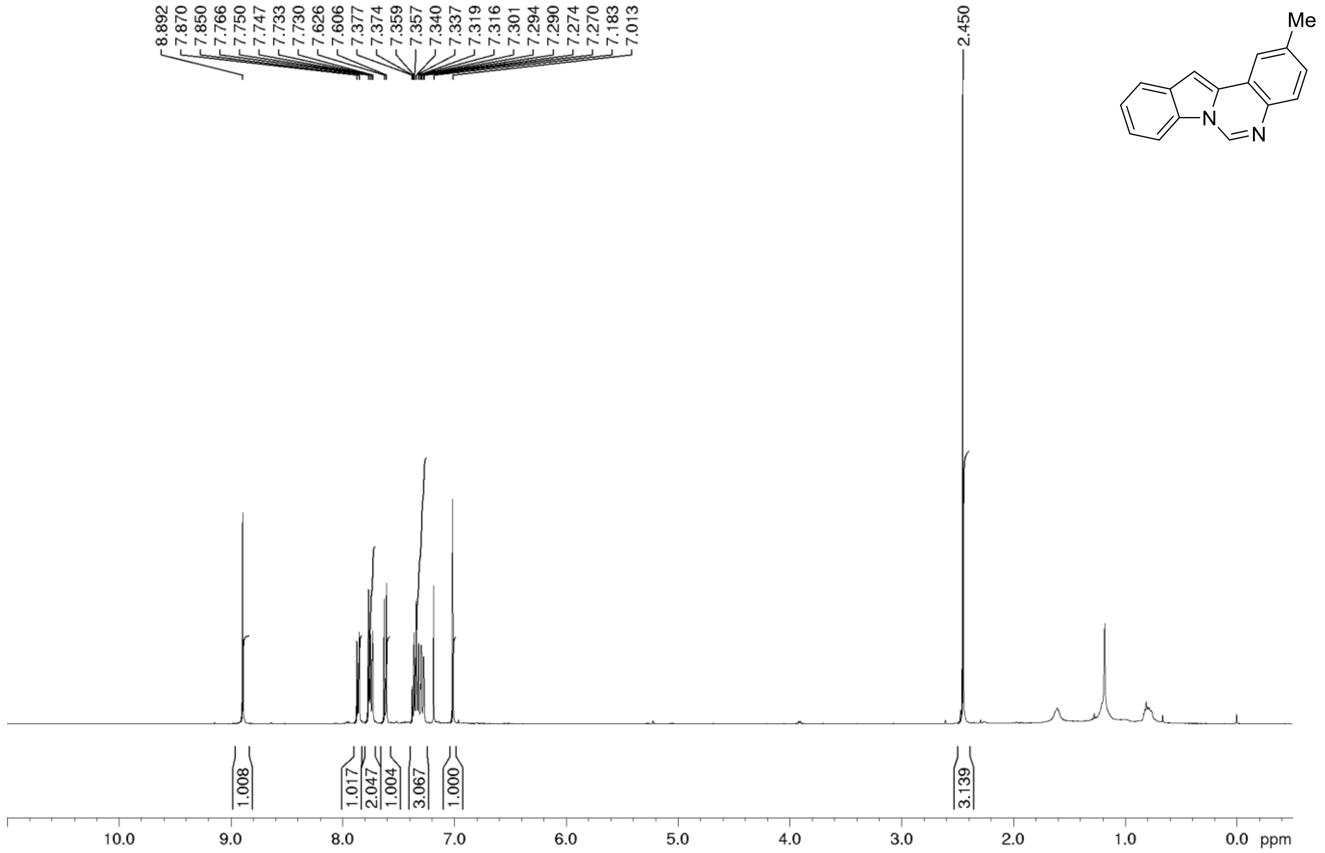
8.892
7.870
7.850
7.766
7.750
7.747
7.733
7.730
7.626
7.606
7.377
7.374
7.359
7.357
7.340
7.337
7.319
7.316
7.301
7.294
7.290
7.274
7.270
7.183
7.013
2.450
Me
N
N
1.008
1.017
2.047
1.004
3.067
1.000
3.139
10.0
9.0
8.0
7.0
6.0
5.0
4.0
3.0
2.0
1.0
0.0 ppm

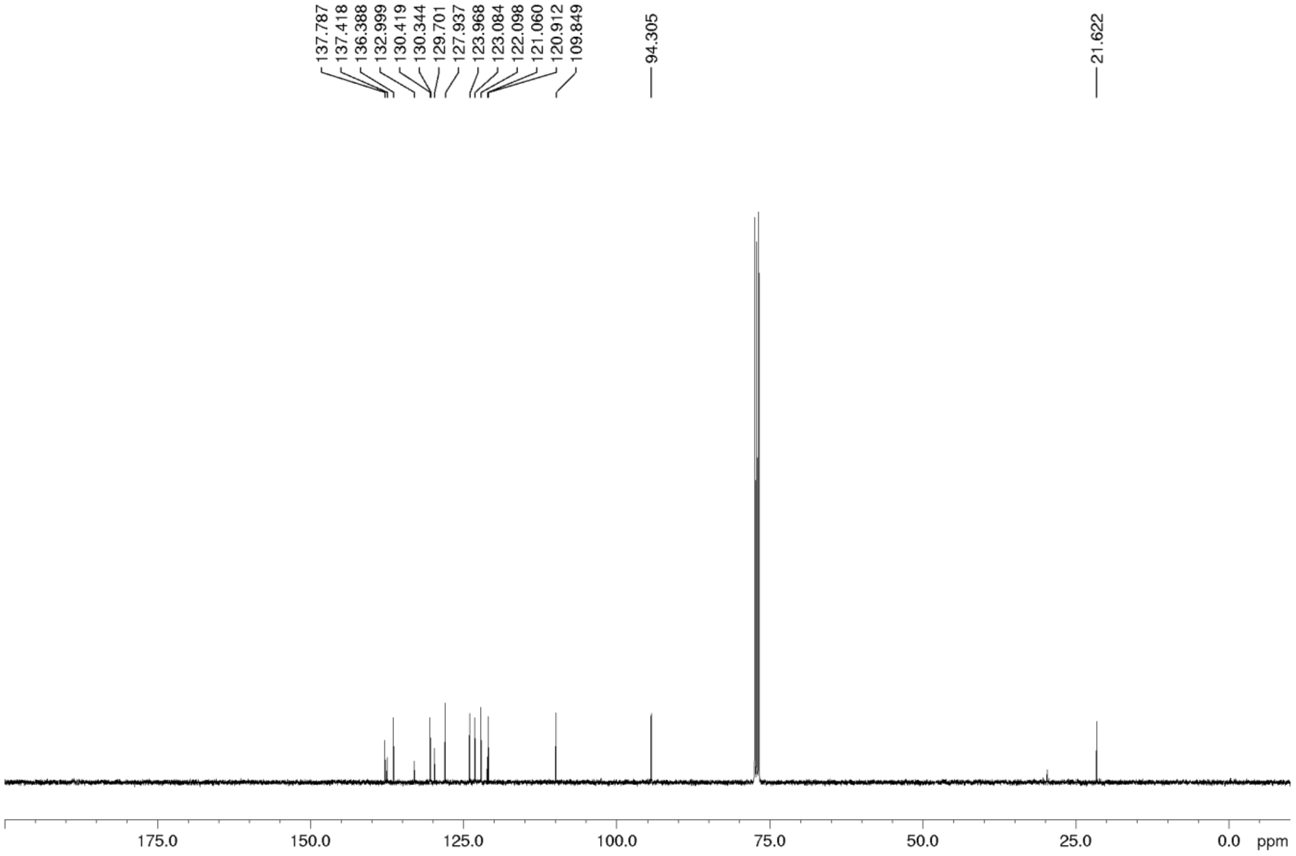
137.787
137.418
136.388
132.999
130.419
130.344
129.701
127.937
123.968
123.084
122.098
121.060
120.912
109.849
94.305
21.622
175.0
150.0
125.0
100.0
75.0
50.0
25.0
0.0 ppm

13e

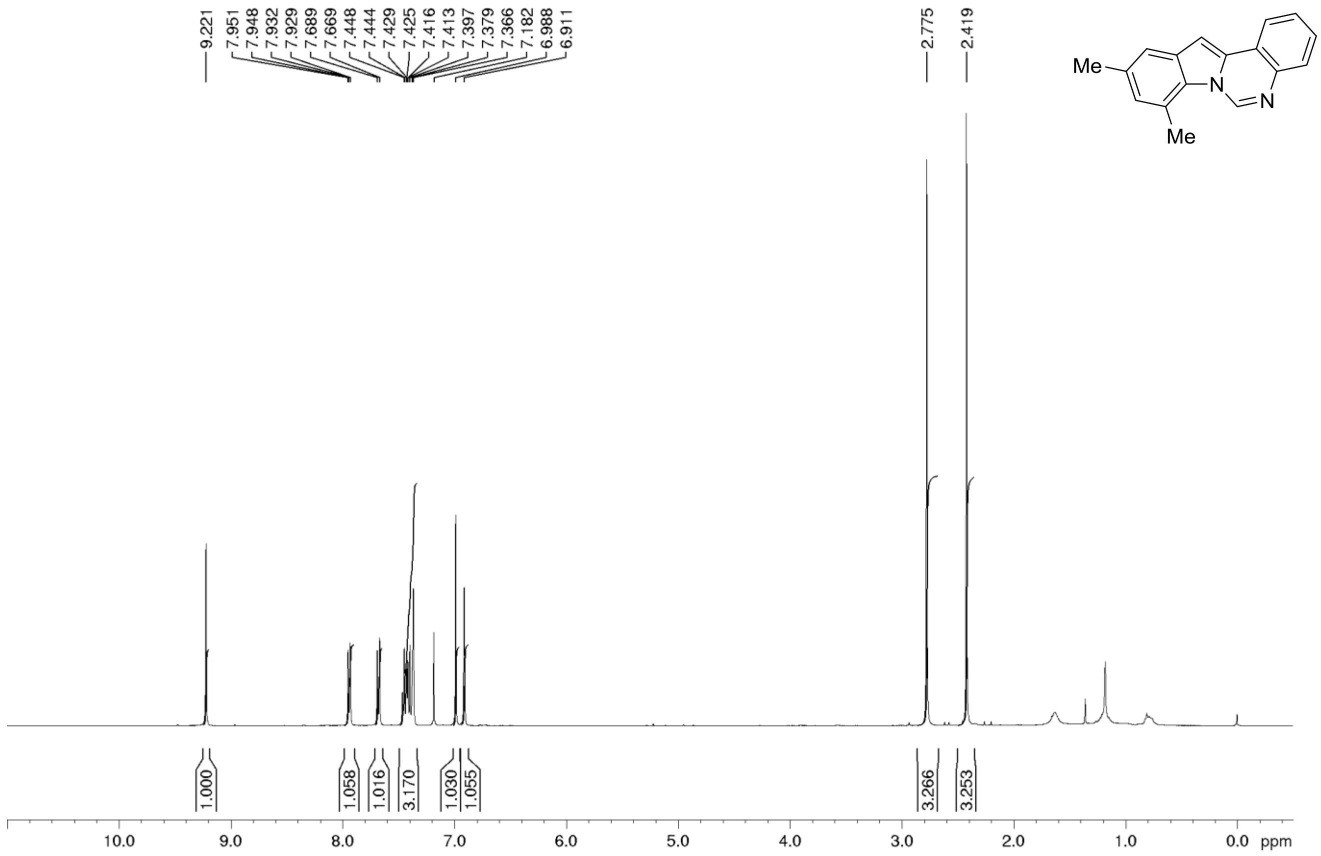
9.221
7.951
7.948
7.932
7.929
7.689
7.669
7.448
7.444
7.429
7.425
7.416
7.413
7.397
7.379
7.366
7.182
6.988
6.911
2.775
2.419
Me
Me
N
N
1.000
1.058
1.016
3.170
1.030
1.055
3.266
3.253
10.0
9.0
8.0
7.0
6.0
5.0
4.0
3.0
2.0
1.0
0.0 ppm

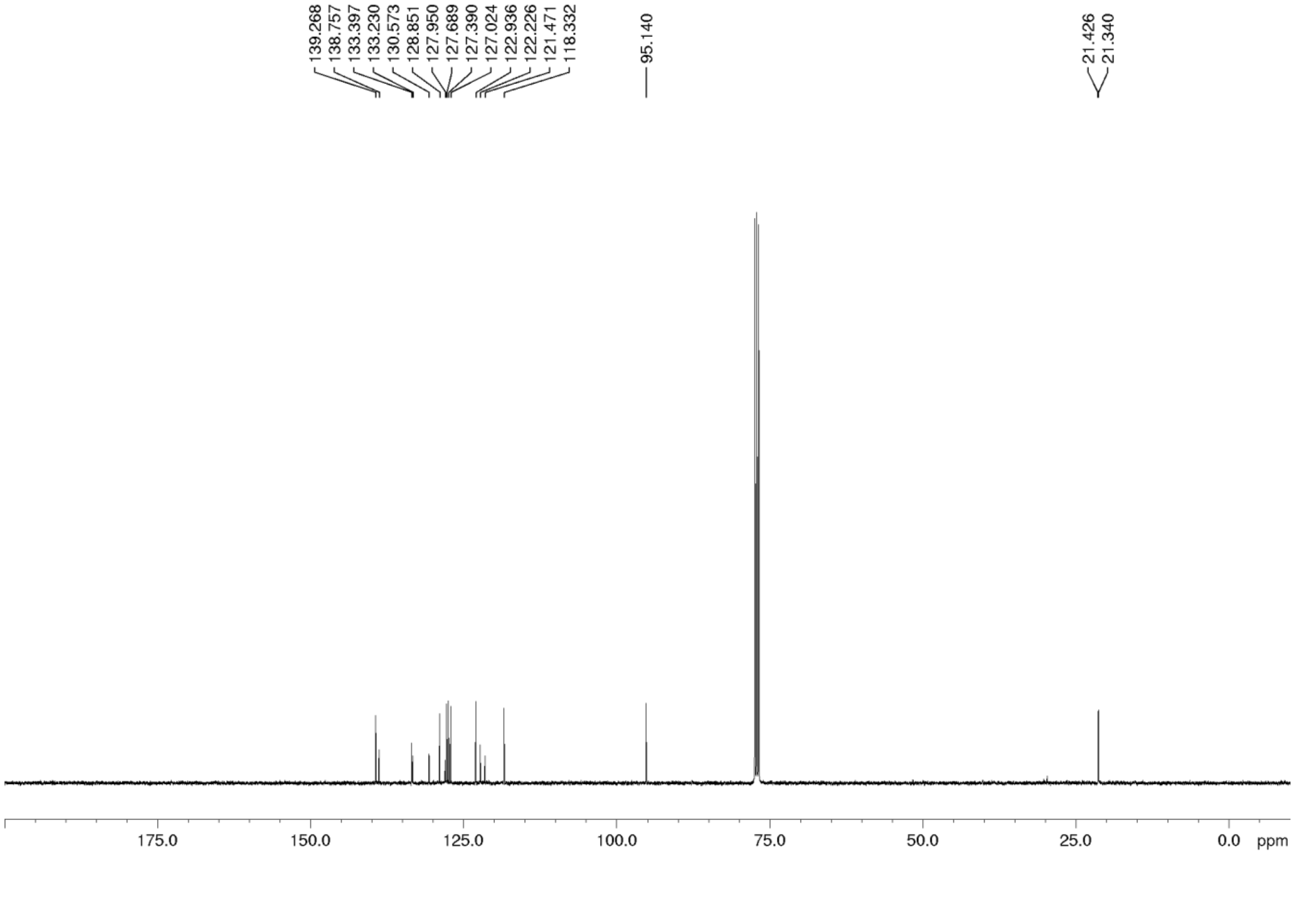
139.268
138.757
133.397
133.230
130.573
128.851
127.950
127.689
127.390
127.024
122.936
122.226
121.471
118.332
95.140
21.426
21.340
175.0
150.0
125.0
100.0
75.0
50.0
25.0
0.0 ppm
